# Low-profile prosthetic foot stiffness category and size, and shoes affect axial and torsional stiffness and hysteresis

**DOI:** 10.3389/fresc.2024.1290092

**Published:** 2024-02-28

**Authors:** Joshua R. Tacca, Zane A. Colvin, Alena M. Grabowski

**Affiliations:** ^1^ Paul M. Rady Department of Mechanical Engineering, University of Colorado, Boulder, CO, United States; ^2^ Department of Integrative Physiology, University of Colorado, Boulder, CO, United States; ^3^ Department of Veterans Affairs, Eastern Colorado Healthcare System, Denver, CO, United States

**Keywords:** prostheses, amputation, prescription, rehabilitation, materials testing

## Abstract

**Introduction:**

Passive-elastic prosthetic feet are manufactured with numerical stiffness categories and prescribed based on the user's body mass and activity level, but mechanical properties, such as stiffness values and hysteresis are not typically reported. Since the mechanical properties of passive-elastic prosthetic feet and footwear can affect walking biomechanics of people with transtibial or transfemoral amputation, characterizing these properties can provide objective metrics for comparison and aid prosthetic foot prescription and design

**Methods:**

We characterized axial and torsional stiffness values, and hysteresis of 33 categories and sizes of a commercially available passive-elastic prosthetic foot model [Össur low-profile (LP) Vari-flex] with and without a shoe. We assumed a greater numerical stiffness category would result in greater axial and torsional stiffness values but would not affect hysteresis. We hypothesized that a greater prosthetic foot length would not affect axial stiffness values or hysteresis but would result in greater torsional stiffness values. We also hypothesized that including a shoe would result in decreased axial and torsional stiffness values and greater hysteresis.

**Results:**

Prosthetic stiffness was better described by curvilinear than linear equations such that stiffness values increased with greater loads. In general, a greater numerical stiffness category resulted in increased heel, midfoot, and forefoot axial stiffness values, increased plantarflexion and dorsiflexion torsional stiffness values, and decreased heel, midfoot, and forefoot hysteresis. Moreover, for a given category, a longer prosthetic foot size resulted in decreased heel, midfoot, and forefoot axial stiffness values, increased plantarflexion and dorsiflexion torsional stiffness values, and decreased heel and midfoot hysteresis. In addition, adding a shoe to the prosthetic foot resulted in decreased heel and midfoot axial stiffness values, decreased plantarflexion torsional stiffness values, and increased heel, midfoot, and forefoot hysteresis.

**Discussion:**

Our results suggest that manufacturers should adjust the design of each category to ensure the mechanical properties are consistent across different sizes and highlight the need for prosthetists and researchers to consider the effects of shoes in combination with prostheses. Our results can be used to objectively compare the LP Vari-flex prosthetic foot to other prosthetic feet to inform their prescription, design, and use for people with a transtibial or transfemoral amputation.

## 1 Introduction

To walk, people with a transtibial or transfemoral amputation typically use passive-elastic prosthetic feet, which are comprised of carbon fiber or fiberglass, and allow elastic energy storage and return during the stance phase. The mechanical properties of passive-elastic prosthetic feet, such as stiffness and hysteresis, affect kinematics, kinetics, muscle activity, metabolic cost, moments acting on the residual limb, and user preference during walking (1–9). Yet, these mechanical properties are not typically reported by the manufacturer. Instead, prosthetic manufacturers use numerical stiffness categories (e.g., 1–9) to delineate each prosthesis where a higher numerical stiffness category corresponds to a stiffer prosthesis and prescribe stiffness categories based on the user's body mass and activity level (10). However, stiffness categories and differences in stiffness values between categories are not consistent across manufacturers or between models (11, 12). Current prosthetic prescriptions rely on manufacturer recommendations and subjective feedback from prosthetists and people with amputation. Therefore, to better inform prosthetic foot prescription, objective values for mechanical properties of prosthetic feet such as stiffness values and hysteresis should be provided. These values will inform dynamic function and can be implemented in future prosthetic designs.

Previous studies have characterized passive-elastic prosthetic feet mechanical properties (11–20) and found that force-displacement and torque-angle profiles are well described by linear (13, 16) or curvilinear (11, 15, 17–19) relationships, which are used to calculate axial (kN/m) and torsional (kN*m/rad) stiffness values. These studies provide axial stiffness values for compressive forces applied to a prosthetic foot heel, midfoot, and forefoot, which affect the biomechanics of the user during walking (1). For example, a study that varied the heel and forefoot stiffness values of an experimental passive-elastic prosthetic foot found that greater heel stiffness values resulted in a higher ground reaction force loading rate, greater knee flexion angle in early stance, and greater knee extension moment, and that greater forefoot stiffness values resulted in a greater knee extension angle in mid-stance and greater knee flexor moment during walking at a range of speeds (0.7–1.5 m/s) for people with unilateral transtibial amputation (1). Therefore, determining the heel, midfoot, and forefoot axial stiffness values for a passive-elastic prosthetic foot would provide objective values for comparing prosthetic feet and better predict the dynamic effects of using different stiffness passive-elastic prosthetic feet on walking biomechanics.

Characterizing torsional stiffness values of passive-elastic prosthetic feet can provide additional information to derive function and can be compared to the biological ankle-foot system. A biological ankle can behave mechanically like a torsional spring and damper system during walking at 1.2 m/s (21) and typically has a curvilinear torque vs. angle relationship during the stance phase so that the torsional stiffness increases with greater ankle dorsiflexion (concave shape) (21, 22). Some previous studies have characterized torsional prosthetic foot stiffness properties (6, 16, 22–24). One method of characterizing the torsional stiffness of prosthetic feet is to calculate the average torsional stiffness when dorsiflexion and plantarflexion torques are applied to the prosthesis by a materials testing machine (16). Another method to characterize the torsional stiffness of prosthetic feet is to measure the ankle torque and angle during the stance phase of walking and determine how well or if the torque-angle curve matches the concave shape of a biological ankle torque-angle curve (i.e., Index of Anthromorphicity) (22). In addition, another previous study found that prosthetic foot length affects torsional stiffness values since longer feet have a longer moment arm and have greater torsional stiffness values than shorter feet for a given applied force and angle change (24).

Furthermore, the prosthetic foot energy returned during the push-off phase of stance depends on stiffness values (3) and hysteresis, or energy loss (17, 25). Passive-elastic prosthetic foot energy return is related to the energy stored and can affect walking biomechanics, where lower energy return can result in decreased affected leg work during push-off, increased unaffected leg work during collision, and increased hip work (7, 26). Hysteresis has been reported for some passive-elastic prosthetic feet (13, 17–19) and likely depends more on material properties rather than stiffness categories of prosthetic feet (13). Ultimately, characterizing the passive-elastic prosthetic feet axial and torsional stiffness values and hysteresis will better inform prosthetic prescription and function by allowing objective comparisons between different prosthetic foot models, stiffness categories, and sizes.

Most people with a transtibial or transfemoral amputation wear shoes over their prosthetic foot during walking and this likely affects stiffness values and hysteresis compared to prosthetic feet alone (27). Major et al. found that adding shoes to prosthetic feet resulted in lower axial stiffness values at the heel and midfoot but not at the forefoot compared to prosthetic feet alone (27). Moreover, Major et al. found that adding shoes to prosthetic feet resulted in greater hysteresis compared to prosthetic feet alone (27). Since adding shoes to prosthetic feet changes the mechanical properties compared to prosthetic feet alone and because shoes are commonly used when people with a transtibial or transfemoral amputation walk, shoes should be considered when characterizing prosthetic feet mechanical properties.

There are many prosthetic foot models that are commercially available to people with a transtibial or transfemoral amputation. One such model is the Össur low-profile (LP) Vari-flex (Össur, Reykjavik, Iceland), which is a passive-elastic prosthetic foot made of carbon-fiber with a short build height (0.068 m) (10) so that it can be used by people with long residual limbs. It has also been used within a stance-phased powered prosthesis (28). Characterizing the mechanical properties of LP Vari-flex feet can provide objective measures that can be used to compare these prostheses to other available prosthetic feet (11–20), inform dynamic function, and influence future prosthetic design that includes stance-phase powered prostheses. Therefore, it is important to determine the axial and torsional stiffness values and hysteresis of a wide range of different stiffness categories and sizes of prosthetic feet. Moreover, since shoes can affect the mechanical properties of prosthetic feet, it is also important to determine the axial and torsional stiffness values and hysteresis of prosthetic feet with and without shoes.

The Össur Vari-flex (higher profile version of the LP Vari-flex, Össur, Reykjavik, Iceland) prosthetic foot exhibits a curvilinear force-displacement profile (11), thus we expected that the axial and torsional stiffness of all the stiffness categories of the LP Vari-flex would be better characterized by a curvilinear force-displacement profile than a linear force-displacement profile independent of a shoe. We assumed that because a greater numerical stiffness category is prescribed to people with greater body mass and higher activity levels, a greater stiffness category would result in higher axial stiffness values when force is applied at the heel, midfoot, and forefoot, and higher torsional stiffness values when plantarflexion and dorsiflexion torque are applied to the prosthetic foot but would have no effect on hysteresis with or without a shoe. Since manufacturers recommend the same LP Vari-flex prosthetic foot stiffness category for a given body mass and activity level regardless of prosthetic foot size, we hypothesized that greater passive-elastic prosthetic foot length (size) would have no effect on axial stiffness values when force is applied at the heel, midfoot, and forefoot, and hysteresis within a given category with or without a shoe. However, since increasing prosthetic foot size increases the length and moment arms of the prosthesis, we hypothesized that greater passive-elastic prosthetic foot length (size) would result in greater torsional stiffness values when plantarflexion and dorsiflexion torque are applied to the prosthetic foot with or without a shoe. Based on results from a previous study that found adding a shoe to prosthetic feet resulted in lower axial stiffness values at the heel and midfoot but not at the forefoot (27), we hypothesized that adding a shoe to the prosthetic foot would result in lower axial stiffness values when force is applied at the heel and midfoot and increase hysteresis but not affect axial stiffness values when force is applied at the forefoot or torsional stiffness values when plantarflexion and dorsiflexion torque are applied to the prosthetic foot and shoe compared to without a shoe.

## 2 Methods

### 2.1 Prosthetic feet

LP Vari-flex prosthetic feet are manufactured in a range of different stiffness categories (1–9) and foot sizes (22–30 cm) that are prescribed to people with a transtibial or transfemoral amputation who have a range of body mass (45–166 kg) and low to high impact (activity) levels (10). We determined the axial stiffness (kN/m) values, torsional stiffness (N*m/rad) values, and hysteresis (%) of 33 different LP Vari-flex prosthetic feet with different stiffness categories (Categories 1–8) and sizes (24–29 cm; Table 1) in compression using a materials testing machine (MTM; Instron Series 5859, Norwood, MA). We determined axial stiffness values and hysteresis with a force applied at the heel, midfoot, and forefoot of each prosthetic foot including the rubber cosmesis with and without a standard walking shoe (New Balance MA411, Boston, MA). Then, we determined torsional stiffness values when plantarflexion and dorsiflexion torque were applied to each prosthetic foot including the rubber cosmesis with and without a standard walking shoe (New Balance MA411, Boston, MA). For two prosthetic foot sizes (27 and 29), we did not have a New Balance MA411 shoe, so we instead used New Balance MW928 shoes. Both the New Balance MA411 and MW928 are designed for walking and have similar mass and construction. Thus, we assumed that the mechanical effects of these walking shoes would not differ.

**Table 1:** Low-profile Vari-flex prosthetic foot (10) stiffness category, size, shoe, average manufacturer recommended body mass for a moderate impact level, maximum manufacturer recommended body mass for a moderate impact level, 1.3 times the maximum recommended body weight (BW; maximum force threshold for the heel and midfoot tests), and 1.0 times the maximum recommended BW (maximum force threshold for the forefoot test).

Foot	Stiffness category	Size (cm)	Shoe (US Size)	Average body mass (kg)	Maximum body mass (kg)	1.3 BW (N)	1.0 BW (N)
1	1	24	MA411 (7)	48.5	52	662	510
2	1	25	MA411 (8)	48.5	52	662	510
3	1	26	MA411 (9)	48.5	52	662	510
4	2	24	MA411 (7)	56	59	752	578
5	2	25	MA411 (8)	56	59	752	578
6	2	26	MA411 (9)	56	59	752	578
7	3	24	MA411 (7)	64	68	866	666
8	3	25	MA411 (8)	64	68	866	666
9	3	26	MA411 (9)	64	68	866	666
10	3	28	MA411 (11)	64	68	866	666
11	3	29	MW928 (12.5)	64	68	866	666
12	4	24	MA411 (7)	73	77	981	755
13	4	25	MA411 (8)	73	77	981	755
14	4	26	MA411 (9)	73	77	981	755
15	4	27	MW928 (10)	73	77	981	755
16	4	28	MA411 (11)	73	77	981	755
17	4	29	MW928 (12.5)	73	77	981	755
18	5	24	MA411 (7)	83	88	1,121	862
19	5	25	MA411 (8)	83	88	1,121	862
20	5	26	MA411 (9)	83	88	1,121	862
21	5	27	MW928 (10)	83	88	1,121	862
22	5	28	MA411 (11)	83	88	1,121	862
23	5	29	MW928 (12.5)	83	88	1,121	862
24	6	25	MA411 (8)	94.5	100	1,274	980
25	6	26	MA411 (9)	94.5	100	1,274	980
26	6	27	MW928 (10)	94.5	100	1,274	980
27	6	28	MA411 (11)	94.5	100	1,274	980
28	6	29	MW928 (12.5)	94.5	100	1,274	980
29	7	26	MA411 (9)	108.5	116	1,478	1,137
30	7	27	MW928 (10)	108.5	116	1,478	1,137
31	7	28	MA411 (11)	108.5	116	1,478	1,137
32	7	29	MW928 (12.5)	108.5	116	1,478	1,137
33	8	27	MW928 (10)	123.5	130	1,656	1,274

### 2.2 Axial stiffness

**Figure 1:**
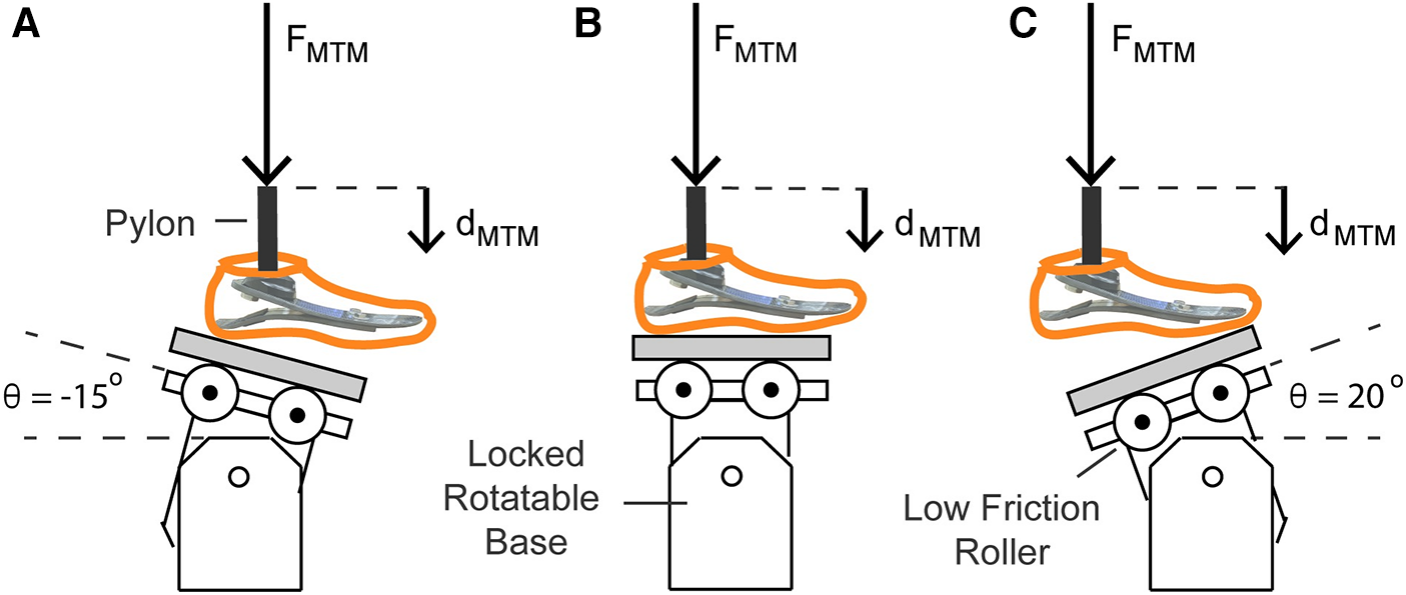
Illustration of axial stiffness testing for the (**A**) heel, (**B**) midfoot, and (**C**) forefoot of each prosthetic foot. The materials testing machine (MTM) applied force (*F*_MTM_) vertically at 100 N/s along the pylon to compress the prosthetic foot. *F*_MTM_ and vertical displacement (*d*_MTM_) were measured by the load cell and MTM. A low friction roller was placed beneath the prosthetic foot to minimize the torque applied on the load cell of the MTM. For the axial tests on the heel, midfoot, and forefoot, the rotatable base was locked at −15°, 0°, and 20° relative to horizontal, respectively.

We constructed a custom aluminum base and low-friction roller system for the MTM to measure the heel, midfoot, and forefoot axial stiffness values of the prosthetic feet. We used a low friction roller between the base and each prosthetic foot to minimize torque on the uniaxial load cell of the MTM (Figure 1). A rigid pylon was aligned vertically and attached to the MTM. Each prosthetic foot was attached to the rigid pylon and the bottom of the prosthetic foot was aligned perpendicular to the pylon. We set the base at −15°, 0°, and 20° relative to horizontal, which corresponds to the angles required for heel, midfoot, and forefoot axial stiffness testing, respectively (29) (Figure 1). For each test, we preloaded the prosthetic foot with 4–6 N so that the platform (grey in Figure 1) would not slide out between the low friction roller and prosthetic foot in between each cycle and used the MTM to apply a force along the pylon at 100 N/s (29) for four consecutive compressive loading and unloading cycles. Each prosthetic foot stiffness category is recommended for a user within a range of body mass values by the manufacturer (Table 1). For each prosthetic stiffness category, we used the highest body mass within the recommended range to estimate the peak ground reaction force applied on the heel, midfoot, and forefoot of the prosthetic foot during walking. We set the maximum force of each test to a value based off the first and second peak vertical ground reaction forces on the affected leg of a person with a transtibial amputation walking on level ground at 1.75 m/s to estimate the ground reaction force that could be applied to a particular prosthesis during walking (30). When a person with a transtibial amputation walks on level ground at 1.75 m/s using a passive-elastic prosthesis, they apply a first peak vertical ground reaction force that is 1.3 times their body weight (BW) for their affected leg (30). Thus, we applied a maximum force of 1.3 times BW of the heaviest person within the recommended range for each prosthetic stiffness category at a moderate impact level for the heel and midfoot tests (base at −15° and 0°). When a person with a transtibial amputation walks on level ground at 1.75 m/s using a passive-elastic prosthesis, they apply a second peak vertical ground reaction force that is 1.0 times their body weight (BW) for their affected leg (30). Thus, we applied a maximum force of 1.0 times BW of the heaviest person within the recommended range for each prosthetic stiffness category at a moderate impact level for the forefoot test (base at 20°).

**Figure 2:**
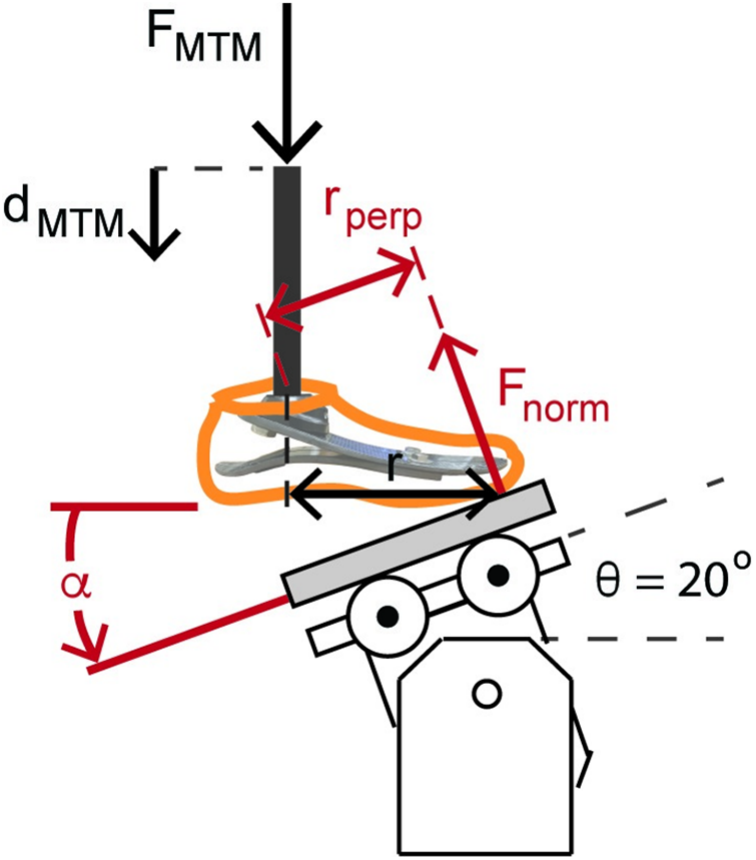
Illustration of the forefoot prosthetic axial stiffness testing and dorsiflexion torsional stiffness testing. The MTM applied force (*F*_MTM_) and displacement (*d*_MTM_) vertically along the pylon. The reaction force applied to the bottom of the prosthetic foot is the normal force (*F*_norm_) relative to the base. For the heel, midfoot, and forefoot axial stiffness testing, the rotatable base was locked at −15°, 0°, and 20° relative to horizontal, respectively. *F*_norm_ equals *F*_MTM_/cos(*θ*). The displacement of the prosthetic foot normal to the base (*d*_norm_) equals *d*_MTM_ × cos(*θ*). Therefore, axial prosthetic stiffness (*k*_pros_) equals the quotient of *F*_MTM_ and *d*_MTM_ × cos(*θ*)^2^. We estimated torsional stiffness values from the quotient of the product of *F*_norm_ and the perpendicular moment arm (*r*_perp_ = *r* × cos(*θ*)) and the angular displacement of the foot $\left(\alpha =\;\tan^{-1}\left(\frac{d_{\mathrm{MTM}}}{r}\right)\right)$.

We determined the axial stiffness values of each prosthetic foot as the quotient of the normal force and displacement applied by the base onto the bottom of the prosthesis (Figures 1, 2). The normal force (*F*_norm_) equals the quotient of the *F*_MTM_ and the cosine of the angle of the base relative to the prosthetic foot (θ) (Figures 1, 2; Equation 1):

(1) $$F_{\mathrm{norm}}=\frac{F_{\mathrm{MTM}}}{\cos(\theta )}$$

The displacement of the prosthetic foot normal to the base (*d*_norm_) equals the product of the vertical displacement of the materials testing machine (*d*_MTM_) and the cosine of the angle of the base relative to the prosthetic foot (θ) (Figures 1, 2; Equation 2):

(2) $$d_{\mathrm{norm}}=d_{\mathrm{MTM}}\cos(\theta )$$

Therefore, the axial stiffness value of the prosthetic foot (*k*_pros_) equals *F*_MTM_ divided by the product of *d*_MTM_ and cos $(\theta {)}^{2}$ (Equation 3):

(3) $$k_{\mathrm{pros}}=\frac{F_{\mathrm{norm}}}{d_{\mathrm{norm}}}=\frac{F_{\mathrm{MTM}}}{d_{\mathrm{MTM}}\cos{\left(\theta \right)}^{2}}$$

### 2.3 Torsional stiffness

We determined torsional stiffness values by dorsiflexing and plantarflexing the prosthetic feet. Plantarflexion and dorsiflexion torsional stiffness values of each prosthetic foot were measured as the quotient of the torque and angular displacement of the prosthesis calculated from the force and displacement measured during the heel and forefoot axial stiffness tests when the rotatable base was locked at −15° and 20°, respectively (Figure 1). For plantarflexion torsional stiffness, we estimated the moment arm as the horizontal distance between the point of contact of the heel during the heel axial stiffness test and the pylon (*r*) and multiplied it by cosine of the base angle (−15°) to calculate the perpendicular moment arm (*r*_perp_). The point of contact was the location where the heel of the prosthesis contacted the base when the prosthetic foot was preloaded with 4–6 N. For dorsiflexion torsional stiffness, we estimated the moment arm as the horizontal distance between the point of contact of the forefoot during the forefoot axial stiffness test and the pylon (*r*; Figure 2) and multiplied it by the cosine of the base angle (20°) to calculate the perpendicular moment arm (*r*_perp_; Figure 2). The point of contact was the location where the forefoot of the prosthesis contacted the base when the prosthetic foot was preloaded with 4–6 N and corresponded with the start of the forefoot axial stiffness test. We calculated torque throughout compression as the product of the normal force (*F*_norm_) and *r*_perp_ (Figure 2). We assumed the point of contact and thus *r*_perp_ was constant throughout loading and unloading due in part to the low friction roller placed beneath the prosthesis. We calculated the angle of the prosthetic foot (*α*) as the inverse tangent of the vertical displacement of the MTM (*d*_MTM_) divided by the horizontal distance of the point of contact and the pylon (*r*; Figure 2). Thus, torsional stiffness $\left(k_{\mathrm{pros},\mathrm{torsion}}\right)$ equals the quotient of the change in torque (τ) and angle (α in rad) of the prosthetic foot (Equation 4):

(4) $$k_{\mathrm{pros},\mathrm{torsion}}=\frac{\tau }{\alpha }=\frac{F_{\mathrm{norm}}\times r_{\mathrm{perp}}}{\tan^{-1}\left(\frac{d_{\mathrm{MTM}}}{r}\right)}$$

### 2.4 Hysteresis

We calculated hysteresis for each loading and unloading cycle as the percentage of energy lost during unloading (difference between the energy returned during unloading and the energy stored during loading) compared to the energy stored during loading. Hysteresis was calculated as the quotient of the difference in the area under the loading and unloading curves and the area under the loading curve (Equation 5):

(5) $$\mathrm{Hysteresis}=\frac{\int_{0}^{\max d_{\mathrm{norm}}}F_{\mathrm{norm}}(d_{\mathrm{norm}})dd_{\mathrm{norm}}-\;\int_{\max d_{\mathrm{norm}}}^{0}F_{\mathrm{norm}}(d_{\mathrm{norm}})dd_{\mathrm{norm}}}{\int_{0}^{\max d_{\mathrm{norm}}}F_{\mathrm{norm}}(d_{\mathrm{norm}})dd_{\mathrm{norm}}}\times 100\text{\%}$$

where *F*_norm_ is the normal force, *d*_norm_ is the displacement of the prosthetic foot, and *dd*_norm_ is the differential of the displacement of the prosthetic foot.

### 2.5 Data analysis

We used a custom MATLAB script (Mathworks Inc., Natick, MA, USA) to fit linear and quadratic curves to the force-displacement and torque-angle data, calculated average axial and torsional stiffness values, and calculated hysteresis. We used a 20 N *F*_norm_ threshold to define the start and end of each loading and unloading cycle and set the maximum *F*_norm_ or torque value of each cycle as the end of the loading phase of the cycle. Then, we fit linear and quadratic least-squares curves to the normal force-displacement and torque-angle data from the loading phases of the last three cycles for each prosthetic foot at the heel, midfoot, and forefoot. So that our results are comparable to previous studies that characterized Vari-flex prosthetic feet (the higher profile version of the LP Vari-flex prosthesis) (11, 20), we calculated average axial and torsional stiffness values from the discrete value of the slope of the force-displacement and torque-angle curve from a minimum value of 50 N to 1.0 × body weight (BW) for the average body mass recommended for the moderate impact level (Table 1). We averaged that value for the last three test cycles for each foot and test condition. Finally, we averaged the hysteresis from the last three cycles from the normal force-displacement and torque-angle data for each prosthetic foot and test condition.

### 2.6 Statistical analysis

We calculated adjusted *R*^2^ values (31, 32) for the linear and quadratic curves for each prosthetic foot at the heel, midfoot, and forefoot. We used adjusted R^2^ values because the adjusted *R*^2^ corrects for added degrees of freedom in the model and allows comparison of the goodness of fit between the linear and quadratic curves (31, 32). The axial and torsional stiffness of the prosthetic foot was determined to be better characterized by a linear or quadratic force-displacement or torque-angle curve if the adjusted *R*^2^ was greater. Then, we constructed eight linear regression models (33) to determine the effect of prosthetic foot stiffness category, prosthetic foot size, and shoe or no shoe on the average axial stiffness values and hysteresis at the heel, midfoot, and forefoot, and torsional stiffness values in the plantarflexion and dorsiflexion directions. We set average axial stiffness values, torsional stiffness values, or hysteresis as the dependent variable and stiffness category (numerical; 1–8), size (numerical; 24–27 cm), and shoe vs. no shoe (categorical; shoe = 1, no shoe = 0) as independent variables (dependent variable = intercept + *B*_1_ × stiffness category + *B*_2_ × size + *B*_3_ × shoe/no shoe). We report unstandardized model coefficients *B*_1_, *B*_2_, and *B*_3_, which represent the change in dependent variable (average axial stiffness value, average torsional stiffness value, and hysteresis at the heel, midfoot, or forefoot) corresponding to a 1 category change in stiffness category, 1 cm change in size, and use of a shoe compared to no shoe, respectively. For each comparison, we controlled for the remaining fixed effects. We visually inspected regression model assumptions of linearity, normality, and homoscedasticity (34), and report 95% confidence intervals for each model coefficient and *R*^2^ values for each regression model. A unit change in hysteresis (%) is a percentage point (p.p.) where one p.p. refers to a 1% unit, such that an increase from 5% to 6% is a 1 p.p. increase as opposed to a 20% increase (i.e., *not* $\frac{6\text{\%}-5\text{\%}}{5\text{\%}}\times 100\text{\%}=20\text{\%}$). We used a significance level of *p* < 0.05. All statistical analyses were performed in RStudio (Boston, MA, USA).

## 3 Results

**Figure 3:**
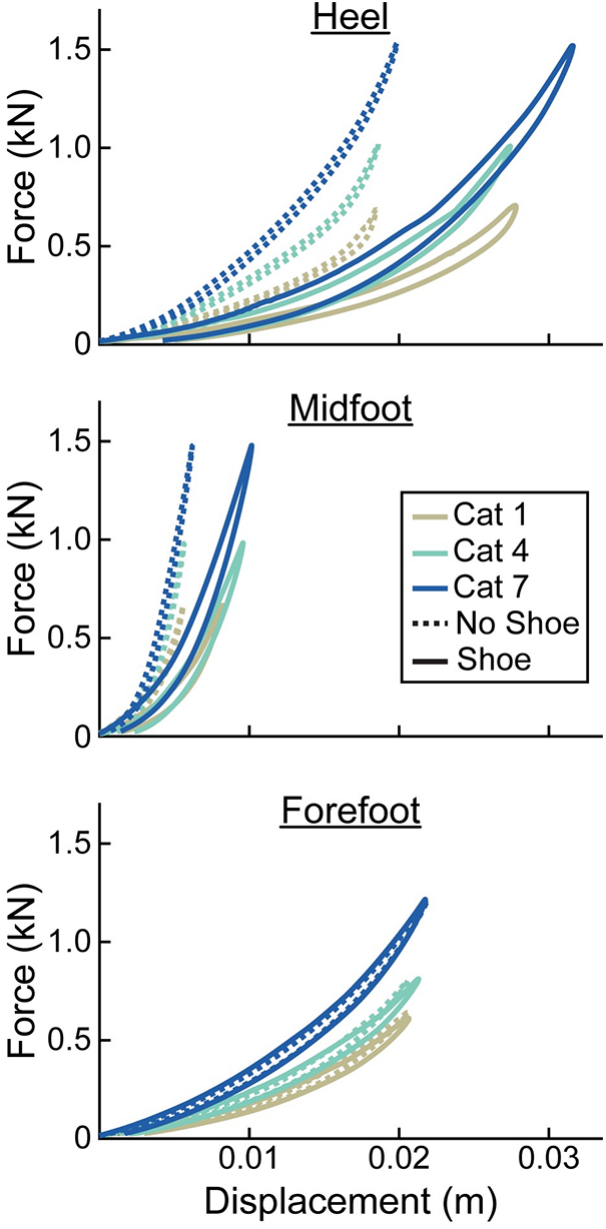
Representative force (kN) vs. displacement (m) curves of the heel, midfoot, and forefoot of size 26 cm LP Vari-flex prosthetic feet. The colors represent different stiffness categories (categories 1, 4, 7). The dashed lines are for the tests without a shoe and the solid lines are for the tests with the shoe. Curves go in a clockwise direction from the start to the end of a cycle.

**Figure 4:**
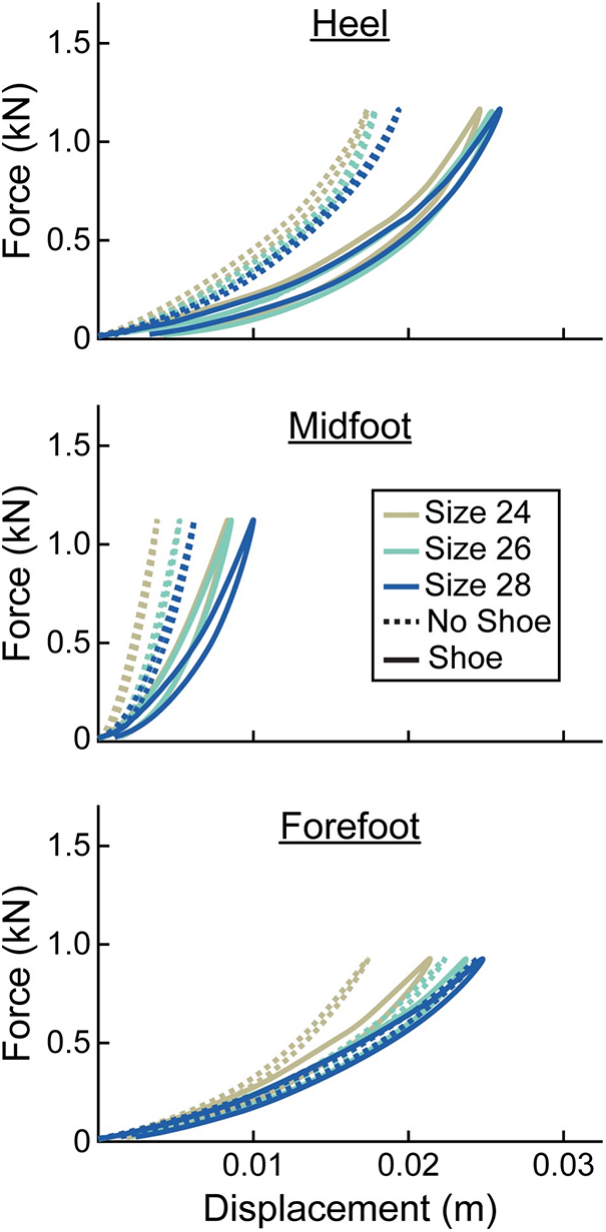
Representative force (kN) vs. displacement (m) curves of the heel, midfoot, and forefoot of stiffness category 5 LP Vari-flex prosthetic feet. The colors represent different sizes (24 cm, 26 cm, 28 cm). The dashed lines are for the tests without a shoe and the solid lines are for the tests with the shoe. Curves go in a clockwise direction from the start to the end of a cycle.

For every prosthetic foot, we found that the adjusted *R*^2^ was higher when force vs. displacement was represented as a quadratic compared to a linear curve (average adjusted *R*^2^ across all tests–quadratic: 1.00, linear: 0.95). Therefore, prosthetic foot force-displacement curves were better described by a quadratic compared to linear fit. The prosthetic foot force-displacement curves were well described by a progressive, quadratic force-displacement curve, meaning that axial stiffness increased with greater force applied (Figures 3, 4).

**Figure 5:**
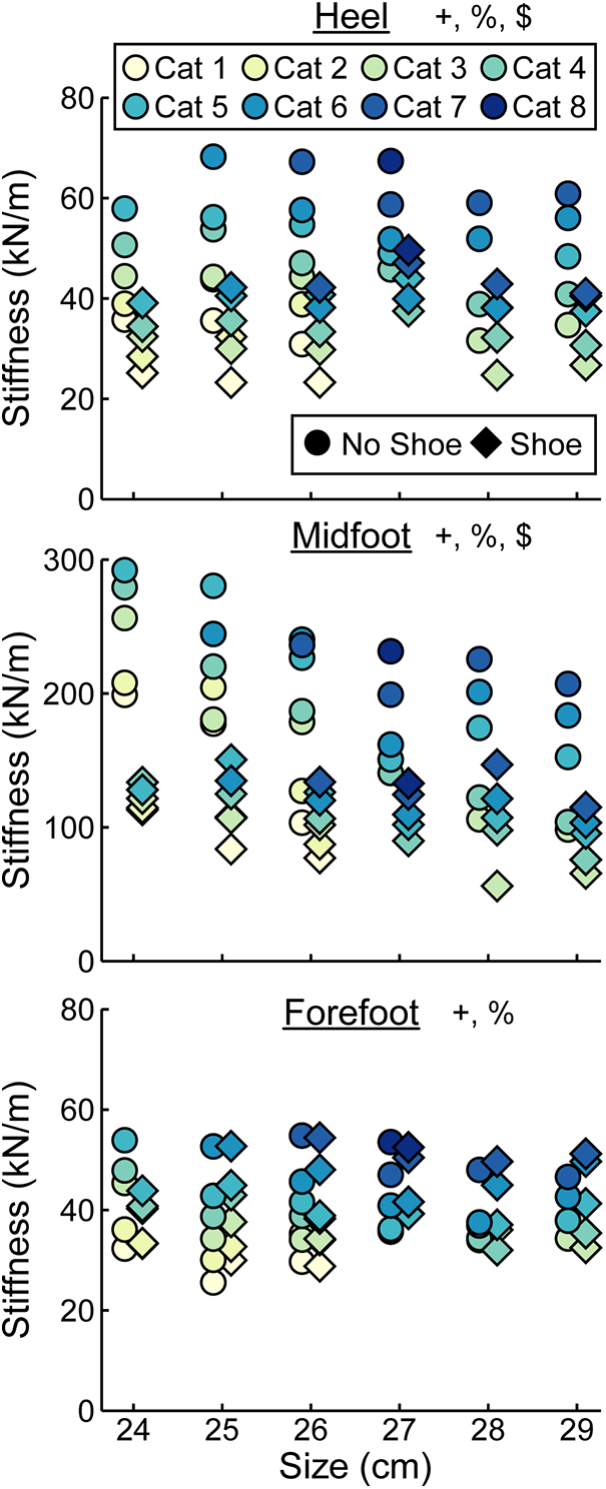
Average axial stiffness values (kN/m) vs. LP Vari-flex prosthetic foot size in cm. The colors represent different stiffness categories (categories 1–8), the circles represent average axial stiffness values without a shoe, and the diamonds represent average axial stiffness values with a shoe. Symbols are offset for no shoe and shoe for clarity. The *y*-axis differs for the midfoot compared to heel and forefoot axial stiffness values. + indicates a significant effect of stiffness category, % indicates a significant effect of size, and $ indicates a significant effect of shoe.

At the heel, average prosthetic foot axial stiffness values increased by 4.6 kN/m for every 1 stiffness category increase (*p* < 0.001), decreased by 1.7 kN/m for every 1 cm increase in size (*p* < 0.001), and decreased by 13.5 kN/m with the shoe compared to without the shoe (*p* < 0.001; Figure 5; Table 2). At the midfoot, average prosthetic foot axial stiffness values increased by 15.6 kN/m for every 1 stiffness category increase (*p* < 0.001), decreased by 19.4 kN/m for every 1 cm increase in size (*p* < 0.001), and decreased by 81.4 kN/m with the shoe compared to without the shoe (*p* < 0.001; Figure 5; Table 2). At the forefoot, average prosthetic foot axial stiffness values increased by 3.8 kN/m for every 1 stiffness category increase (*p* < 0.001) and decreased by 1.6 kN/m for every 1 cm increase in size (*p* < 0.001; Figure 5; Table 2). However, we did not detect a statistically significant effect of adding a shoe on the average forefoot prosthetic foot axial stiffness value (*p* = 0.46; Figure 5; Table 2).

**Figure 6:**
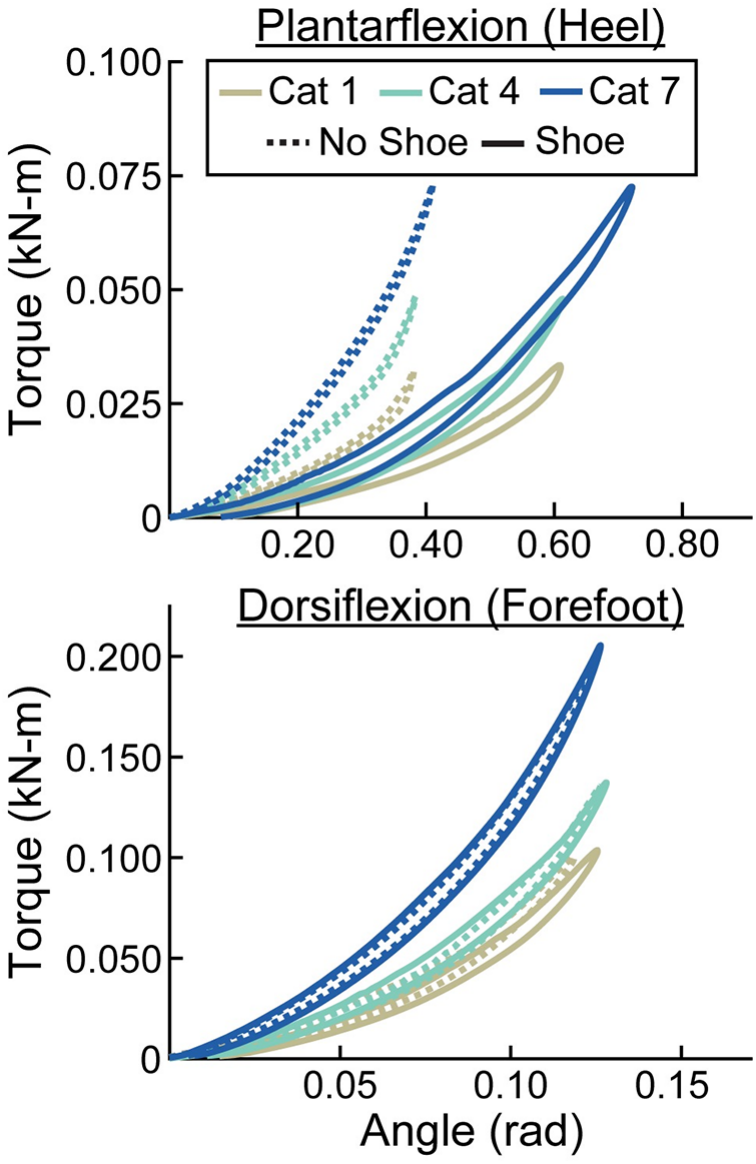
Representative torque (kN-m) vs. angle (rad) curves for plantarflexion (heel) and dorsiflexion (forefoot) of size 26 cm LP Vari-flex prosthetic feet. The colors represent different stiffness categories (categories 1, 4, 7). The dashed lines are for the tests without a shoe and the solid lines are for the tests with the shoe. The *x*- and *y*-axes differ for the plantarflexion and dorsiflexion torque and angle values.

**Figure 7:**
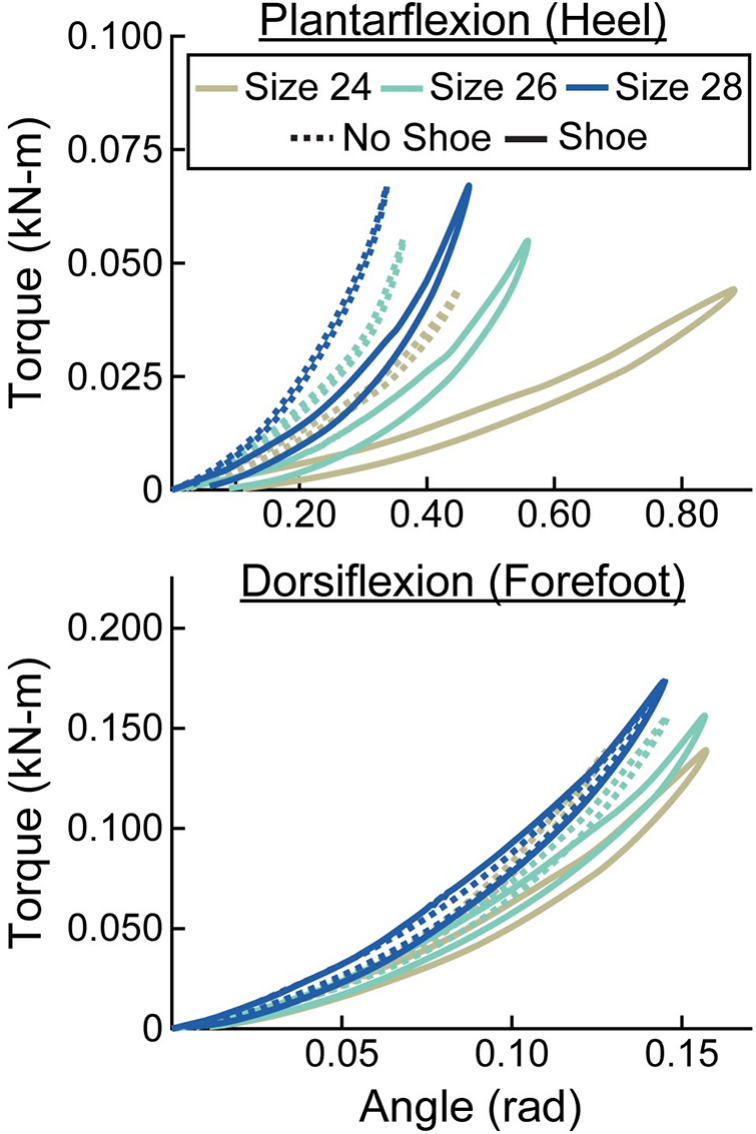
Representative torque (kN-m) vs. angle (rad) curves for plantarflexion (heel) and dorsiflexion (forefoot) of category 5 stiffness LP Vari-flex prosthetic feet. The colors represent different sizes (24 cm, 26 cm, 28 cm). The dashed lines are for the tests without a shoe and the solid lines are for the tests with the shoe. The *x*- and *y*-axes differ for the plantarflexion and dorsiflexion torque and angle values.

**Table 2:** Linear regression parameters for fixed effects of LP Vari-flex prosthetic foot stiffness category, size, and shoe or no shoe on the axial stiffness values (kN/m) at the heel, midfoot, and forefoot. Coefficient estimates, 95% confidence intervals for coefficient estimates (CI), coefficient standard errors (SE), *t* values (*t*), and *p* values (*p*) are listed for each stiffness category (1–8) and size (24–29 cm). The shoe vs. no shoe coefficient is in reference to the no shoe condition.

Heel axial stiffness (kN/m)	Estimate (B)	CI	SE	*t*	*p*
Intercept	72.85	[59.34, 86.35]	6.75	10.79	**<0.001**
Stiffness category	4.64	[4.17, 5.11]	0.23	19.77	**<0.001**
Size [cm]	−1.67	[−2.20, −1.13]	0.27	−6.22	**<0.001**
Shoe vs. no shoe	−13.51	[−15.13, −11.90]	0.81	−16.76	**<0.001**
*R*^2^ = 0.92					
Midfoot axial stiffness (kN/m)	Estimate (B)	CI	SE	*t*	*p*
Intercept	635.26	[539.35, 731.17]	47.98	13.24	**<0.001**
Stiffness category	15.63	[12.29, 18.96]	1.67	9.37	**<0.001**
Size [cm]	−19.39	[−23.21, −15.58]	1.91	−10.17	**<0.001**
Shoe vs. no shoe	−81.35	[−92.80, −69.91]	5.73	−14.21	**<0.001**
*R*^2^ = 0.85					
Forefoot axial stiffness (kN/m)	Estimate (B)	CI	SE	*t*	*p*
Intercept	66.47	[53.62, 79.33]	6.43	10.34	**<0.001**
Stiffness category	3.84	[3.39, 4.29]	0.22	17.18	**<0.001**
Size [cm]	−1.63	[−2.14, −1.12]	0.26	−6.39	**<0.001**
Shoe vs. no shoe	0.57	[−0.97, 2.10]	0.77	0.74	0.464
*R*^2^ = 0.83					

Bold values indicate significance (*p* < 0.05).

**Figure 8:**
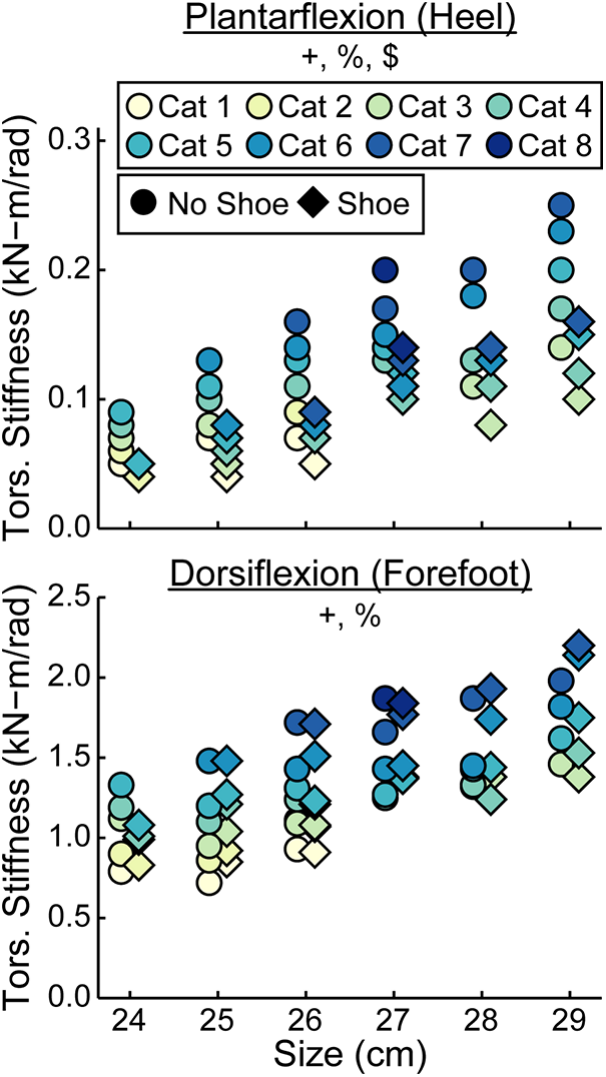
Average torsional (tors.) stiffness values (kN-m/rad) vs. LP Vari-flex prosthetic foot size in cm for plantarflexion (heel) and dorsiflexion (forefoot). The colors represent different stiffness categories (categories 1–8), the circles represent average torsional stiffness without a shoe, and the diamonds represent average torsional stiffness with a shoe. Symbols are offset for no shoe and shoe for clarity. The *y*-axis differs for the plantarflexion and dorsiflexion torsional stiffness values. + indicates a significant effect of stiffness category, % indicates a significant effect of size, and $ indicates a significant effect of shoe.

When force was applied at the heel, average prosthetic foot plantarflexion torsional stiffness values increased by 0.01 kN-m/rad for every 1 stiffness category increase (*p* < 0.001), increased by 0.02 kN-m/rad for every 1 cm increase in size (*p* < 0.001), and decreased by 0.04 kN-m/rad with the shoe compared to without the shoe (*p* < 0.001; Figures 6–8; Table 3). When force was applied at the forefoot, average prosthetic foot dorsiflexion torsional stiffness values increased by 0.12 kN-m/rad for every 1 stiffness category increase (*p* < 0.001) and increased by 0.09 kN-m/rad for every 1 cm increase in size (*p* < 0.001; Figures 6–8; Table 3). However, we did not detect a statistically significant effect of adding a shoe on the average prosthetic foot dorsiflexion torsional stiffness value (*p* = 0.31; Figure 8; Table 3).

**Table 3:** Linear regression parameters for fixed effects of LP Vari-flex prosthetic foot stiffness category, size, and shoe or no shoe on the torsional stiffness values (kN-m/rad) in plantarflexion (heel) and dorsiflexion (forefoot). Coefficient estimates, 95% confidence intervals for coefficient estimates (CI), coefficient standard errors (SE), *t* values (*t*), and *p* values (*p*) are listed for each stiffness category (1–8) and size (24–29 cm). The shoe vs. no shoe coefficient is in reference to the no shoe condition.

Plantarflexion (Heel) torsional stiffness (kN-m/rad)	Estimate (B)	CI	SE	*t*	*p*
Intercept	−0.36	[−0.42, −0.30]	0.03	−12.56	**<0.001**
Stiffness category	0.01	[0.01, 0.01]	0.00	11.85	**<0.001**
Size [cm]	0.02	[0.01, 0.02]	0.00	14.56	**<0.001**
Shoe vs. no shoe	−0.04	[−0.05, −0.03]	0.00	−11.63	**<0.001**
*R*^2^ = 0.92					
Dorsiflexion (Forefoot) torsional stiffness (kN-m/rad)	Estimate (B)	CI	SE	*t*	*p*
Intercept	−1.65	[−2.10, −1.20]	0.23	−7.30	**<0.001**
Stiffness category	0.12	[0.11, 0.14]	0.01	15.75	**<0.001**
Size [cm]	0.09	[0.07, 0.11]	0.01	10.28	**<0.001**
Shoe vs. no shoe	0.03	[−0.03, 0.08]	0.03	1.03	0.305
*R*^2^ = 0.91					

Bold values indicate significance (*p* < 0.05).

**Figure 9:**
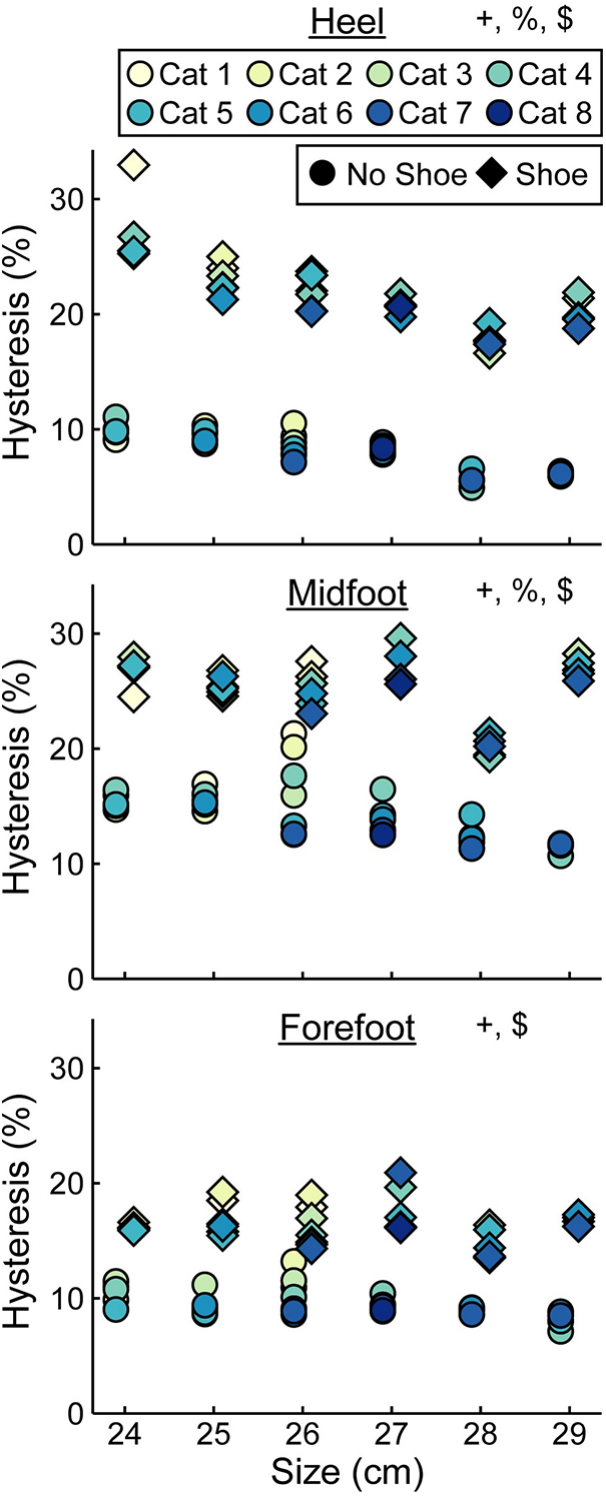
Average hysteresis (%) vs. LP Vari-flex prosthetic foot size in cm. The colors represent different prosthetic foot stiffness categories (categories 1–8), the circles represent average hysteresis without a shoe, and the diamonds represent average hysteresis with a shoe. Symbols are offset for no shoe and shoe for clarity. + indicates a significant effect of stiffness category, % indicates a significant effect of size, and $ indicates a significant effect of shoe.

Hysteresis at the heel decreased by 0.3 percentage points (p.p.) for every 1 stiffness category increase (*p* < 0.001), decreased by 1.0 p.p. for every 1 cm increase in size (*p* = 0.01), and increased by 13.8 p.p. with the shoe compared to without the shoe (*p* < 0.001; Figure 9, Table 4). Hysteresis at the midfoot decreased by 0.3 p.p. for every 1 stiffness category increase (*p* = 0.04), decreased by 0.5 p.p. for every 1 cm increase in size (*p* = 0.01), and increased by 11.0 p.p. with the shoe compared to without the shoe (*p* < 0.001; Figure 9, Table 4). Hysteresis at the forefoot decreased by 0.3 p.p. for every 1 stiffness category increase (*p* = 0.01) and increased by 7.0 p.p. with the shoe compared to without the shoe (*p* < 0.001; Figure 9, Table 4). However, we did not detect a statistically significant effect of prosthetic foot size on hysteresis at the forefoot (*p* = 0.48; Figure 9, Table 4).

**Table 4:** Linear regression parameters for fixed effects of LP Vari-flex prosthetic foot stiffness category, size, and shoe or no shoe on the hysteresis (%) at the heel, midfoot, and forefoot. Coefficient estimates, 95% confidence intervals for coefficient estimates (CI), coefficient standard errors (SE), *t* values (*t*), and *p* values (*p*) are listed for each stiffness category (1–8) and size (24–29 cm). The shoe vs. no shoe coefficient is in reference to the no shoe condition.

Heel hysteresis (%)	Estimate (B)	CI	SE	*t*	*p*
Intercept	36.56	[29.95, 43.17]	3.31	11.06	**<0.001**
Stiffness category	−0.31	[−0.54, −0.08]	0.12	−2.70	**0.009**
Size [cm]	−1.03	[−1.29, −0.76]	0.13	−7.81	**<0.001**
Shoe vs. no shoe	13.81	[13.02, 14.60]	0.39	35.00	**<0.001**
*R*^2^ = 0.96					
Midfoot hysteresis (%)	Estimate (B)	CI	SE	*t*	*p*
Intercept	28.40	[18.96, 37.84]	4.72	6.01	**<0.001**
Stiffness category	−0.34	[−0.66, −0.01]	0.16	−2.05	**0.045**
Size [cm]	−0.48	[−0.85, −0.10]	0.19	−2.54	**0.014**
Shoe vs. no shoe	10.99	[9.86, 12.12]	0.56	19.49	**<0.001**
*R*^2^ = 0.87					
Forefoot hysteresis (%)	Estimate (B)	CI	SE	*t*	*p*
Intercept	12.78	[7.22, 18.34]	2.78	4.60	**<0.001**
Stiffness category	−0.28	[−0.47, −0.08]	0.10	−2.85	**0.006**
Size [cm]	−0.08	[−0.30, 0.14]	0.11	−0.71	0.479
Shoe vs. no shoe	6.99	[6.32, 7.65]	0.33	21.05	**<0.001**
*R*^2^ = 0.88					

Bold values indicate significance (*p* < 0.05).

## 4 Discussion

In support of our hypothesis, the force-displacement curves of the LP Vari-flex prosthetic foot at the heel, midfoot, and forefoot (Supplementary Material: Force-Displacement Equations) and the plantarflexion and dorsiflexion torque-angle curves (Supplementary Material: Torque-Angle Equations) exhibited a curvilinear profile and were well-described by a quadratic curve (average adjusted *R*^2^ for all tests: 1.00). These results are similar to the curvilinear stiffness exhibited by the higher profile prosthetic model, the Vari-flex (11), likely because both prostheses have a similar design and are made of carbon fiber. The LP Vari-flex prosthetic foot force-displacement and torque-angle curves have a steeper slope with greater applied forces and torques and thus stiffen with displacement. This suggests that the stiffness of the prosthetic foot differs during dynamic tasks where forces change and thus prosthetists may need to consider the activity when prescribing a given prosthetic foot stiffness category. For example, based on the force-displacement equations (Supplementary Table S4), we estimate that when a 70 kg person uses a category 4, size 27 prosthesis inside of a walking shoe, the axial stiffness at the heel is 35.1 kN/m for a load consistent with walking at 0.75 m/s [1.0 BW first peak vertical ground reaction force (30)], but this value increases to 40.5 kN/m for a load consistent with walking at 1.75 m/s [1.3 BW first peak vertical ground reaction force (30)]. The change in axial stiffness at the heel between low and high loading (5.4 kN/m) is similar to the change in average axial stiffness at the heel for a 1 category increase (4.6 kN/m; Table 2), suggesting that it is meaningful to consider the curvilinear nature of LP Vari-flex prosthetic feet.

We found that a greater numerical stiffness category of the LP Vari-flex prosthetic foot resulted in increased average axial stiffness values at the heel, midfoot, and forefoot by 4.6, 15.6, and 3.8 kN/m per one category increase, respectively. The change in heel stiffness values between categories of the LP Vari-flex prosthetic foot were similar to the changes in the higher profile prosthetic foot model (Vari-flex), where heel axial stiffness values increase by about 6–7 kN/m per one category increase (11, 19). In general, we found that the heel and forefoot axial stiffness values of the LP Vari-flex prosthetic foot for a given stiffness category are stiffer than the higher profile Vari-flex prosthetic foot model. For example, the average axial stiffness value of the heel for a size 27 LP Vari-flex without a shoe ranges from 49.1 to 58.7 kN/m for categories 5–7, whereas the average axial stiffness value of the heel for a size 27 Vari-flex prosthetic foot without a shoe ranges from 37.5 to 45.4 kN/m in Turner et al. (20) and 36.4 to 47.1 kN/m in Ruxin et al. (11) for categories 5–7. Moreover, the average axial stiffness value of the forefoot for a size 27 LP Vari-flex without a shoe ranges from 36.2 to 47.0 kN/m for categories 5–7, whereas the average axial stiffness value of the forefoot for a size 27 Vari-flex prosthetic foot without shoes for categories 5–7 ranges from 29.1 to 38.5 kN/m in Turner et al. (20) and 28.6 to 40.0 kN/m in Ruxin et al. (11). These differences in stiffness values between the same category of the LP Vari-flex and Vari-flex may be clinically meaningful. The average forefoot stiffness of the size 27 category 5 LP Vari-flex prosthesis is 7.6 kN/m or 26.6% greater than the Vari-flex prosthesis (11). A previous study suggested that a 10% change in prosthetic foot stiffness is the minimum clinically important difference (22). Furthermore, a previous study of people with amputation walking with an experimental prosthesis at 0.7–1.5 m/s suggests that a 7.6 kN/m increase in forefoot stiffness can increase the magnitude of unaffected leg negative center-of-mass work by 0.9 J on average (1), which may increase the risk of osteoarthritis in the unaffected leg knee (35). Ultimately, the differences in heel and forefoot axial stiffness values between the LP Vari-flex and Vari-flex prosthetic feet suggest that prosthetists should prescribe a lower stiffness category for the LP Vari-flex prosthesis than they would for the Vari-flex prosthesis. Furthermore, the changes in axial stiffness values of the LP Vari-flex prosthetic foot without a shoe between prosthetic stiffness categories for the heel, midfoot, and forefoot were variable (Figure 5). Previous studies that characterized commercially available passive-elastic prosthetic feet found similar results (11, 19). The variable changes in axial stiffness values between stiffness categories highlight the need for objective measurements of prosthetic foot stiffness values within and between manufacturers because our results show that an increase in the stiffness category may not always result in an actual increase in the stiffness of the prosthesis. Such measurements would improve the understanding of the mechanical function provided by prostheses.

We also found that a greater stiffness category of the LP Vari-flex prosthetic foot resulted in an increased average torsional stiffness value for plantarflexion (heel) and dorsiflexion (forefoot) by 0.01 and 0.12 kN-m/rad, respectively. Major et al. estimated the torsional stiffness values of three commercially-available prosthetic feet, the SACH foot, Seattle foot, and Flex-foot, and found that the plantarflexion (heel) and dorsiflexion (forefoot) stiffness values ranged from 0.09 to 0.20 kN-m/rad and 0.39 to 1.40 kN-m/rad, respectively (6). Similarly, we found that plantarflexion (heel) stiffness values of the LP Vari-flex foot without a shoe ranged from 0.05 to 0.25 kN-m/rad and dorsiflexion (forefoot) stiffness values of the LP Vari-flex foot without a shoe ranged from 0.72 to 1.98 kN-m/rad across the tested stiffness categories and sizes. Different torsional stiffness values of prosthetic feet can affect the joint angles, peak ground reaction forces, and metabolic cost of people with unilateral transtibial amputation during walking (6), so characterizing the torsional stiffness values of prosthetic feet can be a useful tool for predicting how different prosthetic feet will affect walking biomechanics. Moreover, since a biological ankle can behave mechanically like a torsional spring and damper system during walking at 1.2 m/s (21), torsional stiffness values of prosthetic feet provide information that can be compared to the biological ankle-foot system (36) to derive function and potentially inform biomimetic prosthetic prescription and design.

In contrast to our prediction, we found that a greater LP Vari-flex prosthetic foot stiffness category resulted in a 0.3 percentage point decrease in hysteresis for the heel, midfoot, and forefoot, which is a relatively small effect. The hysteresis at the heel, midfoot, and forefoot of the LP Vari-flex prosthetic feet without a shoe averaged across sizes ranged from 6.9% to 10.3%, 12.1% to 18.1%, and 8.8% to 10.7%, respectively. Therefore, it is unclear if the effect of LP Vari-flex prosthetic foot stiffness category on hysteresis is clinically meaningful. Future studies should examine the independent effects of prosthetic hysteresis on kinematics, kinetics, muscle activity, metabolic cost and user preference during walking to determine the clinically meaningful difference in prosthetic hysteresis. Nonetheless, prosthetists may want to consider that LP Vari-flex prosthetic feet with stiffer categories have less hysteresis than less stiff categories when prescribing prosthetic feet.

We partially reject our hypothesis that an increase in prosthetic foot size would have no effect on axial stiffness values or hysteresis but would increase torsional stiffness values. We found that a 1 cm increase in the size of the LP Vari-flex prosthetic foot resulted in a 1.7, 19.4, and 1.6 kN/m decrease in axial stiffness values at the heel, midfoot, and forefoot, respectively, an 0.02 and 0.09 kN-m/rad increase in plantarflexion (heel) and dorsiflexion (forefoot) torsional stiffness values, respectively, and a 1.0 and 0.5 percentage point decrease in the hysteresis at the heel and midfoot, respectively. As hypothesized, an increase in prosthetic foot size resulted in an increase in torsional stiffness values due to an increase in the moment arm of the prosthesis. Despite the fact that manufacturers recommend the same LP Vari-flex prosthetic foot stiffness category for a given body mass and activity level regardless of prosthetic foot size (10), prosthetic foot size does affect axial stiffness values, torsional stiffness values, and hysteresis. Since axial stiffness values, torsional stiffness values, and hysteresis can affect kinematics, kinetics, muscle activity, metabolic cost and user preference during walking (1–7), prosthetists should consider that an increase in the size of the LP Vari-flex prosthetic foot can decrease axial stiffness values, decrease hysteresis, and increase torsional stiffness values when prescribing prosthetic feet. Furthermore, manufacturers should design prosthetic feet to have similar mechanical properties for a given prosthetic foot stiffness category regardless of the prosthetic foot size.

In support of our hypothesis, we found that adding a shoe to the LP Vari-flex prosthetic foot decreased axial stiffness values at the heel and midfoot by 13.5 and 81.4 kN/m, respectively, and increased hysteresis at the heel, midfoot, and forefoot by 13.8, 11.0, and 7.0 percentage points, respectively. Our results are similar to those of Major et al. who found that adding an athletic shoe to the prosthetic foot decreased heel and midfoot axial stiffness values by 20.5 kN/m and 151.6 kN/m, respectively, and increased hysteresis at the heel, midfoot, and forefoot by 7.4, 9.3, and 3.4 percentage points, respectively, compared to values for a prosthetic foot without a shoe (27). Moreover, similar to Major et al., we found that adding a shoe did not affect forefoot axial stiffness values (27). Ultimately, adding a shoe to a prosthetic foot affects the heel and midfoot axial stiffness values and heel, midfoot, and forefoot hysteresis, so footwear should be considered when determining how different prosthetic feet affect kinematics, kinetics, muscle activity, metabolic cost, and user preference of people with transtibial or transfemoral amputation.

In contrast to our hypothesis that adding a shoe to the LP Vari-flex prosthetic foot would not affect torsional stiffness values, we found that adding a shoe resulted in a decrease of plantarflexion (heel) torsional stiffness by 0.04 kN-m/rad but did not affect dorsiflexion (forefoot) torsional stiffness. Overall, adding a shoe to the LP Vari-flex prosthetic foot affects heel and midfoot axial stiffness values, heel, midfoot, and forefoot hysteresis, and plantarflexion (heel) torsional stiffness values. Previous studies have found that different types of footwear can have different effects on stiffness and hysteresis (27). This highlights the need to consider footwear when choosing and aligning prosthetic feet and predicting how different prosthetic feet may affect kinematics, kinetics, muscle activity, metabolic cost, and user preference of people with transtibial or transfemoral amputation during walking.

Our study had some potential limitations. We used a uniaxial load cell (Instron 2580-201, Norwood, MA), so we were unable to measure off-axis forces on the load cell during the heel and forefoot tests. We used a low-friction roller system to reduce off-axis forces on the load cell and derived equations (1)–(3) to estimate the actual force applied to the prosthetic foot based on the force measured by the uniaxial load cell (Supplementary Material: Derivation and Verification of Equations 1–3). However, since the low-friction roller system is not perfectly frictionless, we may have overestimated the force on the prosthetic foot (Supplementary Material: Derivation and Verification of Equations 1–3). We conducted a *post hoc* analysis of the forefoot test with one prosthetic foot using a multi-axis force transducer (MC3A-500, AMTI, Watertown, MA, USA) and found that our estimate of the force applied to the prosthetic foot from equation (1) overestimated the actual force measured by the multi-axis force transducer by 1% (Supplementary Material: Derivation and Verification of Equations 1–3). Another potential limitation is that we estimated the torque and angle of each prosthetic foot assuming a constant moment arm (r) from the point of contact of the foot to the pylon when the prosthesis was preloaded to 4–6 N for the heel and forefoot tests. However, the moment arm may have decreased as the prosthetic foot was plantarflexed during the heel test and dorsiflexed during the forefoot test despite the low friction roller system. Therefore, we may have overestimated the torque on the prosthetic foot.

For our study, we only tested the effects of one type of walking shoe, which does not represent all types of footwear that people with amputation wear during daily life. Major et al. characterized the stiffness and hysteresis of different prosthetic feet inside several different types of footwear that included a hiking boot, athletic shoe, leather dress shoe, and flat shoe (27). The shoe that we tested is similar to the athletic shoe described in Major et al. (27). Major et al. found that of all the tested shoes, the athletic shoe resulted in the greatest change in stiffness and hysteresis relative to the condition without a shoe (27). Therefore, we expect that the differences in stiffness and hysteresis between prostheses with and without a shoe that we found in our study are likely greater than if we had tested a hiking boot, leather dress shoe, or flat shoe. Future studies should measure the effects of different types of footwear on the mechanical properties of LP Vari-flex feet or measure the prosthetic ankle torque-angle curves during walking with different footwear to provide characterization of the mechanical properties of prosthetic feet.

In addition to the mechanical properties of passive-elastic prosthetic feet, the alignment of the prosthesis relative to the socket can affect the function of the prosthesis during walking and is important for prosthetists to consider when prescribing prosthetic feet (37). When prescribing prosthetic feet, prosthetists often adjust the alignment of the prosthesis depending on if the person with amputation feels the prosthesis is too compliant or too stiff. Objective stiffness values of prosthetic devices can be used by prosthetists when choosing the prosthetic device, but the prosthesis can be further adjusted by changing its alignment. Future studies should examine how different alignments of the prosthesis relative to the socket can affect the mechanical properties of LP Vari-flex feet and provide guidelines for aligning prosthetic feet.

In conclusion, we characterized the axial stiffness values, torsional stiffness values, and hysteresis of LP Vari-flex prosthetic feet with a range of stiffness categories and sizes without and with shoes. In general, a greater prosthetic foot stiffness category resulted in an increase in heel, midfoot, and forefoot axial stiffness values, an increase in plantarflexion and dorsiflexion torsional stiffness values, and a decrease in heel, midfoot, and forefoot hysteresis. Moreover, an increase in prosthetic foot size resulted in a decrease in heel, midfoot, and forefoot axial stiffness values, an increase in plantarflexion and dorsiflexion torsional stiffness values, and a decrease in heel and midfoot hysteresis. Finally, adding a shoe to the LP Vari-flex prosthetic foot resulted in a decrease in heel and midfoot axial stiffness values, a decrease in plantarflexion torsional stiffness values, and an increase in heel, midfoot, and forefoot hysteresis. Thus, estimating the dynamic function of prosthetic feet without and with a shoe may be affected by the manufacturer-recommended prosthetic foot stiffness category and size as well as the footwear used in combination with the prosthesis. Future research and/or manufacturers should characterize the mechanical properties of prosthetic feet and footwear prior to experimental testing or prescription to better understand the resulting effects of mechanical properties on the user's walking biomechanics, preferences, daily activities, and the usability and acceptability of the prosthesis.

Overall, the axial and torsional stiffness values, hysteresis, and force-displacement equations of LP Vari-flex prosthetic feet with and without a shoe can be used to objectively compare LP Vari-flex prosthetic feet to other prosthetic feet to inform their prescription and design and use by people with a transtibial or transfemoral amputation. Prosthetists can compare our objective stiffness values to values reported for other prosthetic feet (11) rather than using manufacturer-defined categories that can be inconsistent or subjective. For example, the recommended stiffness category of the Vari-flex prosthesis for a 70 kg person with a moderate impact level is a category 4, which has an average stiffness of 35.7 kN/m at the heel and 26.7 kN/m at the forefoot for the size 27 prosthesis (20). If a prosthetist wants to prescribe an LP Vari-flex prosthesis of the same size with similar characteristics as the Vari-flex prosthesis, they could use the equations from Table 2 (average stiffness = intercept + *B*_1_ × stiffness category + *B*_2_ × size + *B*_3_ × shoe/no shoe) to determine which category LP Vari-flex prosthesis has the same heel and forefoot stiffness as the Vari-flex prosthesis. Our results suggest they should prescribe the category 2 prosthesis, which has an average stiffness of 37.0 kN/m at the heel (from Table 2: average heel stiffness = 72.85 + (4.64 × 2) + (−1.67 × 27) + (−13.51 × 0)) and 30.14 kN/m at the forefoot (from Table 2: average forefoot stiffness = 66.47 + (3.84 × 2) + (−1.63 × 27) + (0.57 × 0)). Similarly, since prosthetists typically choose the prosthetic foot size to match the size of the biological foot, a prosthetist can use our results in Tables 2–4 to ensure consistent characteristics for people of similar weight and impact level for a range of different foot sizes. Future work should synthesize our results and previous studies to create tables with objective stiffness values for different prostheses so prosthetists can compare different prosthetic foot categories, sizes, and models. In addition, our results can be used by researchers conducting studies on the effects of prosthetic feet with different mechanical properties on walking biomechanics, such as kinematics, kinetics, muscle activity, and metabolic cost. Moreover, researchers can use the force-displacement and torque-angle equations to design experimental prosthetic feet with mechanical properties that match commercially available prosthetic feet.

## Data Availability

The original contributions presented in the study are included in the article/**Supplementary Material**, further inquiries can be directed to the corresponding author.

## Supplementary material

DATA SHEET 1

Code for statistics and figures.

DATA SHEET 2

Average axial and torsional stiffness and hysteresis data.

DATA SHEET 3

Derivation and verification of Equations 1–3.

DATA SHEET 4

Information about Data.

DATA SHEET 5

Force-displacement equations during the loading phase for the heel, midfoot, and forefoot tests with and without a shoe.

DATA SHEET 6

Torque-angle equations during the loading phase for the heel and forefoot tests with and without a shoe.

DATA SHEET 7

Force-displacement equations during the unloading phase for the heel, midfoot, and forefoot tests with and without a shoe.

DATA SHEET 8

Torque-angle equations during the unloading phase for the heel and forefoot tests with and without a shoe.
