# Supplementary material for: Low-profile prosthetic foot stiffness category and size, and shoes affect axial and torsional stiffness and hysteresis
Source: Front Rehabil Sci. 2024 Feb 28;5:1290092. doi: 10.3389/fresc.2024.1290092 (PMC10932964; doi:10.3389/fresc.2024.1290092)
Supplement: Supplementary file 1 [file Datasheet1.pdf]

Code for Statistics and Plots for 'Low-profile prosthetic foot stiffness category and size, and shoes affect axial and torsional stiffness and hysteresis'

Joshua R. Tacca, Zane A. Colvin, and Alena M. Grabowski

01/03/2024

Load Data

```
#all data
stiff_data <- read.csv("Data.csv")
```

Prepare Data

```
width <- 0.1

#Create a column where the sizes are offset based on the shoe, to make the graphs clearer
stiff_data$Size_shift <- stiff_data$Size + width*ifelse(stiff_data$No_Shoe.Shoe == "Shoe", 1, -1)

#Create a column where the stiffness categories are offset based on the shoe to make the graphs clearer
stiff_data$Stiff_num <- ifelse(stiff_data$Stiff_cat == "cat1", 1,
                                 ifelse(stiff_data$Stiff_cat == "cat2", 2,
                                        ifelse(stiff_data$Stiff_cat == "cat3", 3,
                                               ifelse(stiff_data$Stiff_cat == "cat4", 4,
                                                      ifelse(stiff_data$Stiff_cat == "cat5", 5,
                                                             ifelse(stiff_data$Stiff_cat == "cat6", 6,
                                                                    ifelse(stiff_data$Stiff_cat == "cat

stiff_data$Stiff_shift <- stiff_data$Stiff_num + width*ifelse(stiff_data$No_Shoe.Shoe == "Shoe", 1, -1)

midfoot_data <- stiff_data[stiff_data$Test == "midfoot",]
heel_data <- stiff_data[stiff_data$Test == "heel",]
forefoot_data <- stiff_data[stiff_data$Test == "forefoot",]
```

Plots

Figure 5: Heel Test; Average Stiffness during Loading vs. Size

```
#Heel, stiff vs. size, group by category and shoe
heel_kavg <- ggplot(data = heel_data, aes(x=Size_shift, y=Kavg_load, shape = No_Shoe.Shoe, fill = Stiff
  scale_shape_manual(values = c(21, 23))+
  geom_point(size = 4, alpha = 1)+
  labs(x = "Size (cm)", y = "Stiffness (kN/m)")+
  scale_fill_brewer(palette = "YlGnBu")+
  scale_color_brewer(palette = "YlGnBu")+
```

```r
  scale_y_continuous(limits = c(0,80), expand = c(0,0))+
  theme_classic()

heel_kavg
```

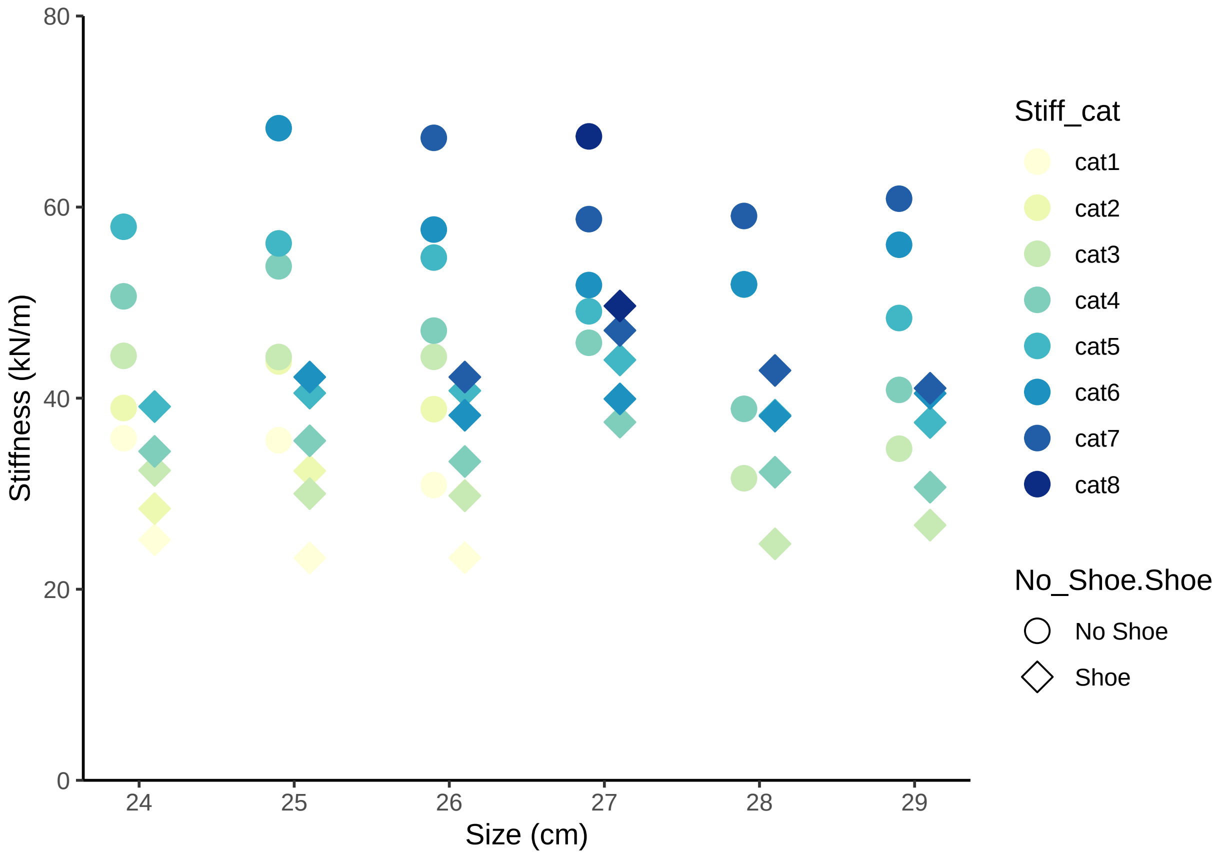

Figure 5: Midfoot Test; Average Stiffness during Loading vs. Size

```r
#Midfoot, stiff vs. size, group by category and shoe
mdf_kavg <- ggplot(data = midfoot_data, aes(x=Size_shift, y=Kavg_load, shape = No_Shoe.Shoe, fill = Sti
  scale_shape_manual(values = c(21, 23))+
  geom_point(size = 4, alpha = 1)+
  labs(x = "Size (cm)", y = "Stiffness (kN/m)")+
  scale_fill_brewer(palette = "YlGnBu")+
  scale_color_brewer(palette = "YlGnBu")+
  scale_y_continuous(limits = c(0,300), expand = c(0,0))+
  theme_classic()

mdf_kavg
```

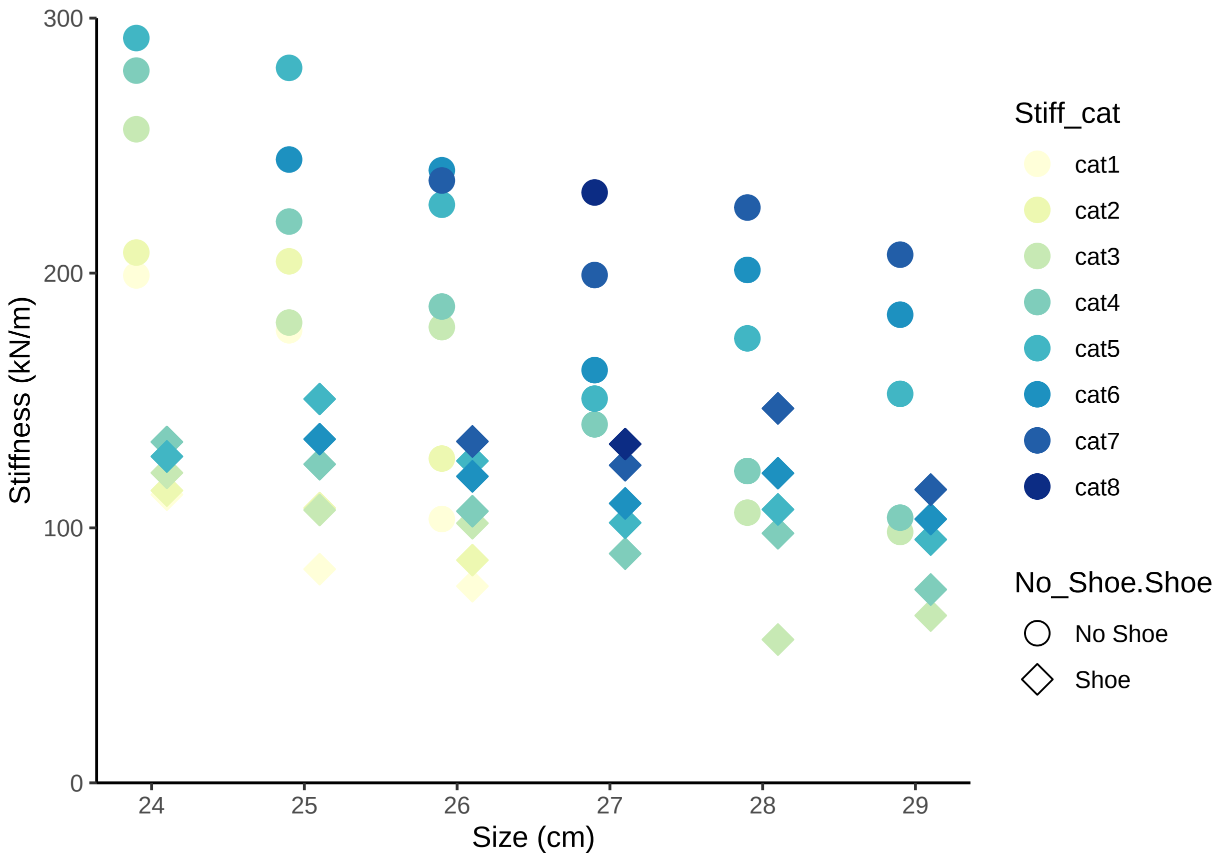

Figure 5: Forefoot Test; Average Stiffness during Loading vs. Size

```
#Forefoot, stiff vs. size, group by category and shoe
frf_kavg <- ggplot(data = forefoot_data, aes(x=Size_shift, y=Kavg_load, shape = No_Shoe.Shoe, fill = St
  scale_shape_manual(values = c(21, 23))+
  geom_point(size = 4, alpha = 1)+
  labs(x = "Size (cm)", y = "Stiffness (kN/m)")+
  scale_fill_brewer(palette = "YlGnBu")+
  scale_color_brewer(palette = "YlGnBu")+
  scale_y_continuous(limits = c(0,80), expand = c(0,0))+
  theme_classic()

frf_kavg
```

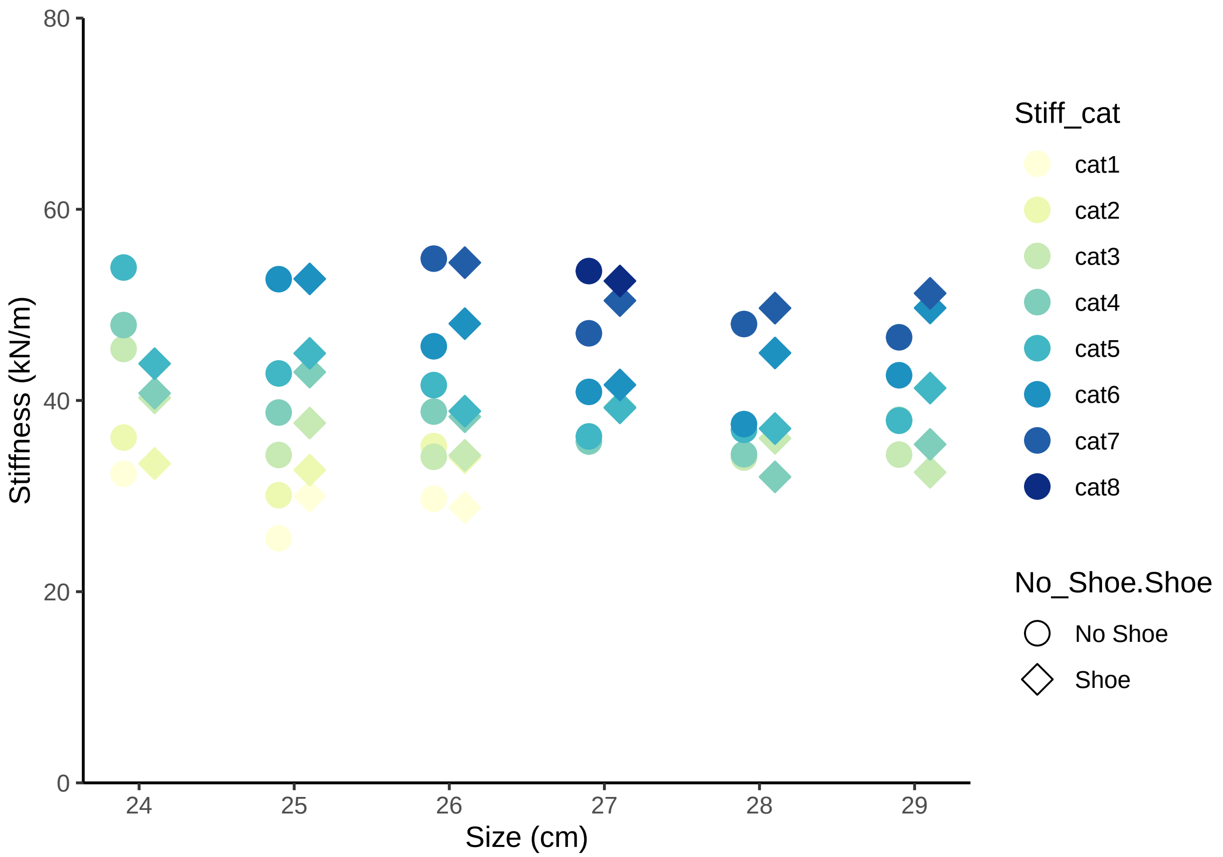

Export Figure 5

```
#combine heel, midfoot, and  forefoot for Figure 5
fig5 <- plot_grid(heel_kavg+theme(legend.position = "none"), mdf_kavg+theme(legend.position = "none"), f
fig5
```

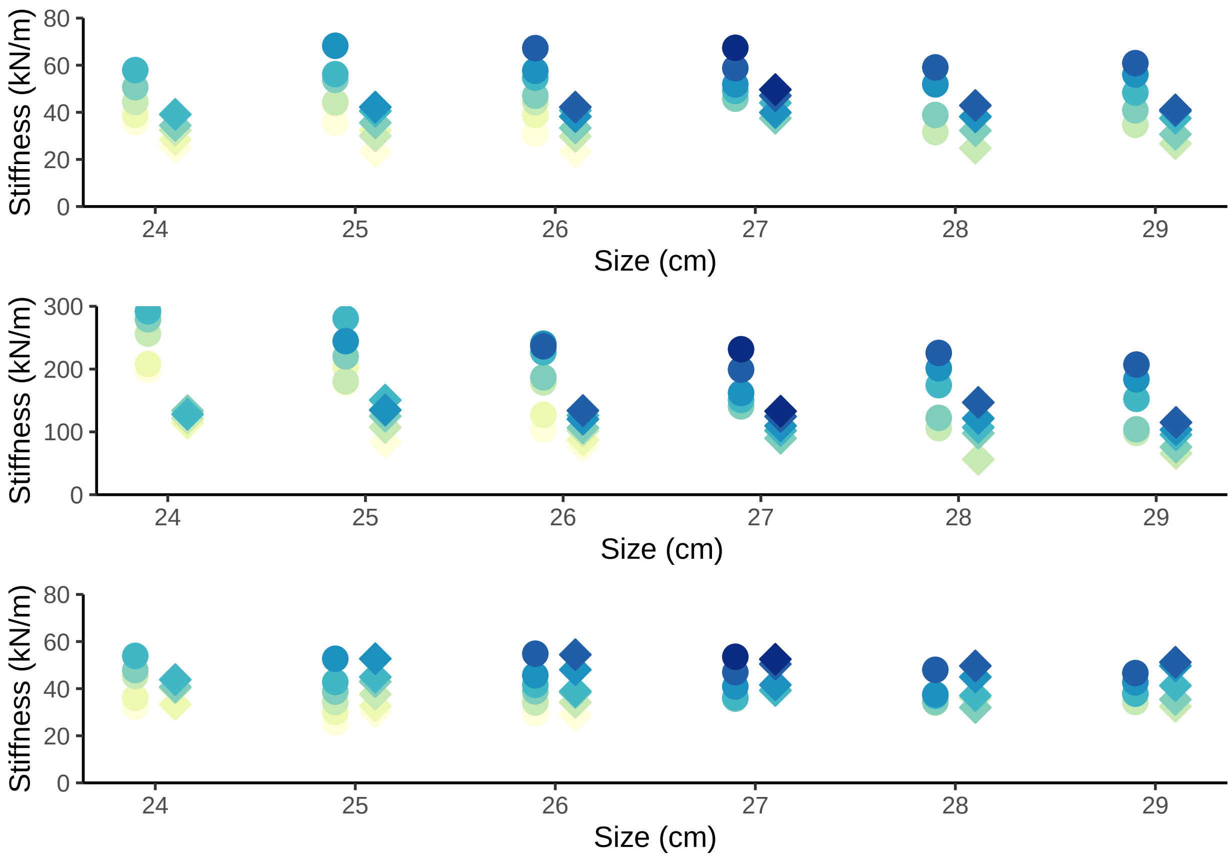


```
ggsave("fig5_v1.pdf", plot = fig5, device = "pdf", useDingbats = FALSE, width = 3.25, height = 8, units
```

Figure 8: Torsional Stiffness vs. Size

```
#Heel (Plantarflexion), stiff vs. size, group by category and shoe
heel_tors <- ggplot(data = heel_data, aes(x=Size_shift, y=Ktors_avg_load, shape = No_Shoe.Shoe, fill = S
  scale_shape_manual(values = c(21, 23))+
  geom_point(size = 4, alpha = 1)+
  labs(x = "Size (cm)", y = "Angular Stiffness (kN-m/rad)")+
  scale_fill_brewer(palette = "YlGnBu")+
  scale_color_brewer(palette = "YlGnBu")+
  scale_y_continuous(limits = c(0,0.31), expand = c(0,0))+
  theme_classic()

heel_tors
```

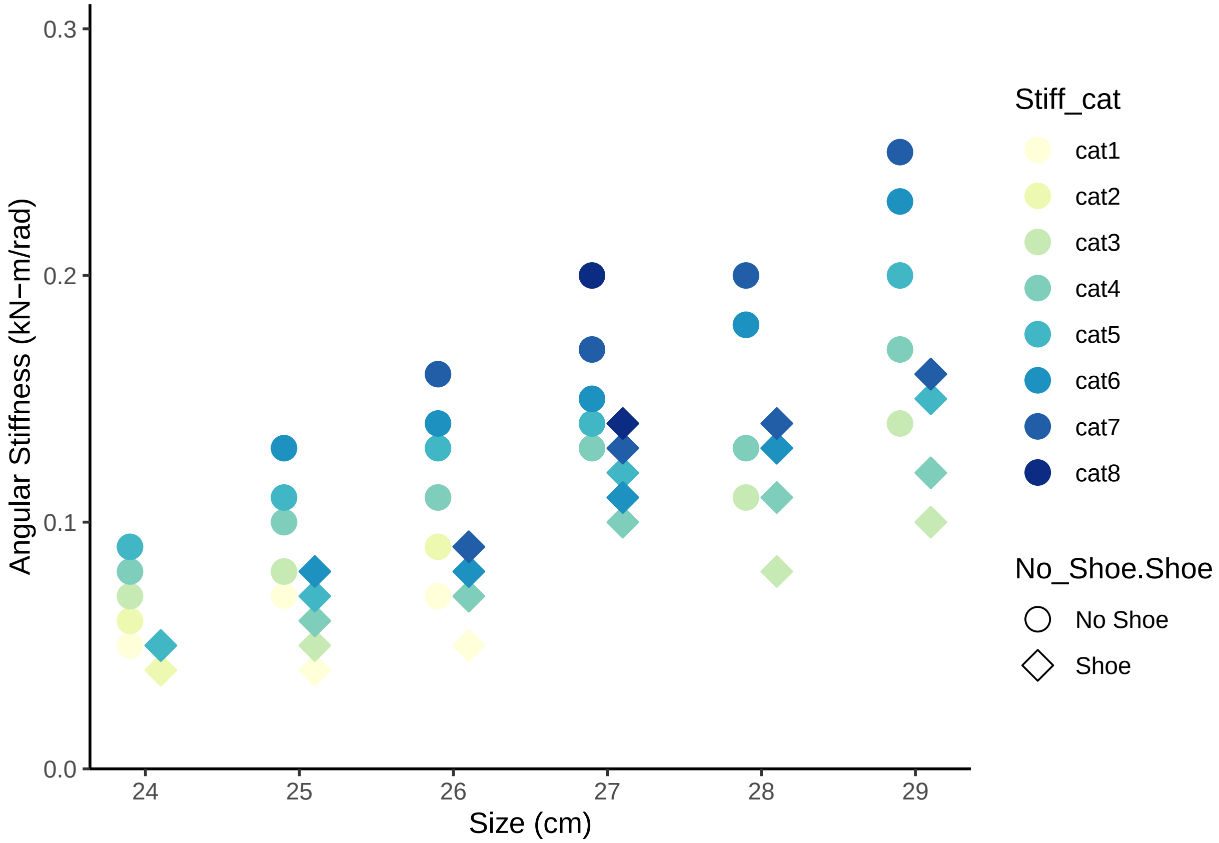


```
#Forefoot (Dorsiflexion), stiff vs. size, group by category and shoe
fore_tors <- ggplot(data = forefoot_data, aes(x=Size_shift, y=Ktors_avg_load, shape = No_Shoe.Shoe, fil
  scale_shape_manual(values = c(21, 23))+
  geom_point(size = 4, alpha = 1)+
  labs(x = "Size (cm)", y = "Angular Stiffness (kN-m/rad)")+
  scale_fill_brewer(palette = "YlGnBu")+
  scale_color_brewer(palette = "YlGnBu")+
  scale_y_continuous(limits = c(0,2.5), expand = c(0,0))+
  theme_classic()

fore_tors
```

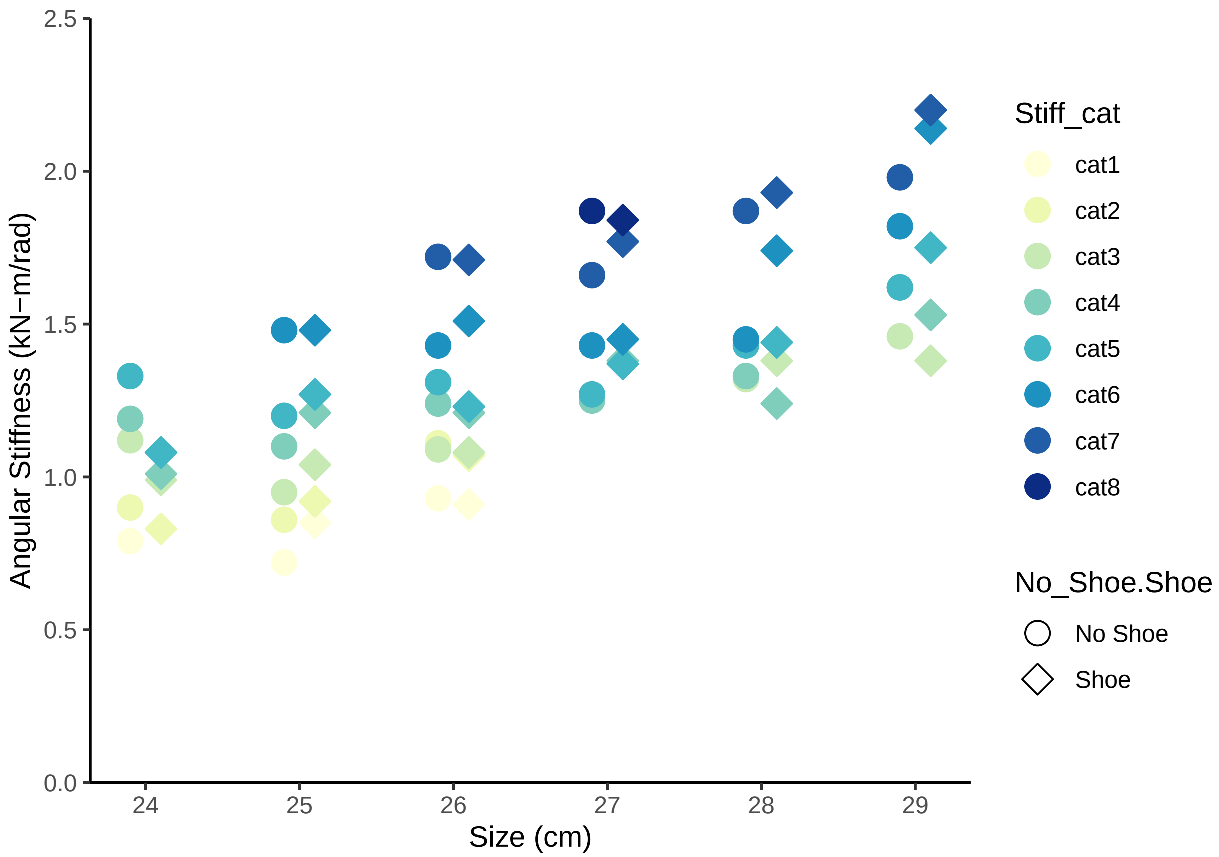

Export Figure 8

```
#combine heel, midfoot, and forefoot
fig8 <- plot_grid(heel_tors+theme(legend.position = "none"), fore_tors+theme(legend.position = "none"),
fig8
```

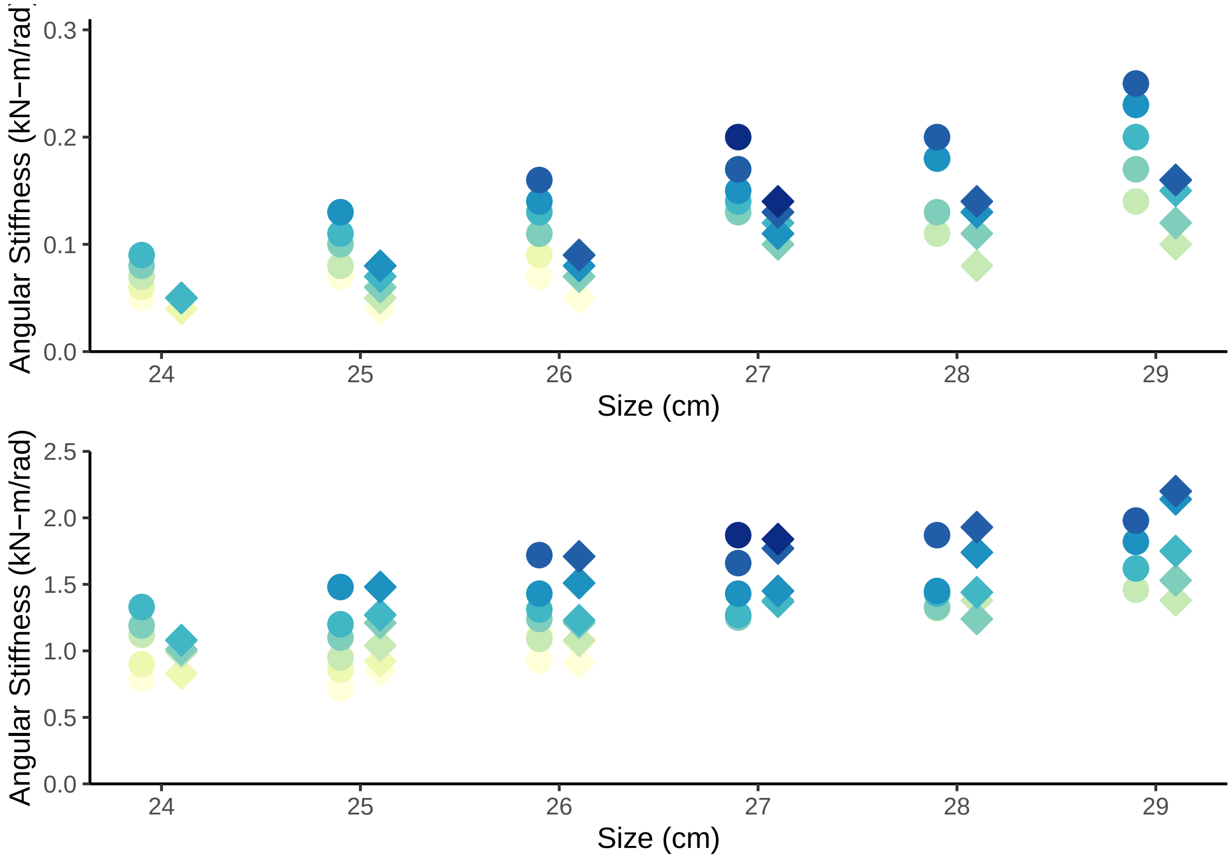


```
ggsave("fig8.pdf", plot = fig8, device = "pdf", useDingbats = FALSE, width = 3.25, height = 5.3, units =
```

Figure 9: Hysteresis Plots

```
#Heel, hysteresis vs. size, group by category and shoe
heel_hys <- ggplot(data = heel_data, aes(x=Size_shift, y=Per_hysteresis, shape = No_Shoe.Shoe, fill = St
  scale_shape_manual(values = c(21, 23))+
  geom_point(size = 4, alpha = 1)+
  labs(x = "Size (cm)", y = "Hysteresis (%)")+
  scale_fill_brewer(palette = "YlGnBu")+
  scale_color_brewer(palette = "YlGnBu")+
  scale_y_continuous(limits = c(0,35), expand = c(0,0))+
  theme_classic()

heel_hys
```

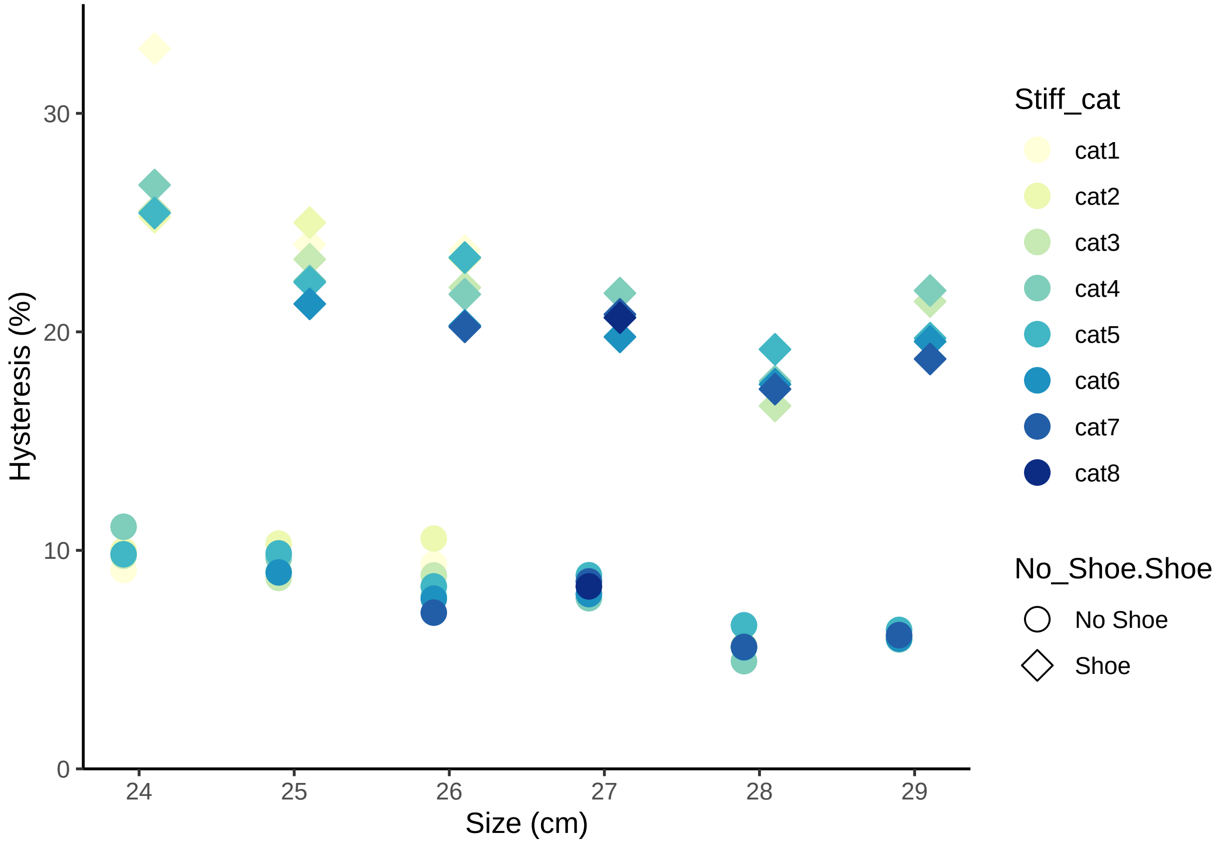


```r
#Midfoot, hysteresis vs. size, group by category and shoe
mdf_hys <- ggplot(data = midfoot_data, aes(x=Size_shift, y=Per_hysteresis, shape = No_Shoe.Shoe, fill =
  scale_shape_manual(values = c(21, 23))+
  geom_point(size = 4, alpha = 1)+
  labs(x = "Size (cm)", y = "Hysteresis (%)")+
  scale_fill_brewer(palette = "YlGnBu")+
  scale_color_brewer(palette = "YlGnBu")+
  scale_y_continuous(limits = c(0,35), expand = c(0,0))+
  theme_classic()

mdf_hys
```

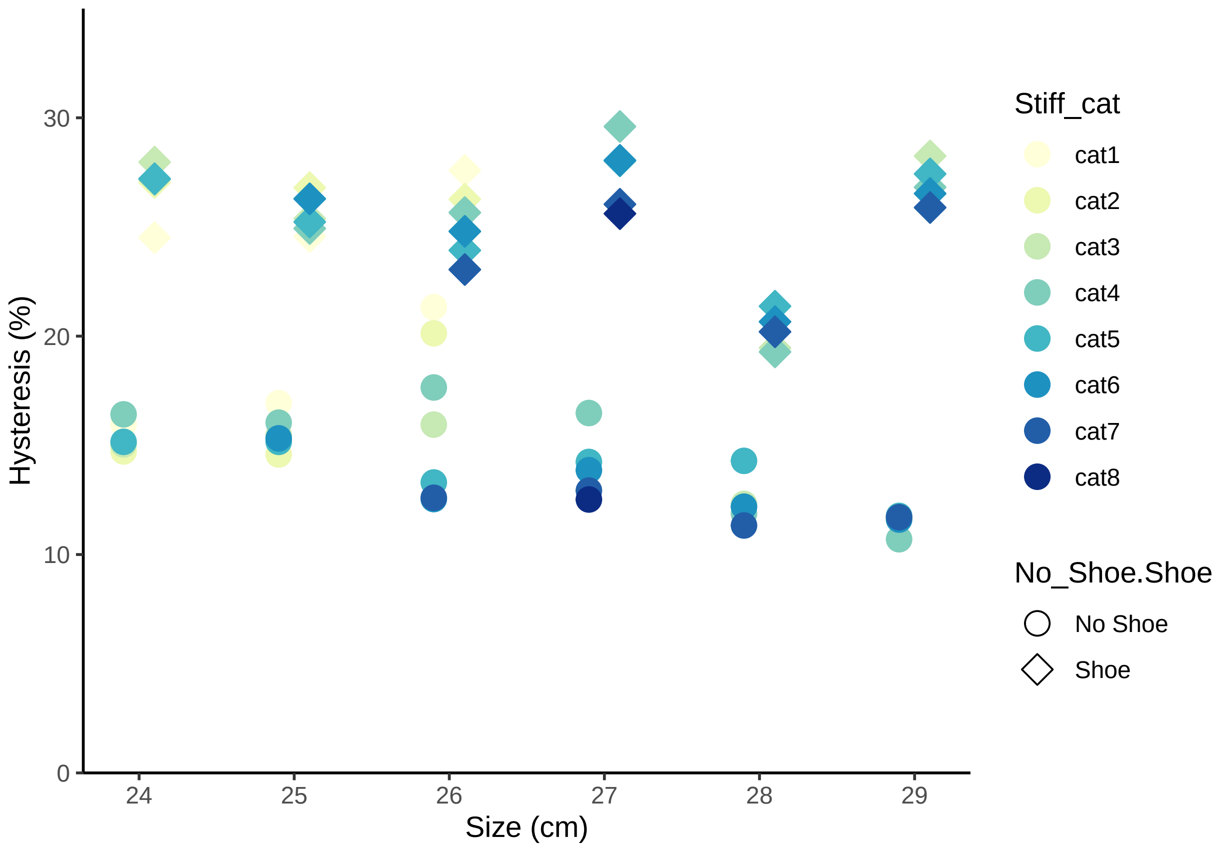


```
#Forefoot, hysteresis vs. size, group by category and shoe
for_hys <- ggplot(data = forefoot_data, aes(x=Size_shift, y=Per_hysteresis, shape = No_Shoe.Shoe, fill =
  scale_shape_manual(values = c(21, 23))+
  geom_point(size = 4, alpha = 1)+
  labs(x = "Size (cm)", y = "Hysteresis (%)")+
  scale_fill_brewer(palette = "YlGnBu")+
  scale_color_brewer(palette = "YlGnBu")+
  scale_y_continuous(limits = c(0,35), expand = c(0,0))+
  theme_classic()

for_hys
```

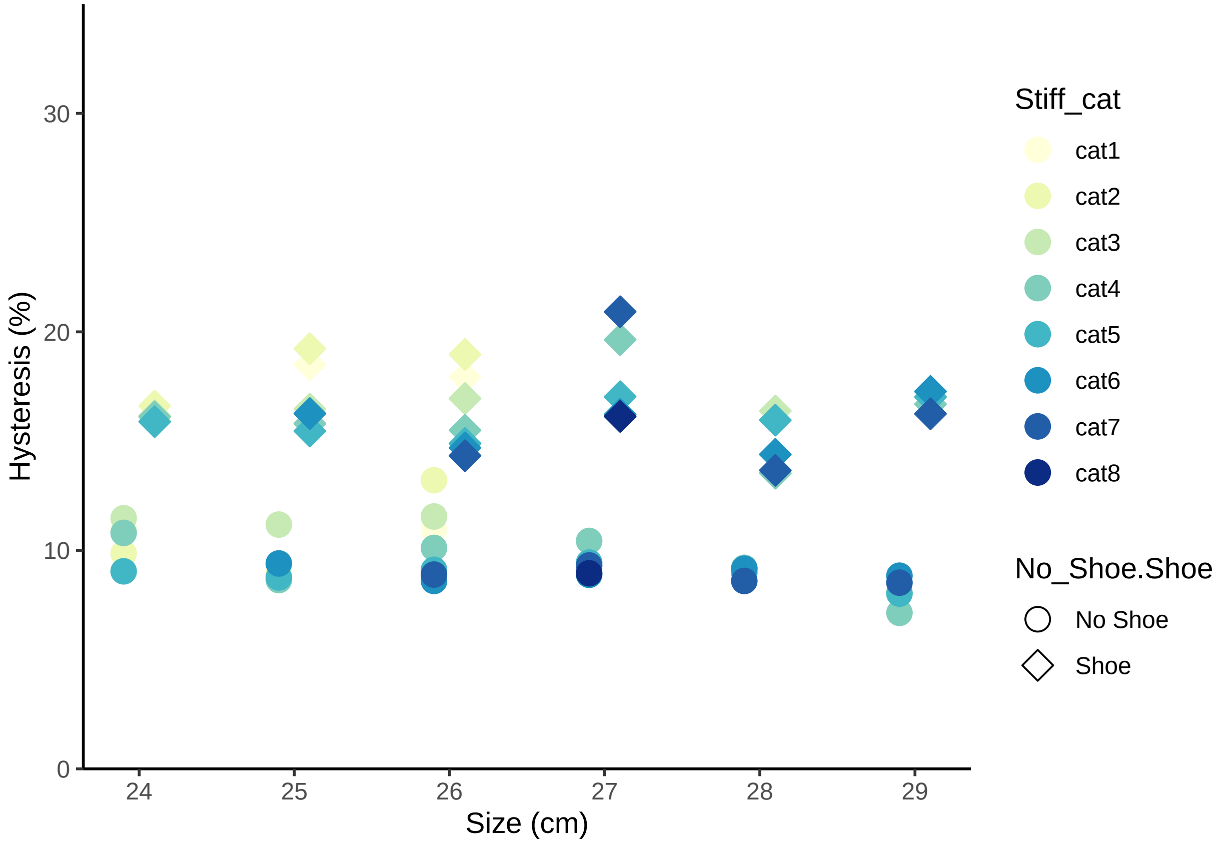

Export Figure 9

```
#combine heel, midfoot, and forefoot for Figure 9
fig9 <- plot_grid(heel_hys+theme(legend.position = "none"), mdf_hys+theme(legend.position = "none"), fo
fig9
```

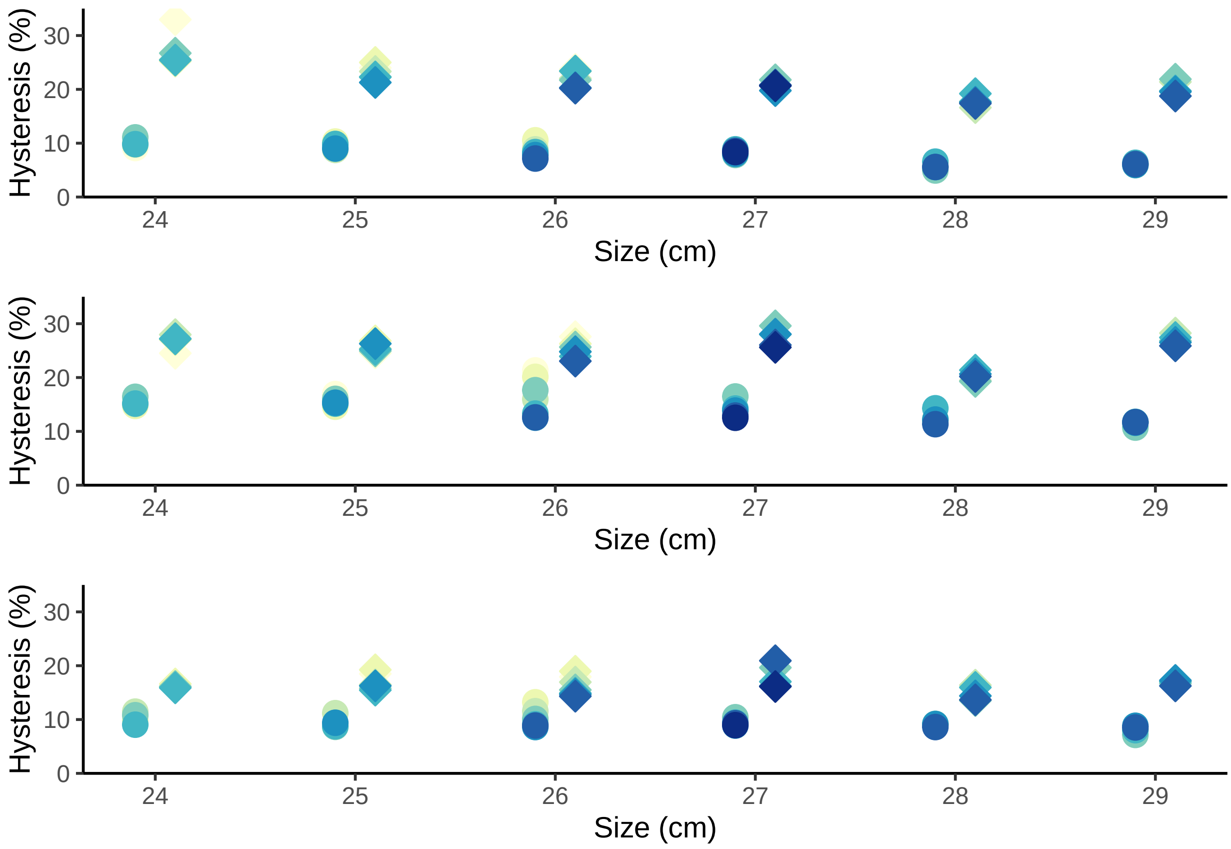


```r
ggsave("fig9.pdf", plot = fig9, device = "pdf", useDingbats = FALSE, width = 3.25, height = 8, units =
```

Statistics

Table 2: Linear Regression Models for the effects of category, size, and shoe/no shoe on axial stiffness

```r
#Heel (stiff category as numerical)
heel.axial <- lm(Kavg_load ~ Stiff_num + Size + No_Shoe.Shoe, data = heel_data)
summary(heel.axial)
```

```
##
## Call:
## lm(formula = Kavg_load ~ Stiff_num + Size + No_Shoe.Shoe, data = heel_data)
##
## Residuals:
##     Min      1Q  Median      3Q     Max
## -8.4415 -2.1428 -0.1635  1.9002  9.2631
##
## Coefficients:
##                    Estimate Std. Error t value Pr(>|t|)
## (Intercept)         72.8462     6.7547  10.785 7.43e-16 ***
## Stiff_num            4.6434     0.2349  19.770  < 2e-16 ***
## Size                -1.6684     0.2684  -6.215 4.80e-08 ***
## No_Shoe.ShoeShoe -13.5145     0.8062 -16.764  < 2e-16 ***
```

```
## ---
## Signif. codes:  0 '***' 0.001 '**' 0.01 '*' 0.05 '.' 0.1 ' ' 1
##
## Residual standard error: 3.275 on 62 degrees of freedom
## Multiple R-squared:  0.9161, Adjusted R-squared:  0.912
## F-statistic: 225.6 on 3 and 62 DF,  p-value: < 2.2e-16
```

```r
confint(heel.axial)
```

```
##                        2.5 %     97.5 %
## (Intercept)       59.343751  86.348680
## Stiff_num          4.173895   5.112896
## Size              -2.204973  -1.131802
## No_Shoe.ShoeShoe -15.126026 -11.903065
```

```r
r2(heel.axial)
```

```
## # R2 for Linear Regression
##        R2: 0.916
##   adj. R2: 0.912
```

```r
#plot(heel.axial, which = 1)
#plot(heel.axial, which = 2)
#plot(heel.axial, which = 3)
#plot(heel.axial, which = 5)

#Midfoot (stiff category as numerical)
midfoot.axial <- lm(Kavg_load ~ Stiff_num + Size + No_Shoe.Shoe, data = midfoot_data)
summary(midfoot.axial)
```

```
##
## Call:
## lm(formula = Kavg_load ~ Stiff_num + Size + No_Shoe.Shoe, data = midfoot_data)
##
## Residuals:
##     Min      1Q  Median      3Q     Max
## -43.488 -16.304   0.617  16.506  51.941
##
## Coefficients:
##                   Estimate Std. Error t value Pr(>|t|)
## (Intercept)        635.261     47.978  13.241  < 2e-16 ***
## Stiff_num           15.627      1.668   9.367 1.75e-13 ***
## Size               -19.394      1.907 -10.172 7.70e-15 ***
## No_Shoe.ShoeShoe   -81.352      5.726 -14.207  < 2e-16 ***
## ---
## Signif. codes:  0 '***' 0.001 '**' 0.01 '*' 0.05 '.' 0.1 ' ' 1
##
## Residual standard error: 23.26 on 62 degrees of freedom
## Multiple R-squared:  0.8447, Adjusted R-squared:  0.8371
## F-statistic: 112.4 on 3 and 62 DF,  p-value: < 2.2e-16
```

```r
confint(midfoot.axial)
```

```
##                        2.5 %    97.5 %
## (Intercept)      539.35341 731.16865
## Stiff_num         12.29196  18.96166
## Size             -23.20521 -15.58250
## No_Shoe.ShoeShoe -92.79812 -69.90552
```

```r
r2(midfoot.axial)
```

```
## # R2 for Linear Regression
##        R2: 0.845
##   adj. R2: 0.837
```

```r
#plot(midfoot.axial, which = 1)
#plot(midfoot.axial, which = 2)
#plot(midfoot.axial, which = 3)
#plot(midfoot.axial, which = 5)

#Forefoot (stiff category as numerical)
forefoot.axial <- lm(Kavg_load ~ Stiff_num + Size + No_Shoe.Shoe, data = forefoot_data)
summary(forefoot.axial)
```

```
##
## Call:
## lm(formula = Kavg_load ~ Stiff_num + Size + No_Shoe.Shoe, data = forefoot_data)
##
## Residuals:
##     Min      1Q  Median      3Q     Max
## -6.2938 -2.1224  0.3352  1.7076  7.3991
##
## Coefficients:
##                  Estimate Std. Error t value Pr(>|t|)
## (Intercept)       66.4749     6.4310  10.337 4.09e-15 ***
## Stiff_num          3.8414     0.2236  17.179  < 2e-16 ***
## Size              -1.6321     0.2556  -6.386 2.45e-08 ***
## No_Shoe.ShoeShoe   0.5655     0.7675   0.737    0.464
## ---
## Signif. codes:  0 '***' 0.001 '**' 0.01 '*' 0.05 '.' 0.1 ' ' 1
##
## Residual standard error: 3.118 on 62 degrees of freedom
## Multiple R-squared:  0.827,  Adjusted R-squared:  0.8186
## F-statistic: 98.79 on 3 and 62 DF,  p-value: < 2.2e-16
```

```r
confint(forefoot.axial)
```

```
##                       2.5 %    97.5 %
## (Intercept)      53.6195540 79.330163
## Stiff_num         3.3944073  4.288402
## Size             -2.1429923 -1.121257
## No_Shoe.ShoeShoe -0.9687895  2.099699
```

```r
r2(forefoot.axial)
```

```
## # R2 for Linear Regression
##        R2: 0.827
##   adj. R2: 0.819
```

```r
#plot(forefoot.axial, which = 1)
#plot(forefoot.axial, which = 2)
#plot(forefoot.axial, which = 3)
#plot(forefoot.axial, which = 5)
```

Table 3: Linear Regression Models for the effects of category, size, and shoe/no shoe on torsional stiffness

```r
#Heel (stiff category as numerical)
heel.torsional <- lm(Ktors_avg_load ~ Stiff_num + Size + No_Shoe.Shoe, data = heel_data)
summary(heel.torsional)
```

```
##
## Call:
## lm(formula = Ktors_avg_load ~ Stiff_num + Size + No_Shoe.Shoe,
##     data = heel_data)
##
## Residuals:
##       Min        1Q    Median        3Q       Max
## -0.030688 -0.007736 -0.001813  0.005458  0.045128
##
## Coefficients:
##                   Estimate Std. Error t value Pr(>|t|)
## (Intercept)      -0.361882   0.028817  -12.56   <2e-16 ***
## Stiff_num         0.011877   0.001002   11.85   <2e-16 ***
## Size              0.016676   0.001145   14.56   <2e-16 ***
## No_Shoe.ShoeShoe -0.040000   0.003439  -11.63   <2e-16 ***
## ---
## Signif. codes:  0 '***' 0.001 '**' 0.01 '*' 0.05 '.' 0.1 ' ' 1
##
## Residual standard error: 0.01397 on 62 degrees of freedom
## Multiple R-squared:  0.9223, Adjusted R-squared:  0.9186
## F-statistic: 245.4 on 3 and 62 DF,  p-value: < 2.2e-16
```

```r
confint(heel.torsional)
```

```
##                          2.5 %      97.5 %
## (Intercept)      -0.419487092 -0.30427672
## Stiff_num         0.009873906  0.01387994
## Size              0.014387185  0.01896563
## No_Shoe.ShoeShoe -0.046875015 -0.03312498
```

```r
r2(heel.torsional)
```

```
## # R2 for Linear Regression
##        R2: 0.922
##   adj. R2: 0.919
```

```r
#plot(heel.torsional, which = 1)
#plot(heel.torsional, which = 2)
#plot(heel.torsional, which = 3)
#plot(heel.torsional, which = 5)

#Forefoot (stiff category as numerical)
forefoot.torsional <- lm(Ktors_avg_load ~ Stiff_num + Size + No_Shoe.Shoe, data = forefoot_data)
summary(forefoot.torsional)
```

```
##
## Call:
## lm(formula = Ktors_avg_load ~ Stiff_num + Size + No_Shoe.Shoe,
##     data = forefoot_data)
##
## Residuals:
##      Min       1Q   Median       3Q      Max
## -0.22656 -0.06241 -0.00728  0.06538  0.34329
##
## Coefficients:
##                   Estimate Std. Error t value Pr(>|t|)
## (Intercept)      -1.649151   0.225809  -7.303 6.41e-10 ***
## Stiff_num         0.123668   0.007852  15.750  < 2e-16 ***
## Size              0.092275   0.008974  10.283 5.02e-15 ***
## No_Shoe.ShoeShoe  0.027879   0.026950   1.034    0.305
## ---
## Signif. codes:  0 '***' 0.001 '**' 0.01 '*' 0.05 '.' 0.1 ' ' 1
##
## Residual standard error: 0.1095 on 62 degrees of freedom
## Multiple R-squared:  0.9053, Adjusted R-squared:  0.9007
## F-statistic: 197.6 on 3 and 62 DF,  p-value: < 2.2e-16
```

```r
confint(forefoot.torsional)
```

```
##                        2.5 %     97.5 %
## (Intercept)      -2.10053746 -1.1977642
## Stiff_num         0.10797248  0.1393632
## Size              0.07433698  0.1102130
## No_Shoe.ShoeShoe -0.02599292  0.0817505
```

```r
r2(forefoot.torsional)
```

```
## # R2 for Linear Regression
##        R2: 0.905
##   adj. R2: 0.901
```

```r
#plot(forefoot.torsional, which = 1)
#plot(forefoot.torsional, which = 2)
#plot(forefoot.torsional, which = 3)
#plot(forefoot.torsional, which = 5)
```

Table 4: Linear Regression Models for the effects of category, size, and shoe/no shoe on hysteresis

```r
#Heel (stiff category as numerical)
heel.hys <- lm(Per_hysteresis ~ Stiff_num + Size + No_Shoe.Shoe, data = heel_data)
summary(heel.hys)
```

```
##
## Call:
## lm(formula = Per_hysteresis ~ Stiff_num + Size + No_Shoe.Shoe,
##     data = heel_data)
##
## Residuals:
##     Min      1Q  Median      3Q     Max
## -4.0857 -0.8808  0.0141  0.7122  7.5370
##
## Coefficients:
##                  Estimate Std. Error t value Pr(>|t|)
## (Intercept)       36.5577     3.3060  11.058 2.65e-16 ***
## Stiff_num         -0.3109     0.1150  -2.704  0.00882 **
## Size              -1.0264     0.1314  -7.812 8.38e-11 ***
## No_Shoe.ShoeShoe  13.8094     0.3946  35.000  < 2e-16 ***
## ---
## Signif. codes:  0 '***' 0.001 '**' 0.01 '*' 0.05 '.' 0.1 ' ' 1
##
## Residual standard error: 1.603 on 62 degrees of freedom
## Multiple R-squared:  0.9554, Adjusted R-squared:  0.9533
## F-statistic:   443 on 3 and 62 DF,  p-value: < 2.2e-16
```

```r
confint(heel.hys)
```

```
##                       2.5 %      97.5 %
## (Intercept)      29.9491211 43.16623776
## Stiff_num        -0.5406758 -0.08109754
## Size             -1.2890058 -0.76375968
## No_Shoe.ShoeShoe 13.0206813 14.59810658
```

```r
r2(heel.hys)
```

```
## # R2 for Linear Regression
##        R2: 0.955
##   adj. R2: 0.953
```

```r
#plot(heel.hys, which = 1)
#plot(heel.hys, which = 2)
#plot(heel.hys, which = 3)
#plot(heel.hys, which = 5)

#Midfoot (stiff category as numerical)
midfoot.hys <- lm(Per_hysteresis ~ Stiff_num + Size + No_Shoe.Shoe, data = midfoot_data)
summary(midfoot.hys)
```

```
##
## Call:
## lm(formula = Per_hysteresis ~ Stiff_num + Size + No_Shoe.Shoe,
##     data = midfoot_data)
##
## Residuals:
##     Min      1Q  Median      3Q     Max
## -5.5950 -1.2416 -0.1675  0.9206  5.6396
##
## Coefficients:
##                  Estimate Std. Error t value Pr(>|t|)
## (Intercept)       28.4001     4.7234   6.013 1.06e-07 ***
## Stiff_num         -0.3363     0.1642  -2.048   0.0448 *
## Size              -0.4759     0.1877  -2.535   0.0138 *
## No_Shoe.ShoeShoe  10.9891     0.5637  19.494  < 2e-16 ***
## ---
## Signif. codes:  0 '***' 0.001 '**' 0.01 '*' 0.05 '.' 0.1 ' ' 1
##
## Residual standard error: 2.29 on 62 degrees of freedom
## Multiple R-squared:  0.8652, Adjusted R-squared:  0.8587
## F-statistic: 132.7 on 3 and 62 DF,  p-value: < 2.2e-16
```

```
confint(midfoot.hys)
```

```
##                       2.5 %       97.5 %
## (Intercept)      18.9581567 37.842108463
## Stiff_num        -0.6646126 -0.007990253
## Size             -0.8511251 -0.100679877
## No_Shoe.ShoeShoe  9.8622179 12.115963879
```

```
r2(midfoot.hys)
```

```
## # R2 for Linear Regression
##        R2: 0.865
##   adj. R2: 0.859
```

```
#plot(midfoot.hys, which = 1)
#plot(midfoot.hys, which = 2)
#plot(midfoot.hys, which = 3)
#plot(midfoot.hys, which = 5)

#forefoot (stiff category as numerical)
forefoot.hys <- lm(Per_hysteresis ~ Stiff_num + Size + No_Shoe.Shoe, data = forefoot_data)
summary(forefoot.hys)
```

```
##
## Call:
## lm(formula = Per_hysteresis ~ Stiff_num + Size + No_Shoe.Shoe,
##     data = forefoot_data)
##
## Residuals:
##     Min      1Q  Median      3Q     Max
```

```
## -2.9277 -0.8447 -0.0446  0.5662  5.2106
##
## Coefficients:
##                   Estimate Std. Error t value Pr(>|t|)
## (Intercept)       12.77817    2.78080   4.595 2.18e-05 ***
## Stiff_num         -0.27568    0.09669  -2.851  0.00591 **
## Size              -0.07873    0.11051  -0.712  0.47888
## No_Shoe.ShoeShoe   6.98667    0.33188  21.052  < 2e-16 ***
## ---
## Signif. codes:  0 '***' 0.001 '**' 0.01 '*' 0.05 '.' 0.1 ' ' 1
##
## Residual standard error: 1.348 on 62 degrees of freedom
## Multiple R-squared:  0.8802, Adjusted R-squared:  0.8744
## F-statistic: 151.9 on 3 and 62 DF,  p-value: < 2.2e-16
```

```r
confint(forefoot.hys)
```

```
##                       2.5 %      97.5 %
## (Intercept)       7.2194209 18.33690960
## Stiff_num        -0.4689656 -0.08239448
## Size             -0.2996324  0.14217480
## No_Shoe.ShoeShoe  6.3232463  7.65008699
```

```r
r2(forefoot.hys)
```

```
## # R2 for Linear Regression
##        R2: 0.880
##   adj. R2: 0.874
```

```r
#plot(forefoot.hys, which = 1)
#plot(forefoot.hys, which = 2)
#plot(forefoot.hys, which = 3)
#plot(forefoot.hys, which = 5)
```

Average values

```r
avg_r2 <- stiff_data %>%
  summarise(
    linear_r2_avg = mean(linear_adj_rsq),
    quad_r2_avg = mean(quad_adj_rsq),
    linear_r2_tors_avg = mean(ang_linear_adj_rsq, na.rm = TRUE),
    quad_r2_tors_avg = mean(ang_quad_adj_rsq, na.rm = TRUE)
  )

avg_stiff <- stiff_data %>%
  group_by(Stiff_cat, Test, No_Shoe.Shoe) %>%
  summarise(
    kavg = mean(Kavg_load),
    hys_avg = mean(Per_hysteresis)
  )
```

```
## `summarise()` has grouped output by 'Stiff_cat', 'Test'. You can override using
## the `.groups` argument.
```

```r
max(avg_stiff$hys_avg)
```

```
## [1] 26.89333
```

```r
avg_stiff_size <- stiff_data %>%
  group_by(Stiff_cat, Test, No_Shoe.Shoe, Size) %>%
  summarise(
    kavg = mean(Kavg_load)
  )
```

```
## `summarise()` has grouped output by 'Stiff_cat', 'Test', 'No_Shoe.Shoe'. You
## can override using the `.groups` argument.
```

```r
heel_27_cat5_noshoe <- avg_stiff_size[avg_stiff_size$Test == "heel" & avg_stiff_size$Stiff_cat == "cat5

heel_27_cat7_noshoe <- avg_stiff_size[avg_stiff_size$Test == "heel" & avg_stiff_size$Stiff_cat == "cat7

forefoot_27_cat4_noshoe <- avg_stiff_size[avg_stiff_size$Test == "forefoot" & avg_stiff_size$Stiff_cat =

forefoot_27_cat7_noshoe <- avg_stiff_size[avg_stiff_size$Test == "forefoot" & avg_stiff_size$Stiff_cat =

avg_ang_stiff_noshoe_hindfoot <- heel_data[heel_data$No_Shoe.Shoe == "No Shoe",]%>%
  group_by(Stiff_cat, Size) %>%
  summarise(
    kang = mean(Ktors_avg_load),
  )
```

```
## `summarise()` has grouped output by 'Stiff_cat'. You can override using the
## `.groups` argument.
```

```r
max(avg_ang_stiff_noshoe_hindfoot$kang)
```

```
## [1] 0.25
```

```r
min(avg_ang_stiff_noshoe_hindfoot$kang)
```

```
## [1] 0.05
```

```r
avg_ang_stiff_noshoe_forefoot <- forefoot_data[forefoot_data$No_Shoe.Shoe == "No Shoe",]%>%
  group_by(Stiff_cat, Size) %>%
  summarise(
    kang = mean(Ktors_avg_load),
  )
```

```
## `summarise()` has grouped output by 'Stiff_cat'. You can override using the
## `.groups` argument.
```

```r
max(avg_ang_stiff_noshoe_forefoot$kang)
```

```
## [1] 1.98
```

```r
min(avg_ang_stiff_noshoe_forefoot$kang)
```

```
## [1] 0.72
```
