# Supplementary material for: Low-profile prosthetic foot stiffness category and size, and shoes affect axial and torsional stiffness and hysteresis
Source: Front Rehabil Sci. 2024 Feb 28;5:1290092. doi: 10.3389/fresc.2024.1290092 (PMC10932964; doi:10.3389/fresc.2024.1290092)
Supplement: Supplementary file 3 [file Datasheet3.docx]

**Derivation and Verification of Equations 1–3**

The force applied onto the prosthetic foot from the custom base and low-friction roller system is the normal force (F_norm_), which is perpendicular to the surface of the locked rotatable base (Supplementary Material Figure 1). F_norm_ equals the vector sum of the vertical force applied (F_MTM_) by the Instron materials testing machine (MTM) onto the pylon of the prosthesis and a reaction force (F_rxn_) on the MTM due to the angle of the surface (θ) and the low friction roller system (Supplementary Material Figure 1). We derived equation 1 by solving the static equilibrium equations (Supplementary Material Figure 1):

$F_{norm}= \frac{F_{MTM}}{\cos\theta}.$ (Eqn. 1)


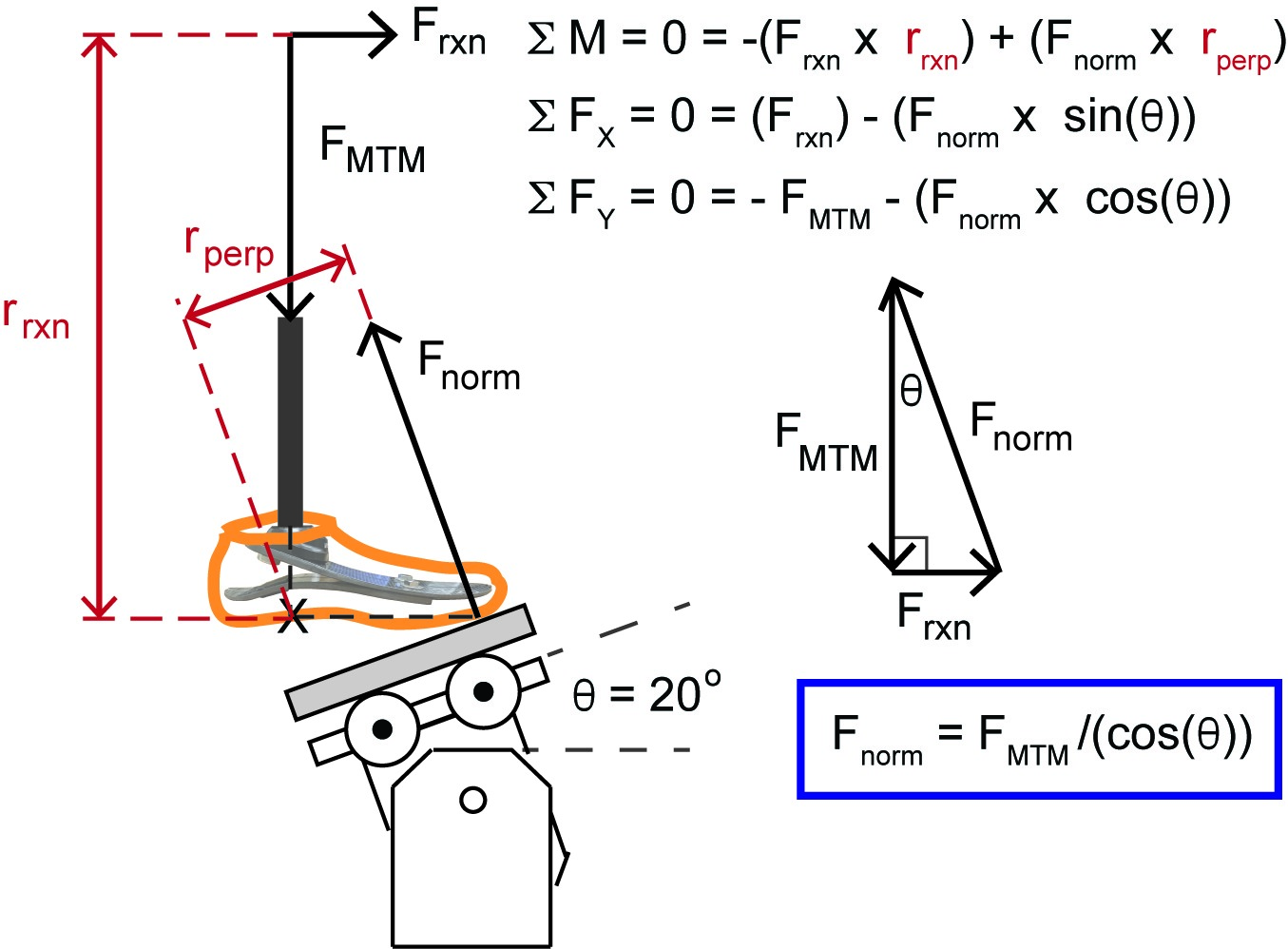


**Supplementary Material Figure 1.** Free body diagram and derivation of equation (1) for the forefoot stiffness test. During the test the materials testing machine (MTM) applied a downward vertical force onto the pylon of the prosthesis (F_MTM_). The prosthetic foot came in contact with the low friction roller system on the locked rotatable base set at angle (θ) of -15° for the heel stiffness test and 20° for the forefoot stiffness test. The force applied to the prosthesis is the normal force perpendicular to the rotatable base surface (F_norm_) and equals $\frac{F_{MTM}}{cos\theta}$. F_norm_ is the vector sum of F_MTM_ and a reaction force (F_rxn_) applied to the load cell of the MTM.


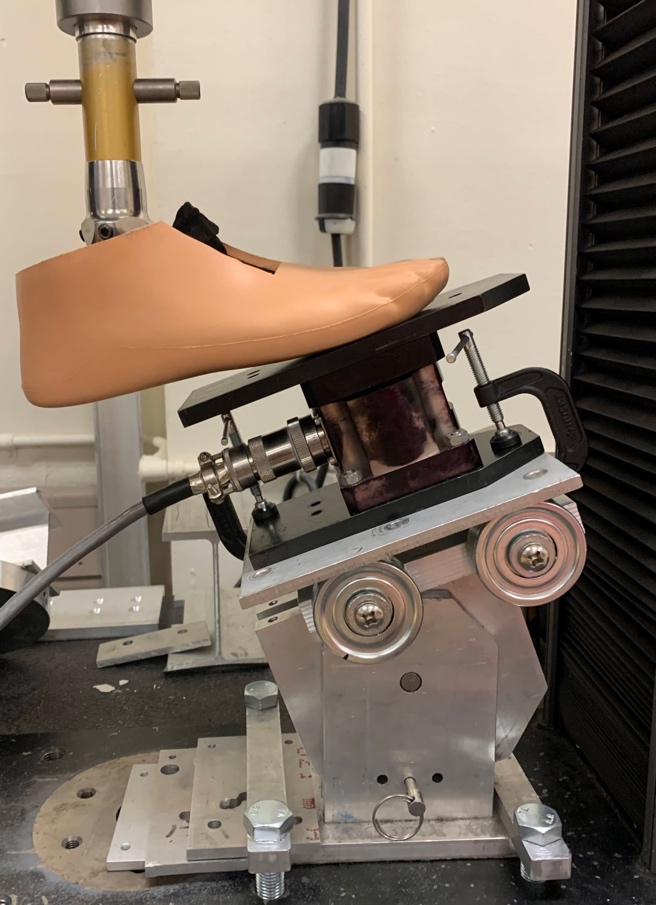
To verify our derivation of equation 1, we placed a 3-axis force transducer (MC3A-500, AMTI, Watertown, MA, USA) sampling at 1000 Hz beneath the prosthetic foot to measure F_norm_ (Supplementary Material Figure 2). We conducted a forefoot stiffness test with the category 3, size 25 LP Vari-flex (Össur, Reykjavik, Iceland) prosthetic foot without a shoe. We applied F_MTM_ onto the pylon of the prosthesis at a rate of 100 N/s for 4 loading and unloading cycles. During the test, the prosthetic foot contacted the force transducer that we placed on top of the low-friction roller system and the rotatable base set to 20°. The average maximum F_MTM_ was 675.1 N, while F_norm_ measured directly from the force transducer (F_norm,ft_) was 711.7 N. Using F_MTM_ and equation (1), we estimated the normal force applied to the prosthesis (F_norm,est_). The average maximum F_norm,est_ was 718.4 N. Since F_norm,ft_ $\approx$ F_norm,est_, we verified equation 1 and used equation 1 for all heel and forefoot stiffness calculations reported in the manuscript.

**Supplementary Material Figure 2.** We placed a force transducer (MC3A-500, AMTI, Waterford, MA, USA) between the category 3, size 25 LP Vari-flex (Össur, Reykjavik, Iceland) prosthetic foot without a shoe and the locked rotatable base and low-friction roller system. We applied force with the materials testing machine to the pylon of the prosthesis at 100 N/s. The rotatable base was set to 20°. To verify equation 1, we measured the normal force applied to the prosthesis (F_norm_) directly from the force transducer and compared F_norm_ to the value calculated from the materials testing machine force (F_MTM_) and equation 1 (F_norm_ = $\frac{F_{MTM}}{cos\theta}$).

The displacement of the prosthetic foot normal to the surface of the custom base and low-friction roller system is the normal displacement (d_norm_), which is perpendicular to the surface of the locked rotatable base (Supplementary Material Figure 3). Since the low-friction roller system allows the prosthetic foot to move parallel to the surface (d_par_), d_norm_ equals the product of the vertical displacement of the MTM (d_MTM_) and the cosine of the angle of the rotatable base (θ)(Eqn. 2); Supplementary Material Figure 3):

$d_{norm}= d_{MTM}\cos\theta$. (Eqn. 2)

Therefore, the axial stiffness of the prosthesis (k) equals the quotient of F_MTM_ and the product of d_MTM_ and cos($\theta$)^2^:

$k = \frac{F_{norm}}{d_{norm}}= \frac{F_{MTM}}{d_{MTM}\left( \cos\theta\right)^{2}}$. (Eqn. 3)


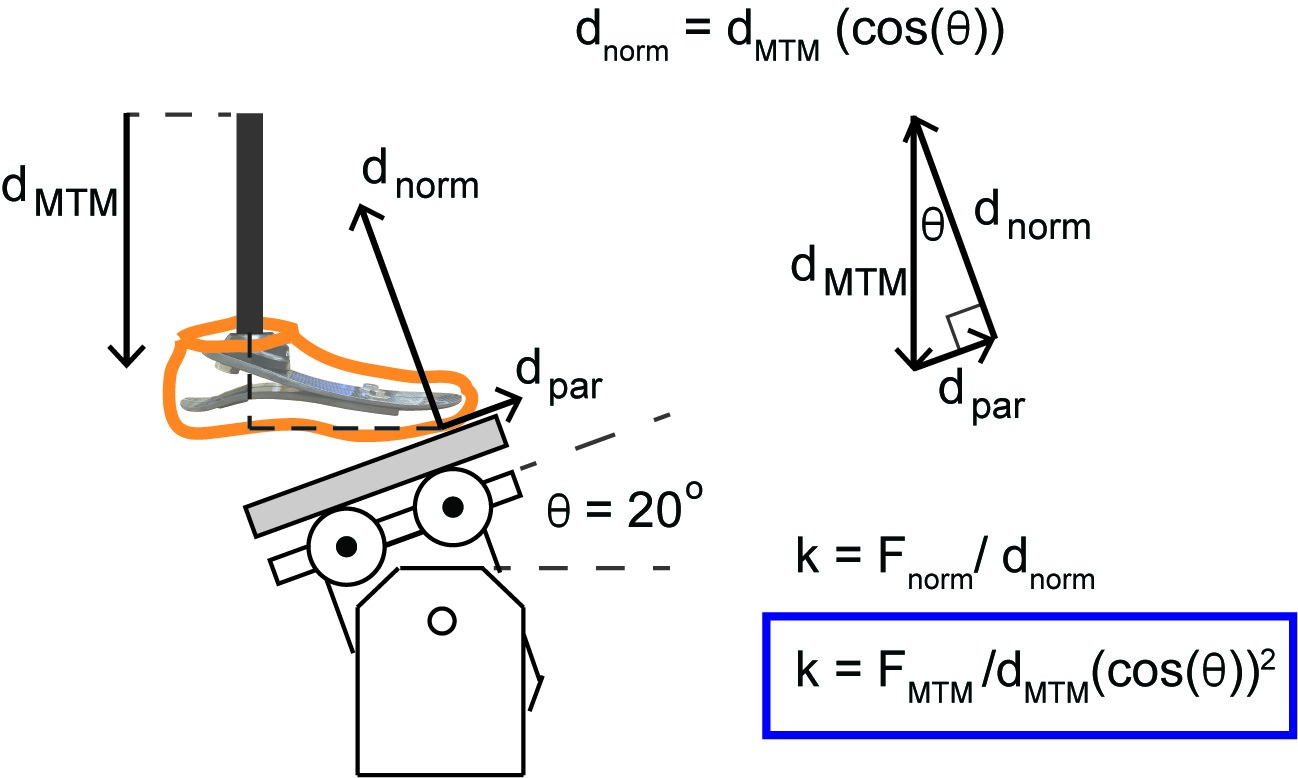


**Supplementary Material Figure 3.** Free body diagram and derivations of equations 2 and 3 for the forefoot stiffness test. During the test the materials testing machine (MTM) applied a vertical force onto the pylon of the prosthesis (F_MTM_) displacing the pylon vertically (d_MTM_). The prosthetic foot came in contact with the low-friction roller system on the locked rotatable base set at angle (θ) of -15° for the heel stiffness test and 20° for the forefoot stiffness test. The low-friction roller system allowed the prosthesis to displace parallel to the surface (d_par_). Thus, the displacement of the prosthesis perpendicular to the surface (d_norm_) equals $d_{MTM}cos\theta$ and the axial stiffness (k) equals $\frac{F_{MTM}}{d_{MTM}\left( \cos\theta\right)^{2}} .$
