# Supplementary material for: Low-profile prosthetic foot stiffness category and size, and shoes affect axial and torsional stiffness and hysteresis
Source: Front Rehabil Sci. 2024 Feb 28;5:1290092. doi: 10.3389/fresc.2024.1290092 (PMC10932964; doi:10.3389/fresc.2024.1290092)
Supplement: Supplementary file 4 [file Datasheet4.docx]

Information about Data.csv

Columns

- Test: stiffness test– heel, midfoot, or forefoot
- Stiff_cat: stiffness category– cat1, cat2, cat3, cat4, cat5, cat6, cat7, or cat8
- Size: size of the prosthetic foot in cm
- No_Shoe/Shoe: whether test was with a shoe or not
- Kavg_load: average axial stiffness in kN/m during loading phase
- Kavg_unload: average axial stiffness in kN/m during unloading phase
- Per_Hysteresis: hysteresis in %
- linear_adj_rsq: adjusted R^2^ value for linear fit of force-displacement equations
- quad_adj_rsq: adjusted R^2^ value for quadratic fit of force-displacement equations
- Ktors_avg_load: average torsional stiffness in kN-m/rad during loading phase
- Ktors_avg_unload: average torsional stiffness in kN-m/rad during unloading phase
- tors_linear_adj_rsq: adjusted R^2^ value for linear fit of torque-angle equations
- tors_quad_adj_rsq: adjusted R^2^ value for quadratic fit of torque-angle equations
