# Supplementary material for: Low-profile prosthetic foot stiffness category and size, and shoes affect axial and torsional stiffness and hysteresis
Source: Front Rehabil Sci. 2024 Feb 28;5:1290092. doi: 10.3389/fresc.2024.1290092 (PMC10932964; doi:10.3389/fresc.2024.1290092)
Supplement: Supplementary file 5 [file Datasheet5.docx]

| **Heel Force-Displacement Coefficients; No Shoe**  (f = ax^2^ + bx + c) | | | | | | | |
| --- | --- | --- | --- | --- | --- | --- | --- |
|  |  | **Size** | | | | | |
| **Category** | **Coefficient** | **24** | **25** | **26** | **27** | **28** | **29** |
| **1** | **a** | 2.63E+03 | 1.75E+03 | 1.96E+03 | – | – | – |
|  | **b** | -5.94E+00 | 3.27E+00 | -4.90E+00 | – | – | – |
|  | **c** | 5.88E-02 | 2.36E-02 | 6.10E-02 | – | – | – |
| **2** | **a** | 2.01E+03 | 2.68E+03 | 1.44E+03 | – | – | – |
|  | **b** | 6.06E+00 | 1.15E+00 | 1.32E+01 | – | – | – |
|  | **c** | 4.36E-02 | 3.98E-02 | -1.68E-04 | – | – | – |
| **3** | **a** | 2.02E+03 | 2.39E+03 | 3.14E+03 | – | 1.16E+03 | 1.24E+03 |
|  | **b** | 1.12E+01 | 8.82E-01 | -9.21E+00 | – | 1.94E+00 | 8.62E+00 |
|  | **c** | 3.46E-02 | 3.88E-02 | 6.76E-02 | – | 3.54E-02 | 3.09E-02 |
| **4** | **a** | 3.40E+03 | 2.62E+03 | 2.62E+03 | 2.19E+03 | 1.65E+03 | 1.45E+03 |
|  | **b** | -4.69E+00 | 1.04E+01 | -7.52E-02 | 4.58E+00 | 3.21E+00 | 1.20E+01 |
|  | **c** | 7.58E-02 | 2.69E-02 | 6.10E-02 | 4.82E-02 | 4.44E-02 | 2.91E-02 |
| **5** | **a** | 3.00E+03 | 3.14E+03 | 3.59E+03 | 2.53E+03 | 2.86E+03 | 2.63E+03 |
|  | **b** | 1.00E+01 | 6.35E+00 | -8.19E+00 | 1.18E+00 | -1.16E+00 | -2.00E+00 |
|  | **c** | 5.83E-02 | 6.73E-02 | 8.48E-02 | 6.72E-02 | 5.17E-02 | 7.39E-02 |
| **6** | **a** | – | 3.08E+03 | 2.92E+03 | 2.09E+03 | 2.83E+03 | 2.67E+03 |
|  | **b** | – | 2.09E+01 | 3.81E+00 | 8.53E+00 | -8.72E+00 | 6.99E+00 |
|  | **c** | – | 2.86E-02 | 6.47E-02 | 4.58E-02 | 8.43E-02 | 6.23E-02 |
| **7** | **a** | – | – | 3.18E+03 | 2.92E+03 | 3.05E+03 | 2.69E+03 |
|  | **b** | – | – | 9.18E+00 | -2.13E+00 | -6.53E+00 | 5.20E+00 |
|  | **c** | – | – | 4.98E-02 | 7.46E-02 | 8.80E-02 | 6.49E-02 |
| **8** | **a** | – | – | – | 3.17E+03 | – | – |
|  | **b** | – | – | – | 2.30E+00 | – | – |
|  | **c** | – | – | – | 7.05E-02 | – | – |

Loading Phase

**Supplementary Material Table 1.** Coefficients for the force-displacement equations during the loading phase at the heel for the LP Vari-flex prosthetic feet of different categories and sizes without a shoe. Equations are in the form of f = ax^2^ + bx + c where f is the force (kN), x is the displacement (m), and a (kN m^-2^), b (kN m^-1^), and c (kN) are coefficients.

**Supplementary Material Table 2.** Coefficients for the force-displacement equations during the loading phase at the midfoot for the LP Vari-flex feet of different categories and sizes without a shoe. Equations are in the form of f = ax^2^ + bx + c where f is the force (kN), x is the displacement (m), and a (kN m^-2^), b (kN m^-1^), and c (kN) are coefficients.

| **Midfoot Force-Displacement Coefficients; No Shoe**  (ax^2^ + bx + c = f) | | | | | | | |
| --- | --- | --- | --- | --- | --- | --- | --- |
|  |  | **Size** | | | | | |
| **Category** | **Coefficient** | **24** | **25** | **26** | **27** | **28** | **29** |
| **1** | **a** | 3.45E+04 | 3.92E+04 | 2.13E+04 | – | – | – |
|  | **b** | 8.21E+01 | 7.91E+00 | -1.35E+01 | – | – | – |
|  | **c** | 5.49E-04 | 1.29E-02 | 5.65E-02 | – | – | – |
| **2** | **a** | 5.14E+04 | 5.37E+04 | 2.89E+04 | – | – | – |
|  | **b** | 1.58E+01 | 1.53E+01 | -3.78E+01 | – | – | – |
|  | **c** | 1.23E-02 | 2.81E-02 | 6.23E-02 | – | – | – |
| **3** | **a** | 4.90E+04 | 3.16E+04 | 4.14E+04 | – | 1.64E+04 | 1.73E+04 |
|  | **b** | 1.03E+02 | 4.36E+01 | -3.69E+01 | – | -2.11E+01 | -4.37E+01 |
|  | **c** | 1.32E-04 | 1.90E-02 | 4.32E-02 | – | 5.44E-02 | 7.08E-02 |
| **4** | **a** | 4.56E+04 | 4.19E+04 | 3.58E+04 | 2.36E+04 | 1.56E+04 | 1.68E+04 |
|  | **b** | 1.32E+02 | 5.43E+01 | -4.53E+01 | -2.56E+01 | 1.09E+01 | -4.47E+01 |
|  | **c** | -1.62E-02 | 1.57E-02 | 4.06E-02 | 4.55E-02 | 4.06E-02 | 7.34E-02 |
| **5** | **a** | 4.39E+04 | 5.81E+04 | 3.90E+04 | 2.19E+04 | 2.48E+04 | 1.92E+04 |
|  | **b** | 1.39E+02 | 4.48E+01 | 7.50E+00 | -1.62E+01 | 2.58E+01 | 1.21E+01 |
|  | **c** | -1.92E-02 | -9.46E-03 | -1.20E-03 | 2.62E-02 | 1.73E-02 | -2.71E-04 |
| **6** | **a** | – | 4.50E+04 | 3.73E+04 | 2.32E+04 | 2.06E+04 | 2.18E+04 |
|  | **b** | – | 3.90E+01 | 3.61E+01 | -1.93E+01 | 7.89E+01 | 3.82E+01 |
|  | **c** | – | 3.80E-02 | -9.06E-03 | 2.96E-02 | -3.04E-02 | -2.82E-03 |
| **7** | **a** | – | – | 4.44E+04 | 2.43E+04 | 1.62E+04 | 1.96E+04 |
|  | **b** | – | – | -3.13E+01 | 4.46E+01 | 1.32E+02 | 4.49E+01 |
|  | **c** | – | – | 2.47E-02 | 1.29E-02 | -5.17E-02 | -7.66E-02 |
| **8** | **a** | – | – | – | 2.72E+04 | – | – |
|  | **b** | – | – | – | 6.19E+01 | – | – |
|  | **c** | – | – | – | 3.37E-03 | – | – |

**Supplementary Material Table 3.** Coefficients for the force-displacement equations during the loading phase at the forefoot for the LP Vari-flex feet of different categories and sizes without a shoe. Equations are in the form of f = ax^2^ + bx + c where f is the force (kN), x is the displacement (m), and a (kN m^-2^), b (kN m^-1^), and c (kN) are coefficients.

| **Forefoot Force-Displacement Coefficients; No Shoe**  (ax^2^ + bx + c = f) | | | | | | | |
| --- | --- | --- | --- | --- | --- | --- | --- |
|  | | **Size** | | | | | |
| **Category** | **Coefficient** | **24** | **25** | **26** | **27** | **28** | **29** |
| **1** | **a** | 1.51E+03 | 1.16E+03 | 1.57E+03 | – | – | – |
|  | **b** | 1.85E+00 | -1.13E+00 | -3.27E+00 | – | – | – |
|  | **c** | 2.62E-02 | 4.24E-02 | 3.88E-02 | – | – | – |
| **2** | **a** | 2.01E+03 | 1.30E+03 | 2.03E+03 | – | – | – |
|  | **b** | -4.54E+00 | 6.25E-01 | -6.04E+00 | – | – | – |
|  | **c** | 4.53E-02 | 3.32E-02 | 4.70E-02 | – | – | – |
| **3** | **a** | 2.60E+03 | 1.03E+03 | 1.59E+03 | – | 1.43E+03 | 1.37E+03 |
|  | **b** | -1.99E+00 | 1.04E+01 | -2.04E+00 | – | -6.28E-01 | 3.02E+00 |
|  | **c** | 3.41E-02 | 1.72E-02 | 3.96E-02 | – | 3.37E-02 | 3.22E-02 |
| **4** | **a** | 2.88E+03 | 1.73E+03 | 1.78E+03 | 1.48E+03 | 1.27E+03 | 1.45E+03 |
|  | **b** | -6.81E+00 | -1.44E-01 | -1.27E+00 | -2.48E+00 | 1.28E+00 | 4.99E+00 |
|  | **c** | 5.33E-02 | 5.18E-02 | 4.14E-02 | 4.62E-02 | 4.22E-02 | 3.50E-02 |
| **5** | **a** | 2.99E+03 | 1.67E+03 | 1.70E+03 | 1.39E+03 | 1.18E+03 | 1.40E+03 |
|  | **b** | -1.68E+00 | 5.37E+00 | 5.54E-01 | -2.00E+00 | 6.99E+00 | 2.08E+00 |
|  | **c** | 4.60E-02 | 4.60E-02 | 4.40E-02 | 6.38E-02 | 4.28E-02 | 4.59E-02 |
| **6** | **a** | – | 2.18E+03 | 1.81E+03 | 1.58E+03 | 1.15E+03 | 1.52E+03 |
|  | **b** | – | 6.95E+00 | 1.00E+00 | -1.67E+00 | 4.40E+00 | 3.31E+00 |
|  | **c** | – | 4.37E-02 | 4.94E-02 | 6.19E-02 | 5.52E-02 | 4.59E-02 |
| **7** | **a** | – | – | 2.14E+03 | 1.83E+03 | 1.50E+03 | 1.57E+03 |
|  | **b** | – | – | 5.75E+00 | -2.54E+00 | 1.04E+01 | 5.28E+00 |
|  | **c** | – | – | 4.78E-02 | 7.60E-02 | 4.42E-02 | 5.12E-02 |
| **8** | **a** | – | – | – | 2.08E+03 | – | – |
|  | **b** | – | – | – | -3.15E+00 | – | – |
|  | **c** | – | – | – | 6.63E-02 | – | – |

**Supplementary Material Table 4.** Coefficients for the force-displacement equations during the loading phase at the heel for the LP Vari-flex feet of different categories and sizes with a standard New Balance walking shoe. Equations are in the form of f = ax^2^ + bx + c where f is the force (kN), x is the displacement (m), and a (kN m^-2^), b (kN m^-1^), and c (kN) are coefficients.

| **Heel Force-Displacement Coefficients; Shoe**  (f = ax^2^ + bx + c) | | | | | | | |
| --- | --- | --- | --- | --- | --- | --- | --- |
|  |  | **Size** | | | | | |
| **Category** | **Coefficient** | **24** | **25** | **26** | **27** | **28** | **29** |
| **1** | **a** | 1.59E+03 | 9.70E+02 | 1.03E+03 | – | – | – |
|  | **b** | -1.04E+01 | -3.59E-01 | -6.13E+00 | – | – | – |
|  | **c** | 6.95E-02 | 4.10E-02 | 5.49E-02 | – | – | – |
| **2** | **a** | 1.37E+03 | 1.49E+03 | 1.15E+03 | – | – | – |
|  | **b** | -2.31E+00 | -1.57E+00 | 2.76E-01 | – | – | – |
|  | **c** | 5.57E-02 | 4.74E-02 | 2.19E-02 | – | – | – |
| **3** | **a** | 1.69E+03 | 1.31E+03 | 1.34E+03 | – | 7.63E+02 | 1.27E+03 |
|  | **b** | -5.36E+00 | -2.74E+00 | -5.66E+00 | – | 1.71E+00 | -9.86E+00 |
|  | **c** | 6.55E-02 | 5.58E-02 | 5.63E-02 | – | 4.70E-02 | 7.30E-02 |
| **4** | **a** | 1.91E+03 | 1.51E+03 | 1.49E+03 | 2.39E+03 | 1.40E+03 | 1.46E+03 |
|  | **b** | -1.25E+01 | -1.11E+00 | -8.18E+00 | -1.66E+01 | -4.86E+00 | -1.22E+01 |
|  | **c** | 9.45E-02 | 5.37E-02 | 5.89E-02 | 9.94E-02 | 6.72E-02 | 8.47E-02 |
| **5** | **a** | 2.09E+03 | 1.86E+03 | 2.25E+03 | 2.85E+03 | 1.95E+03 | 2.32E+03 |
|  | **b** | -1.04E+01 | -5.29E+00 | -1.84E+01 | -1.97E+01 | -1.04E+01 | -2.28E+01 |
|  | **c** | 8.97E-02 | 7.37E-02 | 1.02E-01 | 1.08E-01 | 8.90E-02 | 1.30E-01 |
| **6** | **a** | – | 1.69E+03 | 1.60E+03 | 1.88E+03 | 1.63E+03 | 2.16E+03 |
|  | **b** | – | -2.48E-01 | -1.07E+01 | -1.08E+01 | -8.90E+00 | -1.81E+01 |
|  | **c** | – | 7.39E-02 | 9.20E-02 | 1.15E-01 | 9.77E-02 | 1.21E-01 |
| **7** | **a** | – | – | 1.81E+03 | 2.68E+03 | 2.01E+03 | 1.93E+03 |
|  | **b** | – | – | -1.46E+01 | -2.34E+01 | -1.61E+01 | -1.62E+01 |
|  | **c** | – | – | 1.03E-01 | 1.48E-01 | 1.32E-01 | 1.37E-01 |
| **8** | **a** | – | – | – | 2.47E+03 | – | – |
|  | **b** | – | – | – | -2.01E+01 | – | – |
|  | **c** | – | – | – | 1.55E-01 | – | – |

**Supplementary Material Table 5.** Coefficients for the force-displacement equations during the loading phase at the midfoot for the LP Vari-flex feet of different categories and sizes with a standard New Balance walking shoe. Equations are in the form of f = ax^2^ + bx + c where f is the force (kN), x is the displacement (m), and a (kN m^-2^), b (kN m^-1^), and c (kN) are coefficients.

| **Midfoot Force-Displacement Coefficients; Shoe**  (f = ax^2^ + bx + c) | | | | | | | |
| --- | --- | --- | --- | --- | --- | --- | --- |
|  |  | **Size** | | | | | |
| **Category** | **Coefficient** | **24** | **25** | **26** | **27** | **28** | **29** |
| **1** | **a** | 1.25E+04 | 1.15E+04 | 1.02E+04 | – | – | – |
|  | **b** | 4.58E+01 | 1.07E+00 | -8.57E+00 | – | – | – |
|  | **c** | 8.60E-03 | 4.00E-02 | 4.73E-02 | – | – | – |
| **2** | **a** | 1.32E+04 | 1.40E+04 | 9.35E+03 | – | – | – |
|  | **b** | 3.74E+01 | 1.38E+01 | 1.95E+01 | – | – | – |
|  | **c** | 2.23E-02 | 2.25E-02 | 2.89E-02 | – | – | – |
| **3** | **a** | 1.27E+04 | 1.10E+04 | 1.12E+04 | – | 5.24E+03 | 7.21E+03 |
|  | **b** | 3.99E+01 | 2.03E+01 | 3.46E+00 | – | -2.59E+01 | -2.13E+01 |
|  | **c** | 1.17E-02 | 1.56E-02 | 2.33E-02 | – | 7.53E-02 | 7.82E-02 |
| **4** | **a** | 1.16E+04 | 1.39E+04 | 1.07E+04 | 9.60E+03 | 5.89E+03 | 8.56E+03 |
|  | **b** | 5.90E+01 | 1.87E+01 | -2.55E+00 | -1.99E-01 | 4.97E+01 | -1.56E+01 |
|  | **c** | 8.21E-03 | 2.08E-02 | 1.71E-02 | 4.68E-02 | 2.43E-02 | 7.47E-02 |
| **5** | **a** | 1.19E+04 | 1.45E+04 | 1.15E+04 | 1.08E+04 | 8.99E+03 | 9.67E+03 |
|  | **b** | 3.57E+01 | 5.88E+01 | 3.22E+01 | -2.07E+00 | 1.82E+01 | -1.68E+01 |
|  | **c** | 1.22E-02 | 1.13E-02 | 9.49E-03 | 4.64E-02 | 2.75E-02 | 5.96E-02 |
| **6** | **a** | – | 1.15E+04 | 1.08E+04 | 1.21E+04 | 7.21E+03 | 1.19E+04 |
|  | **b** | – | 3.85E+01 | 1.54E+01 | -1.59E+01 | 5.98E+01 | -2.01E+01 |
|  | **c** | – | 1.41E-02 | 2.12E-02 | 6.41E-02 | 5.44E-03 | 7.58E-02 |
| **7** | **a** | – | – | 1.31E+04 | 1.27E+04 | 6.08E+03 | 1.19E+04 |
|  | **b** | – | – | 1.09E+01 | -2.48E+00 | 9.74E+01 | -2.44E+01 |
|  | **c** | – | – | 3.50E-02 | 5.41E-02 | 1.09E-02 | 7.45E-02 |
| **8** | **a** | – | – | – | 1.38E+04 | – | – |
|  | **b** | – | – | – | -1.81E+01 | – | – |
|  | **c** | – | – | – | 7.26E-02 | – | – |

**Supplementary Material Table 6.** Coefficients for the force-displacement equations during the loading phase at the forefoot for the LP Vari-flex feet of different categories and sizes with a standard New Balance walking shoe. Equations are in the form of f = ax^2^ + bx + c where f is the force (kN), x is the displacement (m), and a (kN m^-2^), b (kN m^-1^), and c (kN) are coefficients.

| **Forefoot Force-Displacement Coefficients; Shoe**  (f = ax^2^ + bx + c) | | | | | | | |
| --- | --- | --- | --- | --- | --- | --- | --- |
|  |  | **Size** | | | | | |
| **Category** | **Coefficient** | **24** | **25** | **26** | **27** | **28** | **29** |
| **1** | **a** | 1.42E+03 | 1.39E+03 | 1.26E+03 | – | – | – |
|  | **b** | 8.97E+00 | 3.74E+00 | 2.58E+00 | – | – | – |
|  | **c** | 2.58E-02 | 3.34E-02 | 3.16E-02 | – | – | – |
| **2** | **a** | 1.36E+03 | 1.00E+03 | 1.20E+03 | – | – | – |
|  | **b** | 5.68E+00 | 1.16E+01 | 1.02E+01 | – | – | – |
|  | **c** | 2.91E-02 | 1.42E-02 | 2.08E-02 | – | – | – |
| **3** | **a** | 1.74E+03 | 1.05E+03 | 1.23E+03 | – | 1.28E+03 | 1.07E+03 |
|  | **b** | 7.13E+00 | 1.74E+01 | 7.47E+00 | – | 6.56E+00 | 8.61E+00 |
|  | **c** | 2.70E-02 | 1.95E-02 | 2.61E-02 | – | 1.85E-02 | 2.86E-02 |
| **4** | **a** | 1.59E+03 | 1.78E+03 | 1.39E+03 | 1.24E+03 | 8.48E+02 | 1.13E+03 |
|  | **b** | 5.41E+00 | 4.78E+00 | 6.59E+00 | 1.15E+01 | 1.04E+01 | 1.00E+01 |
|  | **c** | 2.95E-02 | 3.28E-02 | 3.16E-02 | 1.69E-02 | 2.76E-02 | 3.26E-02 |
| **5** | **a** | 1.53E+03 | 1.55E+03 | 1.21E+03 | 1.08E+03 | 9.65E+02 | 1.31E+03 |
|  | **b** | 9.08E+00 | 1.28E+01 | 8.64E+00 | 1.30E+01 | 1.17E+01 | 1.17E+01 |
|  | **c** | 2.96E-02 | 3.35E-02 | 3.00E-02 | 1.81E-02 | 1.68E-02 | 2.69E-02 |
| **6** | **a** | – | 1.65E+03 | 1.72E+03 | 1.18E+03 | 1.29E+03 | 1.66E+03 |
|  | **b** | – | 1.93E+01 | 9.73E+00 | 1.10E+01 | 1.46E+01 | 1.37E+01 |
|  | **c** | – | 2.42E-02 | 4.03E-02 | 2.53E-02 | 2.30E-02 | 2.42E-02 |
| **7** | **a** | – | – | 1.98E+03 | 1.64E+03 | 1.31E+03 | 1.54E+03 |
|  | **b** | – | – | 1.00E+01 | 1.04E+01 | 1.93E+01 | 1.57E+01 |
|  | **c** | – | – | 4.45E-02 | 4.19E-02 | 2.73E-02 | 3.04E-02 |
| **8** | **a** | – | – | – | 1.55E+03 | – | – |
|  | **b** | – | – | – | 1.19E+01 | – | – |
|  | **c** | – | – | – | 3.09E-02 | – | – |
