# Supplementary material for: Low-profile prosthetic foot stiffness category and size, and shoes affect axial and torsional stiffness and hysteresis
Source: Front Rehabil Sci. 2024 Feb 28;5:1290092. doi: 10.3389/fresc.2024.1290092 (PMC10932964; doi:10.3389/fresc.2024.1290092)
Supplement: Supplementary file 6 [file Datasheet6.docx]

Loading Phase

**Supplementary Material Table 7.** Coefficients for the torque-angle equations during the loading phase in plantarflexion (heel) for the LP Vari-flex prosthetic feet of different categories and sizes without a shoe. Equations are in the form of $\tau$ = a$\alpha$^2^ + b$\alpha$ + c where $\tau$ is the torque (kN m), $\alpha$ is the angle (rad), and a (kN m rad^-2^), b (kN m rad^-1^), and c (kN m) are coefficients.

| **Plantarflexion (Heel) Torque-Angle Coefficients; No Shoe**  ($\tau$ = a$\alpha$^2^ + b$\alpha$ + c) | | | | | | | |
| --- | --- | --- | --- | --- | --- | --- | --- |
|  |  | **Size** | | | | | |
| **Category** | **Coefficient** | **24** | **25** | **26** | **27** | **28** | **29** |
| **1** | **a** | 1.42E-01 | 1.36E-01 | 2.12E-01 | – | – | – |
|  | **b** | -4.45E-03 | 1.56E-02 | -6.86E-03 | – | – | – |
|  | **c** | 2.08E-03 | 1.22E-03 | 2.77E-03 | – | – | – |
| **2** | **a** | 1.07E-01 | 2.09E-01 | 1.66E-01 | – | – | – |
|  | **b** | 1.41E-02 | 7.50E-03 | 3.09E-02 | – | – | – |
|  | **c** | 1.66E-03 | 1.53E-03 | 1.22E-04 | – | – | – |
| **3** | **a** | 1.05E-01 | 1.75E-01 | 3.47E-01 | – | 2.06E-01 | 2.86E-01 |
|  | **b** | 2.48E-02 | 1.45E-02 | -1.61E-02 | – | 1.58E-02 | 4.11E-02 |
|  | **c** | 1.57E-03 | 1.47E-03 | 3.07E-03 | – | 1.57E-03 | 1.20E-03 |
| **4** | **a** | 1.87E-01 | 2.01E-01 | 2.83E-01 | 3.19E-01 | 3.00E-01 | 3.43E-01 |
|  | **b** | -1.47E-03 | 2.69E-02 | 6.61E-03 | 2.00E-02 | 1.61E-02 | 5.51E-02 |
|  | **c** | 2.87E-03 | 8.81E-04 | 2.69E-03 | 2.31E-03 | 1.74E-03 | 1.32E-03 |
| **5** | **a** | 1.56E-01 | 2.42E-01 | 4.00E-01 | 3.66E-01 | 5.57E-01 | 6.49E-01 |
|  | **b** | 2.23E-02 | 1.92E-02 | -1.12E-02 | 1.15E-02 | -1.15E-03 | 1.93E-04 |
|  | **c** | 1.96E-03 | 2.67E-03 | 4.00E-03 | 3.27E-03 | 2.69E-03 | 4.39E-03 |
| **6** | **a** | – | 2.40E-01 | 3.12E-01 | 2.92E-01 | 5.33E-01 | 6.46E-01 |
|  | **b** | – | 4.87E-02 | 1.81E-02 | 3.28E-02 | -1.79E-02 | 3.22E-02 |
|  | **c** | – | 1.17E-03 | 2.76E-03 | 1.82E-03 | 4.39E-03 | 3.03E-03 |
| **7** | **a** | – | – | 3.35E-01 | 4.11E-01 | 5.61E-01 | 6.59E-01 |
|  | **b** | – | – | 3.33E-02 | 7.29E-03 | -1.65E-02 | 3.16E-02 |
|  | **c** | – | – | 1.91E-03 | 3.36E-03 | 4.06E-03 | 3.72E-03 |
| **8** | **a** | – | – | – | 4.50E-01 | – | – |
|  | **b** | – | – | – | 1.72E-02 | – | – |
|  | **c** | – | – | – | 3.04E-03 | – | – |

**Supplementary Material Table 8.** Coefficients for the torque-angle equations during the loading phase in plantarflexion (heel) for the LP Vari-flex prosthetic feet of different categories and sizes with a standard New Balance walking shoe. Equations are in the form of $\tau$ = a$\alpha$^2^ + b$\alpha$ + c where $\tau$ is the torque (kN m), $\alpha$ is the angle (rad), and a (kN m rad^-2^), b (kN m rad^-1^), and c (kN m) are coefficients.

| **Plantarflexion (Heel) Torque-Angle Coefficients; Shoe**  ($\tau$ = a$\alpha$^2^ + b$\alpha$ + c) | | | | | | | |
| --- | --- | --- | --- | --- | --- | --- | --- |
|  |  | **Size** | | | | | |
| **Category** | **Coefficient** | **24** | **25** | **26** | **27** | **28** | **29** |
| **1** | **a** | 6.48E-02 | 5.43E-02 | 8.19E-02 | – | – | – |
|  | **b** | -3.91E-03 | 1.12E-02 | 2.79E-03 | – | – | – |
|  | **c** | 2.34E-03 | 1.46E-03 | 1.85E-03 | – | – | – |
| **2** | **a** | 5.43E-02 | 9.49E-02 | 1.00E-01 | – | – | – |
|  | **b** | 6.52E-03 | 6.59E-03 | 8.87E-03 | – | – | – |
|  | **c** | 1.93E-03 | 1.47E-03 | 7.35E-04 | – | – | – |
| **3** | **a** | 6.88E-02 | 6.84E-02 | 1.14E-01 | – | 1.16E-01 | 2.71E-01 |
|  | **b** | 3.96E-03 | 1.05E-02 | 6.16E-04 | – | 1.83E-02 | -3.20E-02 |
|  | **c** | 2.16E-03 | 1.84E-03 | 1.90E-03 | – | 1.98E-03 | 4.23E-03 |
| **4** | **a** | 7.51E-02 | 9.18E-02 | 1.25E-01 | 2.85E-01 | 2.27E-01 | 2.95E-01 |
|  | **b** | -3.74E-03 | 1.02E-02 | -3.33E-03 | -2.75E-02 | -1.47E-03 | -2.87E-02 |
|  | **c** | 3.03E-03 | 1.81E-03 | 1.93E-03 | 4.47E-03 | 3.18E-03 | 4.39E-03 |
| **5** | **a** | 3.69E-02 | 1.10E-01 | 2.01E-01 | 3.29E-01 | 3.28E-01 | 5.35E-01 |
|  | **b** | 1.71E-02 | 8.84E-03 | -2.34E-02 | -3.15E-02 | -2.28E-02 | -7.51E-02 |
|  | **c** | 1.46E-03 | 2.31E-03 | 3.70E-03 | 4.73E-03 | 4.30E-03 | 7.49E-03 |
| **6** | **a** | – | 8.69E-02 | 1.23E-01 | 2.10E-01 | 2.53E-01 | 4.38E-01 |
|  | **b** | – | 2.02E-02 | 2.05E-03 | -7.53E-03 | -6.70E-03 | -4.77E-02 |
|  | **c** | – | 1.84E-03 | 2.54E-03 | 4.94E-03 | 4.38E-03 | 6.32E-03 |
| **7** | **a** | – | – | 1.33E-01 | 3.02E-01 | 3.10E-01 | 3.76E-01 |
|  | **b** | – | – | 1.90E-03 | -3.41E-02 | -2.49E-02 | -3.49E-02 |
|  | **c** | – | – | 2.30E-03 | 6.19E-03 | 5.99E-03 | 6.51E-03 |
| **8** | **a** | – | – | – | 2.86E-01 | – | – |
|  | **b** | – | – | – | -2.55E-02 | – | – |
|  | **c** | – | – | – | 6.42E-03 | – | – |

**Supplementary Material Table 9.** Coefficients for the torque-angle equations during the loading phase in dorsiflexion (forefoot) for the LP Vari-flex prosthetic feet of different categories and sizes without a shoe. Equations are in the form of $\tau$ = a$\alpha$^2^ + b$\alpha$ + c where $\tau$ is the torque (kN m), $\alpha$ is the angle (rad), and a (kN m rad^-2^), b (kN m rad^-1^), and c (kN m) are coefficients.

| **Plantarflexion (Heel) Torque-Angle Coefficients; No Shoe**  ($\tau$ = a$\alpha$^2^ + b$\alpha$ + c) | | | | | | | |
| --- | --- | --- | --- | --- | --- | --- | --- |
|  |  | **Size** | | | | | |
| **Category** | **Coefficient** | **24** | **25** | **26** | **27** | **28** | **29** |
| **1** | **a** | 5.59E+00 | 5.11E+00 | 6.70E+00 | – | – | – |
|  | **b** | -6.51E-03 | -1.10E-01 | -2.88E-03 | – | – | – |
|  | **c** | 2.84E-03 | 5.27E-03 | 2.46E-03 | – | – | – |
| **2** | **a** | 7.32E+00 | 5.81E+00 | 1.01E+01 | – | – | – |
|  | **b** | -1.52E-01 | -3.68E-02 | -2.96E-01 | – | – | – |
|  | **c** | 5.81E-03 | 4.67E-03 | 6.11E-03 | – | – | – |
| **3** | **a** | 9.61E+00 | 4.72E+00 | 7.43E+00 | – | 1.07E+01 | 1.15E+01 |
|  | **b** | -1.13E-01 | 1.65E-01 | -4.67E-02 | – | -1.34E-01 | -2.39E-04 |
|  | **c** | 4.17E-03 | -2.62E-05 | 4.57E-03 | – | 5.80E-03 | 4.03E-03 |
| **4** | **a** | 1.04E+01 | 7.66E+00 | 9.01E+00 | 8.86E+00 | 9.24E+00 | 1.22E+01 |
|  | **b** | -1.78E-01 | -6.33E-02 | -1.53E-01 | -1.72E-01 | -1.63E-02 | 4.45E-02 |
|  | **c** | 6.54E-03 | 6.58E-03 | 5.27E-03 | 6.18E-03 | 6.33E-03 | 3.94E-03 |
| **5** | **a** | 1.11E+01 | 7.39E+00 | 9.03E+00 | 8.63E+00 | 8.54E+00 | 1.19E+01 |
|  | **b** | -1.00E-01 | 1.08E-01 | -3.52E-02 | -1.16E-01 | 1.94E-01 | 1.09E-02 |
|  | **c** | 5.86E-03 | 5.15E-03 | 6.12E-03 | 8.55E-03 | 4.96E-03 | 7.44E-03 |
| **6** | **a** | – | 9.79E+00 | 9.60E+00 | 9.11E+00 | 8.31E+00 | 1.28E+01 |
|  | **b** | – | 1.44E-01 | -2.49E-02 | -2.66E-02 | 1.40E-01 | 5.56E-02 |
|  | **c** | – | 5.31E-03 | 6.89E-03 | 7.35E-03 | 8.18E-03 | 6.98E-03 |
| **7** | **a** | – | – | 1.14E+01 | 1.12E+01 | 1.10E+01 | 1.33E+01 |
|  | **b** | – | – | 1.10E-01 | -1.70E-01 | 2.66E-01 | 1.46E-01 |
|  | **c** | – | – | 6.31E-03 | 1.11E-02 | 4.38E-03 | 8.04E-03 |
| **8** | **a** | – | – | – | 1.30E+01 | – | – |
|  | **b** | – | – | – | -1.70E-01 | – | – |
|  | **c** | – | – | – | 1.06E-02 | – | – |

**Supplementary Material Table 10.** Coefficients for the torque-angle equations during the loading phase in dorsiflexion (forefoot) for the LP Vari-flex prosthetic feet of different categories and sizes with a standard New Balance walking shoe. Equations are in the form of $\tau$ = a$\alpha$^2^ + b$\alpha$ + c where $\tau$ is the torque (kN m), $\alpha$ is the angle (rad), and a (kN m rad^-2^), b (kN m rad^-1^), and c (kN m) are coefficients.

| **Plantarflexion (Heel) Torque-Angle Coefficients; Shoe**  ($\tau$ = a$\alpha$^2^ + b$\alpha$ + c) | | | | | | | |
| --- | --- | --- | --- | --- | --- | --- | --- |
|  |  | **Size** | | | | | |
| **Category** | **Coefficient** | **24** | **25** | **26** | **27** | **28** | **29** |
| **1** | **a** | 5.23E+00 | 5.98E+00 | 6.49E+00 | – | – | – |
|  | **b** | 1.24E-01 | 2.13E-02 | -2.78E-02 | – | – | – |
|  | **c** | 1.22E-03 | 2.69E-03 | 2.95E-03 | – | – | – |
| **2** | **a** | 5.02E+00 | 4.83E+00 | 6.36E+00 | – | – | – |
|  | **b** | 9.70E-02 | 2.00E-01 | 2.00E-01 | – | – | – |
|  | **c** | 2.83E-03 | 3.00E-04 | 4.79E-04 | – | – | – |
| **3** | **a** | 5.88E+00 | 4.70E+00 | 6.34E+00 | – | 9.53E+00 | 9.01E+00 |
|  | **b** | 1.63E-01 | 4.41E-01 | 1.57E-01 | – | 1.25E-01 | 2.34E-01 |
|  | **c** | 9.04E-04 | 8.21E-04 | 1.25E-03 | – | 1.64E-03 | 2.02E-03 |
| **4** | **a** | 5.90E+00 | 7.95E+00 | 7.30E+00 | 8.27E+00 | 6.21E+00 | 9.61E+00 |
|  | **b** | 8.66E-02 | 8.23E-02 | 7.87E-02 | 3.06E-01 | 3.02E-01 | 2.98E-01 |
|  | **c** | 2.97E-03 | 3.77E-03 | 2.40E-03 | 1.88E-03 | 1.24E-03 | 2.46E-03 |
| **5** | **a** | 5.71E+00 | 6.80E+00 | 6.40E+00 | 7.23E+00 | 7.33E+00 | 9.96E+00 |
|  | **b** | 1.73E-01 | 3.07E-01 | 1.78E-01 | 3.17E-01 | 3.75E-01 | 4.79E-01 |
|  | **c** | 2.75E-03 | 2.46E-03 | 2.26E-03 | 1.29E-03 | 1.42E-03 | -1.85E-03 |
| **6** | **a** | – | 7.47E+00 | 8.99E+00 | 7.51E+00 | 9.37E+00 | 1.50E+01 |
|  | **b** | – | 4.78E-01 | 2.00E-01 | 2.96E-01 | 4.73E-01 | 4.49E-01 |
|  | **c** | – | 1.60E-03 | 3.12E-03 | 2.09E-03 | 5.36E-04 | 3.31E-03 |
| **7** | **a** | – | – | 1.04E+01 | 1.04E+01 | 9.86E+00 | 1.33E+01 |
|  | **b** | – | – | 2.44E-01 | 2.71E-01 | 5.96E-01 | 5.32E-01 |
|  | **c** | – | – | 4.82E-03 | 4.89E-03 | 6.54E-04 | 2.34E-03 |
| **8** | **a** | – | – | – | 9.62E+00 | – | – |
|  | **b** | – | – | – | 3.43E-01 | – | – |
|  | **c** | – | – | – | 2.37E-03 | – | – |
