# Supplementary material for: Low-profile prosthetic foot stiffness category and size, and shoes affect axial and torsional stiffness and hysteresis
Source: Front Rehabil Sci. 2024 Feb 28;5:1290092. doi: 10.3389/fresc.2024.1290092 (PMC10932964; doi:10.3389/fresc.2024.1290092)
Supplement: Supplementary file 7 [file Datasheet7.docx]

| **Heel Force-Displacement Coefficients; No Shoe**  (f = ax^2^ + bx + c) | | | | | | | |
| --- | --- | --- | --- | --- | --- | --- | --- |
|  |  | **Size** | | | | | |
| **Category** | **Coefficient** | **24** | **25** | **26** | **27** | **28** | **29** |
| **1** | **a** | 3.14E+03 | 2.10E+03 | 2.01E+03 | – | – | – |
|  | **b** | -1.71E+01 | -4.60E+00 | -7.85E+00 | – | – | – |
|  | **c** | 8.49E-02 | 4.48E-02 | 6.74E-02 | – | – | – |
| **2** | **a** | 2.55E+03 | 3.31E+03 | 1.91E+03 | – | – | – |
|  | **b** | -6.12E+00 | -1.28E+01 | 2.39E+00 | – | – | – |
|  | **c** | 6.77E-02 | 6.87E-02 | 1.40E-02 | – | – | – |
| **3** | **a** | 2.52E+03 | 2.81E+03 | 3.59E+03 | – | 1.28E+03 | 1.43E+03 |
|  | **b** | 1.07E-01 | -8.41E+00 | -2.11E+01 | – | -1.67E+00 | 3.56E+00 |
|  | **c** | 5.29E-02 | 6.22E-02 | 1.04E-01 | – | 4.60E-02 | 4.11E-02 |
| **4** | **a** | 4.07E+03 | 3.25E+03 | 2.89E+03 | 2.52E+03 | 1.79E+03 | 1.66E+03 |
|  | **b** | -1.99E+01 | -3.45E+00 | -7.45E+00 | -2.83E+00 | -1.47E+00 | 6.36E+00 |
|  | **c** | 1.06E-01 | 5.09E-02 | 7.13E-02 | 5.77E-02 | 5.42E-02 | 3.90E-02 |
| **5** | **a** | 3.65E+03 | 3.73E+03 | 3.95E+03 | 2.83E+03 | 3.18E+03 | 2.95E+03 |
|  | **b** | -4.91E+00 | -7.06E+00 | -1.72E+01 | -7.62E+00 | -8.71E+00 | -1.07E+01 |
|  | **c** | 7.61E-02 | 8.36E-02 | 1.03E-01 | 7.91E-02 | 7.14E-02 | 9.74E-02 |
| **6** | **a** | – | 3.80E+03 | 3.20E+03 | 2.41E+03 | 3.06E+03 | 3.00E+03 |
|  | **b** | – | 5.72E+00 | -4.70E+00 | -8.88E-02 | -1.57E+01 | -2.09E+00 |
|  | **c** | – | 4.74E-02 | 7.47E-02 | 5.53E-02 | 1.05E-01 | 7.59E-02 |
| **7** | **a** | – | – | 3.58E+03 | 3.31E+03 | 3.30E+03 | 2.96E+03 |
|  | **b** | – | – | -1.35E+00 | -1.38E+01 | -1.37E+01 | -1.04E+00 |
|  | **c** | – | – | 6.24E-02 | 9.74E-02 | 1.04E-01 | 8.05E-02 |
| **8** | **a** | – | – | – | 3.59E+03 | – | – |
|  | **b** | – | – | – | -8.85E+00 | – | – |
|  | **c** | – | – | – | 8.71E-02 | – | – |

Unloading Phase

**Supplementary Material Table 11.** Coefficients for the force-displacement equations during the unloading phase at the heel for the LP Vari-flex prosthetic feet of different categories and sizes without a shoe. Equations are in the form of f = ax^2^ + bx + c where f is the force (kN), x is the displacement (m), and a (kN m^-2^), b (kN m^-1^), and c (kN) are coefficients.

**Supplementary Material Table 12.** Coefficients for the force-displacement equations during the unloading phase at the midfoot for the LP Vari-flex feet of different categories and sizes without a shoe. Equations are in the form of f = ax^2^ + bx + c where f is the force (kN), x is the displacement (m), and a (kN m^-2^), b (kN m^-1^), and c (kN) are coefficients.

| **Midfoot Force-Displacement Coefficients; No Shoe**  (ax^2^ + bx + c = f) | | | | | | | |
| --- | --- | --- | --- | --- | --- | --- | --- |
|  |  | **Size** | | | | | |
| **Category** | **Coefficient** | **24** | **25** | **26** | **27** | **28** | **29** |
| **1** | **a** | 4.75E+04 | 5.53E+04 | 3.44E+04 | – | – | – |
|  | **b** | 4.13E+01 | -6.67E+01 | -1.04E+02 | – | – | – |
|  | **c** | -3.90E-03 | 4.73E-02 | 1.30E-01 | – | – | – |
| **2** | **a** | 6.66E+04 | 7.17E+04 | 4.39E+04 | – | – | – |
|  | **b** | -5.08E+01 | -5.73E+01 | -1.42E+02 | – | – | – |
|  | **c** | 3.79E-02 | 5.33E-02 | 1.64E-01 | – | – | – |
| **3** | **a** | 7.41E+04 | 4.50E+04 | 5.59E+04 | – | 2.05E+04 | 2.10E+04 |
|  | **b** | 8.68E+00 | -2.56E+01 | -1.28E+02 | – | -5.89E+01 | -8.57E+01 |
|  | **c** | 1.57E-02 | 3.82E-02 | 1.18E-01 | – | 8.97E-02 | 1.33E-01 |
| **4** | **a** | 7.17E+04 | 6.23E+04 | 4.87E+04 | 3.20E+04 | 2.01E+04 | 2.03E+04 |
|  | **b** | 2.77E+01 | -4.66E+01 | -1.32E+02 | -9.59E+01 | -2.53E+01 | -8.13E+01 |
|  | **c** | -1.10E-02 | 5.26E-02 | 1.26E-01 | 1.19E-01 | 6.07E-02 | 1.32E-01 |
| **5** | **a** | 7.00E+04 | 7.51E+04 | 5.04E+04 | 2.74E+04 | 3.47E+04 | 2.52E+04 |
|  | **b** | 2.26E+01 | -3.14E+01 | -6.23E+01 | -6.72E+01 | -5.04E+01 | -4.06E+01 |
|  | **c** | 6.78E-03 | 1.18E-02 | 4.34E-02 | 7.44E-02 | 6.93E-02 | 4.70E-02 |
| **6** | **a** | – | 6.33E+04 | 4.86E+04 | 2.87E+04 | 2.88E+04 | 2.85E+04 |
|  | **b** | – | -6.45E+01 | -4.55E+01 | -7.11E+01 | 1.78E+01 | -1.61E+01 |
|  | **c** | – | 8.11E-02 | 3.51E-02 | 8.19E-02 | -1.62E-02 | 2.73E-02 |
| **7** | **a** | – | – | 5.61E+04 | 3.21E+04 | 2.24E+04 | 2.36E+04 |
|  | **b** | – | – | -1.32E+02 | -2.14E+01 | 9.14E+01 | 1.21E+01 |
|  | **c** | – | – | 1.17E-01 | 4.19E-02 | -8.25E-02 | -8.29E-02 |
| **8** | **a** | – | – | – | 3.55E+04 | – | – |
|  | **b** | – | – | – | -4.34E-01 | – | – |
|  | **c** | – | – | – | 2.24E-02 | – | – |

**Supplementary Material Table 13.** Coefficients for the force-displacement equations during the unloading phase at the forefoot for the LP Vari-flex feet of different categories and sizes without a shoe. Equations are in the form of f = ax^2^ + bx + c where f is the force (kN), x is the displacement (m), and a (kN m^-2^), b (kN m^-1^), and c (kN) are coefficients.

| **Forefoot Force-Displacement Coefficients; No Shoe**  (ax^2^ + bx + c = f) | | | | | | | |
| --- | --- | --- | --- | --- | --- | --- | --- |
|  | | **Size** | | | | | |
| **Category** | **Coefficient** | **24** | **25** | **26** | **27** | **28** | **29** |
| **1** | **a** | 1.92E+03 | 1.37E+03 | 1.94E+03 | – | – | – |
|  | **b** | -7.77E+00 | -8.17E+00 | -1.36E+01 | – | – | – |
|  | **c** | 5.02E-02 | 6.48E-02 | 7.65E-02 | – | – | – |
| **2** | **a** | 2.49E+03 | 1.44E+03 | 2.53E+03 | – | – | – |
|  | **b** | -1.51E+01 | -3.44E+00 | -2.01E+01 | – | – | – |
|  | **c** | 7.56E-02 | 3.34E-02 | 9.31E-02 | – | – | – |
| **3** | **a** | 3.35E+03 | 1.40E+03 | 2.02E+03 | – | 1.70E+03 | 1.64E+03 |
|  | **b** | -1.78E+01 | 1.43E+00 | -1.48E+01 | – | -8.62E+00 | -4.50E+00 |
|  | **c** | 7.19E-02 | 3.37E-02 | 8.66E-02 | – | 6.34E-02 | 5.02E-02 |
| **4** | **a** | 3.60E+03 | 2.11E+03 | 2.29E+03 | 1.85E+03 | 1.59E+03 | 1.73E+03 |
|  | **b** | -2.25E+01 | -9.69E+00 | -1.39E+01 | -1.37E+01 | -8.32E+00 | -1.98E+00 |
|  | **c** | 9.41E-02 | 7.76E-02 | 8.50E-02 | 8.52E-02 | 7.41E-02 | 5.02E-02 |
| **5** | **a** | 3.59E+03 | 2.04E+03 | 2.08E+03 | 1.72E+03 | 1.47E+03 | 1.70E+03 |
|  | **b** | -1.50E+01 | -4.19E+00 | -9.69E+00 | -1.19E+01 | -1.44E+00 | -6.75E+00 |
|  | **c** | 7.76E-02 | 6.42E-02 | 7.44E-02 | 9.06E-02 | 5.62E-02 | 7.15E-02 |
| **6** | **a** | – | 2.67E+03 | 2.16E+03 | 1.86E+03 | 1.41E+03 | 1.85E+03 |
|  | **b** | – | -4.53E+00 | -8.78E+00 | -1.04E+01 | -3.78E+00 | -7.13E+00 |
|  | **c** | – | 6.42E-02 | 7.61E-02 | 8.32E-02 | 7.07E-02 | 7.41E-02 |
| **7** | **a** | – | – | 2.60E+03 | 2.16E+03 | 1.80E+03 | 1.90E+03 |
|  | **b** | – | – | -6.89E+00 | -1.36E+01 | 9.94E-01 | -4.86E+00 |
|  | **c** | – | – | 7.61E-02 | 1.03E-01 | 5.51E-02 | 7.47E-02 |
| **8** | **a** | – | – | – | 2.45E+03 | – | – |
|  | **b** | – | – | – | -1.50E+01 | – | – |
|  | **c** | – | – | – | 1.03E-01 | – | – |

**Supplementary Material Table 14.** Coefficients for the force-displacement equations during the unloading phase at the heel for the LP Vari-flex feet of different categories and sizes with a standard New Balance walking shoe. Equations are in the form of f = ax^2^ + bx + c where f is the force (kN), x is the displacement (m), and a (kN m^-2^), b (kN m^-1^), and c (kN) are coefficients.

| **Heel Force-Displacement Coefficients; Shoe**  (f = ax^2^ + bx + c) | | | | | | | |
| --- | --- | --- | --- | --- | --- | --- | --- |
|  |  | **Size** | | | | | |
| **Category** | **Coefficient** | **24** | **25** | **26** | **27** | **28** | **29** |
| **1** | **a** | 2.62E+03 | 1.55E+03 | 1.48E+03 | – | – | – |
|  | **b** | -4.54E+01 | -1.99E+01 | -2.34E+01 | – | – | – |
|  | **c** | 2.45E-01 | 1.19E-01 | 1.47E-01 | – | – | – |
| **2** | **a** | 2.39E+03 | 2.64E+03 | 1.92E+03 | – | – | – |
|  | **b** | -3.16E+01 | -3.30E+01 | -2.62E+01 | – | – | – |
|  | **c** | 1.65E-01 | 1.63E-01 | 1.39E-01 | – | – | – |
| **3** | **a** | 2.84E+03 | 2.17E+03 | 2.12E+03 | – | 1.09E+03 | 1.90E+03 |
|  | **b** | -3.92E+01 | -3.03E+01 | -3.19E+01 | – | -1.12E+01 | -3.38E+01 |
|  | **c** | 1.99E-01 | 1.68E-01 | 1.78E-01 | – | 9.10E-02 | 2.07E-01 |
| **4** | **a** | 3.20E+03 | 2.41E+03 | 2.30E+03 | 2.65E+03 | 2.02E+03 | 2.14E+03 |
|  | **b** | -5.36E+01 | -2.95E+01 | -3.70E+01 | -3.14E+01 | -2.58E+01 | -3.73E+01 |
|  | **c** | 2.89E-01 | 1.57E-01 | 2.11E-01 | 1.52E-01 | 1.53E-01 | 2.30E-01 |
| **5** | **a** | 3.21E+03 | 2.87E+03 | 3.33E+03 | 3.97E+03 | 2.79E+03 | 3.21E+03 |
|  | **b** | -4.54E+01 | -3.60E+01 | -5.37E+01 | -5.37E+01 | -3.83E+01 | -5.35E+01 |
|  | **c** | 2.29E-01 | 1.85E-01 | 2.75E-01 | 2.49E-01 | 2.04E-01 | 2.95E-01 |
| **6** | **a** | – | 2.47E+03 | 2.25E+03 | 2.61E+03 | 2.28E+03 | 2.98E+03 |
|  | **b** | – | -2.48E+01 | -3.57E+01 | -3.64E+01 | -3.30E+01 | -4.74E+01 |
|  | **c** | – | 1.34E-01 | 2.11E-01 | 2.19E-01 | 2.07E-01 | 2.69E-01 |
| **7** | **a** | – | – | 2.45E+03 | 3.56E+03 | 2.72E+03 | 2.55E+03 |
|  | **b** | – | – | -4.00E+01 | -5.50E+01 | -4.27E+01 | -4.09E+01 |
|  | **c** | – | – | 2.31E-01 | 2.93E-01 | 2.58E-01 | 2.54E-01 |
| **8** | **a** | – | – | – | 3.37E+03 | – | – |
|  | **b** | – | – | – | -5.36E+01 | – | – |
|  | **c** | – | – | – | 3.11E-01 | – | – |

**Supplementary Material Table 15.** Coefficients for the force-displacement equations during the unloading phase at the midfoot for the LP Vari-flex feet of different categories and sizes with a standard New Balance walking shoe. Equations are in the form of f = ax^2^ + bx + c where f is the force (kN), x is the displacement (m), and a (kN m^-2^), b (kN m^-1^), and c (kN) are coefficients.

| **Midfoot Force-Displacement Coefficients; Shoe**  (f = ax^2^ + bx + c) | | | | | | | |
| --- | --- | --- | --- | --- | --- | --- | --- |
|  |  | **Size** | | | | | |
| **Category** | **Coefficient** | **24** | **25** | **26** | **27** | **28** | **29** |
| **1** | **a** | 2.35E+04 | 1.94E+04 | 1.82E+04 | – | – | – |
|  | **b** | -2.59E+01 | -6.95E+01 | -9.36E+01 | – | – | – |
|  | **c** | 4.01E-02 | 1.11E-01 | 1.64E-01 | – | – | – |
| **2** | **a** | 2.64E+04 | 2.51E+04 | 1.75E+04 | – | – | – |
|  | **b** | -6.10E+01 | -7.66E+01 | -5.75E+01 | – | – | – |
|  | **c** | 8.58E-02 | 1.06E-01 | 1.02E-01 | – | – | – |
| **3** | **a** | 2.62E+04 | 1.88E+04 | 1.97E+04 | – | 7.24E+03 | 1.22E+04 |
|  | **b** | -7.32E+01 | -5.52E+01 | -8.74E+01 | – | -6.25E+01 | -9.49E+01 |
|  | **c** | 1.00E-01 | 8.81E-02 | 1.50E-01 | – | 1.80E-01 | 2.38E-01 |
| **4** | **a** | 2.53E+04 | 2.45E+04 | 1.82E+04 | 1.70E+04 | 1.07E+04 | 1.46E+04 |
|  | **b** | -5.35E+01 | -8.04E+01 | -9.44E+01 | -9.38E+01 | 2.63E-01 | -1.01E+02 |
|  | **c** | 8.09E-02 | 1.24E-01 | 1.70E-01 | 1.82E-01 | 3.99E-02 | 2.38E-01 |
| **5** | **a** | 2.27E+04 | 2.70E+04 | 2.11E+04 | 1.91E+04 | 1.48E+04 | 1.60E+04 |
|  | **b** | -7.59E+01 | -4.27E+01 | -6.64E+01 | -1.08E+02 | -5.28E+01 | -1.06E+02 |
|  | **c** | 1.17E-01 | 6.32E-02 | 1.08E-01 | 2.10E-01 | 1.13E-01 | 2.33E-01 |
| **6** | **a** | – | 2.27E+04 | 1.88E+04 | 2.08E+04 | 1.31E+04 | 1.96E+04 |
|  | **b** | – | -8.29E+01 | -8.56E+01 | -1.34E+02 | -5.24E+00 | -1.23E+02 |
|  | **c** | – | 1.50E-01 | 1.61E-01 | 2.78E-01 | 3.62E-02 | 2.58E-01 |
| **7** | **a** | – | – | 2.14E+04 | 2.16E+04 | 1.32E+04 | 1.85E+04 |
|  | **b** | – | – | -9.50E+01 | -1.26E+02 | 2.52E+01 | -1.23E+02 |
|  | **c** | – | – | 1.78E-01 | 2.61E-01 | 1.08E-02 | 2.66E-01 |
| **8** | **a** | – | – | – | 2.25E+04 | – | – |
|  | **b** | – | – | – | -1.43E+02 | – | – |
|  | **c** | – | – | – | 3.00E-01 | – | – |

**Supplementary Material Table 16.** Coefficients for the force-displacement equations during the unloading phase at the forefoot for the LP Vari-flex feet of different categories and sizes with a standard New Balance walking shoe. Equations are in the form of f = ax^2^ + bx + c where f is the force (kN), x is the displacement (m), and a (kN m^-2^), b (kN m^-1^), and c (kN) are coefficients.

| **Forefoot Force-Displacement Coefficients; Shoe**  (f = ax^2^ + bx + c) | | | | | | | |
| --- | --- | --- | --- | --- | --- | --- | --- |
|  |  | **Size** | | | | | |
| **Category** | **Coefficient** | **24** | **25** | **26** | **27** | **28** | **29** |
| **1** | **a** | 2.13E+03 | 2.07E+03 | 1.81E+03 | – | – | – |
|  | **b** | -6.00E+00 | -1.29E+01 | -1.29E+01 | – | – | – |
|  | **c** | 4.92E-02 | 7.18E-02 | 7.39E-02 | – | – | – |
| **2** | **a** | 1.97E+03 | 1.62E+03 | 1.95E+03 | – | – | – |
|  | **b** | -8.79E+00 | -2.73E+00 | -7.35E+00 | – | – | – |
|  | **c** | 6.04E-02 | 3.69E-02 | 5.13E-02 | – | – | – |
| **3** | **a** | 2.60E+03 | 1.82E+03 | 1.86E+03 | – | 1.81E+03 | 1.67E+03 |
|  | **b** | -1.24E+01 | 5.97E-01 | -9.03E+00 | – | -7.11E+00 | -6.85E+00 |
|  | **c** | 7.15E-02 | 3.90E-02 | 6.29E-02 | – | 4.94E-02 | 5.97E-02 |
| **4** | **a** | 2.34E+03 | 2.51E+03 | 2.02E+03 | 2.16E+03 | 1.22E+03 | 1.78E+03 |
|  | **b** | -1.36E+01 | -1.18E+01 | -1.02E+01 | -1.11E+01 | -3.59E-01 | -6.62E+00 |
|  | **c** | 8.01E-02 | 6.97E-02 | 6.92E-02 | 6.46E-02 | 4.35E-02 | 6.21E-02 |
| **5** | **a** | 2.22E+03 | 2.29E+03 | 1.74E+03 | 1.73E+03 | 1.51E+03 | 2.06E+03 |
|  | **b** | -9.42E+00 | -4.41E+00 | -7.18E+00 | -5.40E+00 | -3.31E+00 | -8.10E+00 |
|  | **c** | 6.85E-02 | 5.96E-02 | 6.64E-02 | 5.17E-02 | 4.71E-02 | 6.55E-02 |
| **6** | **a** | – | 2.54E+03 | 2.40E+03 | 1.77E+03 | 1.86E+03 | 2.54E+03 |
|  | **b** | – | -8.36E-01 | -7.96E+00 | -7.02E+00 | -7.92E-01 | -8.17E+00 |
|  | **c** | – | 5.08E-02 | 7.32E-02 | 6.20E-02 | 5.05E-02 | 6.12E-02 |
| **7** | **a** | – | – | 2.73E+03 | 2.71E+03 | 1.87E+03 | 2.32E+03 |
|  | **b** | – | – | -1.01E+01 | -1.93E+01 | 3.65E+00 | -5.25E+00 |
|  | **c** | – | – | 8.38E-02 | 1.03E-01 | 4.38E-02 | 6.23E-02 |
| **8** | **a** | – | – | – | 2.26E+03 | – | – |
|  | **b** | – | – | – | -1.01E+01 | – | – |
|  | **c** | – | – | – | 7.68E-02 | – | – |
