# Supplementary material for: Low-profile prosthetic foot stiffness category and size, and shoes affect axial and torsional stiffness and hysteresis
Source: Front Rehabil Sci. 2024 Feb 28;5:1290092. doi: 10.3389/fresc.2024.1290092 (PMC10932964; doi:10.3389/fresc.2024.1290092)
Supplement: Supplementary file 8 [file Datasheet8.docx]

Unloading Phase

**Supplementary Material Table 17.** Coefficients for the torque-angle equations during the unloading phase in plantarflexion (heel) for the LP Vari-flex prosthetic feet of different categories and sizes without a shoe. Equations are in the form of $\tau$ = a$\alpha$^2^ + b$\alpha$ + c where $\tau$ is the torque (kN m), $\alpha$ is the angle (rad), and a (kN m rad^-2^), b (kN m rad^-1^), and c (kN m) are coefficients.

| **Plantarflexion (Heel) Torque-Angle Coefficients; No Shoe**  ($\tau$ = a$\alpha$^2^ + b$\alpha$ + c) | | | | | | | |
| --- | --- | --- | --- | --- | --- | --- | --- |
|  |  | **Size** | | | | | |
| **Category** | **Coefficient** | **24** | **25** | **26** | **27** | **28** | **29** |
| **1** | **a** | 1.77E-01 | 1.71E-01 | 2.17E-01 | – | – | – |
|  | **b** | -2.44E-02 | -2.60E-03 | -1.36E-02 | – | – | – |
|  | **c** | 3.53E-03 | 1.98E-03 | 3.04E-03 | – | – | – |
| **2** | **a** | 1.37E-01 | 2.54E-01 | 1.78E-01 | – | – | – |
|  | **b** | -2.18E-03 | -1.29E-02 | 2.48E-02 | – | – | – |
|  | **c** | 2.48E-03 | 2.47E-03 | -2.07E-04 | – | – | – |
| **3** | **a** | 1.34E-01 | 2.11E-01 | 3.96E-01 | – | 2.25E-01 | 3.36E-01 |
|  | **b** | 8.75E-03 | -4.96E-03 | -4.27E-02 | – | 6.32E-03 | 1.94E-02 |
|  | **c** | 2.05E-03 | 2.21E-03 | 4.64E-03 | – | 1.86E-03 | 1.94E-03 |
| **4** | **a** | 2.28E-01 | 2.52E-01 | 3.15E-01 | 3.68E-01 | 3.31E-01 | 3.95E-01 |
|  | **b** | -2.52E-02 | 1.14E-03 | -1.13E-02 | -2.74E-04 | -1.88E-04 | 3.08E-02 |
|  | **c** | 4.10E-03 | 1.84E-03 | 3.23E-03 | 2.71E-03 | 2.65E-03 | 1.81E-03 |
| **5** | **a** | 1.97E-01 | 2.89E-01 | 4.40E-01 | 4.10E-01 | 6.25E-01 | 7.24E-01 |
|  | **b** | -1.18E-05 | -5.03E-03 | -3.14E-02 | -1.23E-02 | -3.33E-02 | -3.13E-02 |
|  | **c** | 2.81E-03 | 3.22E-03 | 4.63E-03 | 3.75E-03 | 4.17E-03 | 5.53E-03 |
| **6** | **a** | – | 3.01E-01 | 3.41E-01 | 3.37E-01 | 5.72E-01 | 7.27E-01 |
|  | **b** | – | 1.99E-02 | 1.98E-03 | 9.74E-03 | -3.84E-02 | -3.65E-04 |
|  | **c** | – | 1.87E-03 | 3.19E-03 | 2.22E-03 | 5.17E-03 | 3.93E-03 |
| **7** | **a** | – | – | 3.77E-01 | 4.65E-01 | 6.14E-01 | 7.35E-01 |
|  | **b** | – | – | 1.02E-02 | -2.32E-02 | -4.38E-02 | 1.57E-03 |
|  | **c** | – | – | 2.31E-03 | 4.24E-03 | 5.50E-03 | 4.66E-03 |
| **8** | **a** | – | – | – | 5.06E-01 | – | – |
|  | **b** | – | – | – | -1.20E-02 | – | – |
|  | **c** | – | – | – | 3.79E-03 | – | – |

**Supplementary Material Table 18.** Coefficients for the torque-angle equations during the unloading phase in plantarflexion (heel) for the LP Vari-flex prosthetic feet of different categories and sizes with a standard New Balance walking shoe. Equations are in the form of $\tau$ = a$\alpha$^2^ + b$\alpha$ + c where $\tau$ is the torque (kN m), $\alpha$ is the angle (rad), and a (kN m rad^-2^), b (kN m rad^-1^), and c (kN m) are coefficients.

| **Plantarflexion (Heel) Torque-Angle Coefficients; Shoe**  ($\tau$ = a$\alpha$^2^ + b$\alpha$ + c) | | | | | | | |
| --- | --- | --- | --- | --- | --- | --- | --- |
|  |  | **Size** | | | | | |
| **Category** | **Coefficient** | **24** | **25** | **26** | **27** | **28** | **29** |
| **1** | **a** | 1.13E-01 | 9.03E-02 | 1.17E-01 | – | – | – |
|  | **b** | -4.69E-02 | -1.65E-02 | -2.76E-02 | – | – | – |
|  | **c** | 7.39E-03 | 3.12E-03 | 4.17E-03 | – | – | – |
| **2** | **a** | 9.52E-02 | 1.68E-01 | 1.63E-01 | – | – | – |
|  | **b** | -2.53E-02 | -4.14E-02 | -3.58E-02 | – | – | – |
|  | **c** | 4.31E-03 | 5.06E-03 | 4.18E-03 | – | – | – |
| **3** | **a** | 1.11E-01 | 1.11E-01 | 1.80E-01 | – | 1.67E-01 | 3.90E-01 |
|  | **b** | -2.96E-02 | -2.21E-02 | -4.78E-02 | – | -1.77E-02 | -1.06E-01 |
|  | **c** | 4.56E-03 | 3.57E-03 | 5.99E-03 | – | 3.43E-03 | 1.08E-02 |
| **4** | **a** | 1.19E-01 | 1.46E-01 | 1.92E-01 | 3.31E-01 | 3.29E-01 | 4.16E-01 |
|  | **b** | -4.29E-02 | -3.03E-02 | -5.48E-02 | -7.02E-02 | -6.22E-02 | -1.02E-01 |
|  | **c** | 6.75E-03 | 4.26E-03 | 6.94E-03 | 6.96E-03 | 7.01E-03 | 1.00E-02 |
| **5** | **a** | 5.83E-02 | 1.70E-01 | 2.97E-01 | 4.57E-01 | 4.74E-01 | 7.17E-01 |
|  | **b** | -4.32E-03 | -3.58E-02 | -9.15E-02 | -1.09E-01 | -1.07E-01 | -1.75E-01 |
|  | **c** | 1.70E-03 | 4.88E-03 | 1.02E-02 | 1.04E-02 | 1.04E-02 | 1.50E-02 |
| **6** | **a** | – | 1.30E-01 | 1.75E-01 | 2.92E-01 | 3.47E-01 | 5.87E-01 |
|  | **b** | – | -1.26E-02 | -4.13E-02 | -6.47E-02 | -6.80E-02 | -1.36E-01 |
|  | **c** | – | 2.46E-03 | 5.65E-03 | 8.60E-03 | 7.88E-03 | 1.26E-02 |
| **7** | **a** | – | – | 1.82E-01 | 4.08E-01 | 4.16E-01 | 4.87E-01 |
|  | **b** | – | – | -4.17E-02 | -1.13E-01 | -9.72E-02 | -1.07E-01 |
|  | **c** | – | – | 5.25E-03 | 1.27E-02 | 1.11E-02 | 1.12E-02 |
| **8** | **a** | – | – | – | 3.92E-01 | – | – |
|  | **b** | – | – | – | -1.05E-01 | – | – |
|  | **c** | – | – | – | 1.26E-02 | – | – |

**Supplementary Material Table 19.** Coefficients for the torque-angle equations during the unloading phase in dorsiflexion (forefoot) for the LP Vari-flex prosthetic feet of different categories and sizes without a shoe. Equations are in the form of $\tau$ = a$\alpha$^2^ + b$\alpha$ + c where $\tau$ is the torque (kN m), $\alpha$ is the angle (rad), and a (kN m rad^-2^), b (kN m rad^-1^), and c (kN m) are coefficients.

| **Plantarflexion (Heel) Torque-Angle Coefficients; No Shoe**  ($\tau$ = a$\alpha$^2^ + b$\alpha$ + c) | | | | | | | |
| --- | --- | --- | --- | --- | --- | --- | --- |
|  |  | **Size** | | | | | |
| **Category** | **Coefficient** | **24** | **25** | **26** | **27** | **28** | **29** |
| **1** | **a** | 6.82E+00 | 5.29E+00 | 7.44E+00 | – | – | – |
|  | **b** | -2.01E-01 | -1.43E-01 | -1.33E-01 | – | – | – |
|  | **c** | 6.06E-03 | 6.41E-03 | 4.46E-03 | – | – | – |
| **2** | **a** | 8.73E+00 | 4.88E+00 | 1.25E+01 | – | – | – |
|  | **b** | -3.62E-01 | 9.72E-02 | -7.14E-01 | – | – | – |
|  | **c** | 9.42E-03 | -2.35E-03 | 1.62E-02 | – | – | – |
| **3** | **a** | 1.19E+01 | 6.00E+00 | 8.95E+00 | – | 1.11E+01 | 1.34E+01 |
|  | **b** | -4.38E-01 | -4.46E-02 | -3.15E-01 | – | -2.28E-01 | -2.59E-01 |
|  | **c** | 8.99E-03 | 2.65E-03 | 8.86E-03 | – | 5.20E-03 | 8.04E-03 |
| **4** | **a** | 1.26E+01 | 9.04E+00 | 1.15E+01 | 1.08E+01 | 1.10E+01 | 1.43E+01 |
|  | **b** | -4.98E-01 | -2.85E-01 | -5.43E-01 | -5.18E-01 | -2.87E-01 | -2.54E-01 |
|  | **c** | 1.13E-02 | 1.03E-02 | 1.44E-02 | 1.37E-02 | 9.83E-03 | 8.32E-03 |
| **5** | **a** | 1.30E+01 | 8.87E+00 | 1.08E+01 | 1.04E+01 | 1.04E+01 | 1.37E+01 |
|  | **b** | -3.76E-01 | -1.36E-01 | -3.14E-01 | -4.16E-01 | -8.19E-02 | -2.61E-01 |
|  | **c** | 9.76E-03 | 8.01E-03 | 1.07E-02 | 1.39E-02 | 7.27E-03 | 1.02E-02 |
| **6** | **a** | – | 1.18E+01 | 1.11E+01 | 1.07E+01 | 9.94E+00 | 1.52E+01 |
|  | **b** | – | -1.47E-01 | -2.75E-01 | -3.11E-01 | -1.25E-01 | -3.01E-01 |
|  | **c** | – | 8.63E-03 | 1.02E-02 | 1.13E-02 | 9.77E-03 | 1.12E-02 |
| **7** | **a** | – | – | 1.35E+01 | 1.32E+01 | 1.29E+01 | 1.57E+01 |
|  | **b** | – | – | -2.28E-01 | -5.21E-01 | -4.89E-02 | -2.13E-01 |
|  | **c** | – | – | 1.02E-02 | 1.70E-02 | 7.01E-03 | 1.13E-02 |
| **8** | **a** | – | – | – | 1.50E+01 | – | – |
|  | **b** | – | – | – | -5.19E-01 | – | – |
|  | **c** | – | – | – | 1.58E-02 | – | – |

**Supplementary Material Table 20.** Coefficients for the torque-angle equations during the unloading phase in dorsiflexion (forefoot) for the LP Vari-flex prosthetic feet of different categories and sizes with a standard New Balance walking shoe. Equations are in the form of $\tau$ = a$\alpha$^2^ + b$\alpha$ + c where $\tau$ is the torque (kN m), $\alpha$ is the angle (rad), and a (kN m rad^-2^), b (kN m rad^-1^), and c (kN m) are coefficients.

| **Plantarflexion (Heel) Torque-Angle Coefficients; Shoe**  ($\tau$ = a$\alpha$^2^ + b$\alpha$ + c) | | | | | | | |
| --- | --- | --- | --- | --- | --- | --- | --- |
|  |  | **Size** | | | | | |
| **Category** | **Coefficient** | **24** | **25** | **26** | **27** | **28** | **29** |
| **1** | **a** | 7.40E+00 | 8.26E+00 | 8.89E+00 | – | – | – |
|  | **b** | -1.96E-01 | -3.33E-01 | -4.21E-01 | – | – | – |
|  | **c** | 6.30E-03 | 8.48E-03 | 1.03E-02 | – | – | – |
| **2** | **a** | 6.62E+00 | 7.48E+00 | 9.50E+00 | – | – | – |
|  | **b** | -1.99E-01 | -2.19E-01 | -2.41E-01 | – | – | – |
|  | **c** | 6.35E-03 | 6.17E-03 | 6.00E-03 | – | – | – |
| **3** | **a** | 8.31E+00 | 7.77E+00 | 9.03E+00 | – | 1.21E+01 | 1.32E+01 |
|  | **b** | -2.15E-01 | 2.43E-02 | -2.68E-01 | – | -2.60E-01 | -3.50E-01 |
|  | **c** | 6.35E-03 | 3.44E-03 | 7.40E-03 | – | 4.84E-03 | 9.15E-03 |
| **4** | **a** | 8.30E+00 | 1.09E+01 | 1.01E+01 | 1.35E+01 | 8.59E+00 | 1.45E+01 |
|  | **b** | -3.09E-01 | -3.63E-01 | -3.50E-01 | -4.01E-01 | -7.25E-02 | -3.68E-01 |
|  | **c** | 9.35E-03 | 9.56E-03 | 9.35E-03 | 1.00E-02 | 5.27E-03 | 1.03E-02 |
| **5** | **a** | 8.06E+00 | 9.80E+00 | 8.88E+00 | 1.09E+01 | 1.06E+01 | 1.54E+01 |
|  | **b** | -2.36E-01 | -1.50E-01 | -2.37E-01 | -2.41E-01 | -1.12E-01 | -2.75E-01 |
|  | **c** | 8.41E-03 | 6.90E-03 | 8.35E-03 | 7.58E-03 | 5.25E-03 | 7.41E-03 |
| **6** | **a** | – | 1.13E+01 | 1.21E+01 | 1.09E+01 | 1.31E+01 | 2.12E+01 |
|  | **b** | – | -8.43E-02 | -3.02E-01 | -2.61E-01 | -7.62E-02 | -3.13E-01 |
|  | **c** | – | 6.11E-03 | 9.41E-03 | 8.76E-03 | 5.98E-03 | 8.02E-03 |
| **7** | **a** | – | – | 1.39E+01 | 1.59E+01 | 1.35E+01 | 1.91E+01 |
|  | **b** | – | – | -3.06E-01 | -5.43E-01 | 3.25E-02 | -2.56E-01 |
|  | **c** | – | – | 1.07E-02 | 1.14E-02 | 5.43E-03 | 8.47E-03 |
| **8** | **a** | – | – | – | 1.36E+01 | – | – |
|  | **b** | – | – | – | -3.57E-01 | – | – |
|  | **c** | – | – | – | 1.06E-02 | – | – |
